# A robust dual gene ON–OFF toggle directed by two independent promoter–degron pairs

**DOI:** 10.1242/jcs.260754

**Published:** 2023-04-19

**Authors:** Tsz Kwan Yeung, Sehong Kim, Hoi Tang Ma, Randy Y. C. Poon

**Affiliations:** ^1^ Division of Life Science, Hong Kong University of Science and Technology, Clear Water Bay, Hong Kong; ^2^ Department of Pathology, The University of Hong Kong, Hong Kong; ^3^ State Key Laboratory of Liver Research, The University of Hong Kong, Hong Kong; ^4^ State Key Laboratory of Molecular Neuroscience, Hong Kong University of Science and Technology, Clear Water Bay, Hong Kong

**Keywords:** Auxin-inducible degron, CRISPR, Essential genes, Knockout

## Abstract

Switching genes on and off on cue is a cornerstone for understanding gene functions. One contemporary approach for loss-of-function studies of essential genes involves CRISPR-mediated knockout of the endogenous locus in conjunction with the expression of a rescue construct, which can subsequently be turned off to produce a gene inactivation effect in mammalian cell lines. A broadening of this approach would involve simultaneously switching on a second construct to interrogate the functions of a gene in the pathway. In this study, we developed a pair of switches that were independently controlled by both inducible promoters and degrons, enabling the toggling between two constructs with comparable kinetics and tightness. The gene-OFF switch was based on TRE transcriptional control coupled with auxin-induced degron-mediated proteolysis. A second independently controlled gene-ON switch was based on a modified ecdysone promoter and mutated FKBP12-derived destabilization domain degron, allowing acute and tuneable gene activation. This platform facilitates efficient generation of knockout cell lines containing a two-gene switch that is regulated tightly and can be flipped within a fraction of the time of a cell cycle.

## Teaser

**Summary:** New tools and resources are available for generating tight two-gene on–off switches in mammalian cells.

## INTRODUCTION

Although gene disruption represents the most direct approach in loss-of-function analysis, it is generally irreversible and cannot be used to study essential genes. Tactics including conditional inactivation or depletion are required for studying essential genes. One contemporary approach involves CRISPR-mediated knockout (KO) of the endogenous locus in conjunction with the expression of a rescue construct (itself resistant to the CRISPR-Cas9), which can subsequently be turned off to produce a gene inactivation effect.

Recently, we have demonstrated that combining transcriptional control and degron-mediated post-translational regulation enables both rapid and tight gene silencing in human cells (Ng et al., 2019; Yeung et al., 2021). Transcription is governed by a widely used tetracycline-controlled promoter (Tet-Off) system (Gossen and Bujard, 1992). Concurrently, the protein is targeted for degradation using an auxin-inducible degron (AID) (Hayashi and Karlseder, 2013). The integrated approach addresses some of the shortcomings of the TRE system and AID system, including the ‘leakiness’ and relatively slow responses of the tetracycline-controlled promoters as well as the presence of residual levels of AID fusion proteins after degradation is induced. This allows depletion of essential genes including cyclin-dependent kinase 1 (CDK1) to <1% of the endogenous levels (Lau et al., 2021).

A more powerful approach for gene analysis would involve switching on a second construct at the same time as the rescue construct is turned off. Examples of the induced construct could include mutated versions of the original gene, different isoforms or another gene altogether. This could provide an extra level of analysis of the extent that the induced gene can compensate for the functions of the disrupted gene. Nonetheless, effective and robust systems that can toggle between two genes rapidly in mammalian cells still remains to be established.

**Fig. 1.**
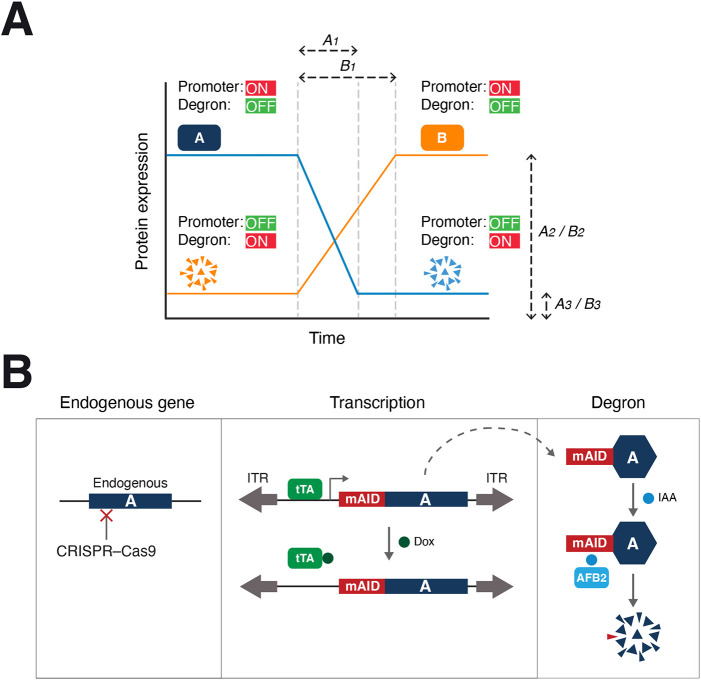
**A dual gene ON–OFF switch.** (A) Elements of a dual gene ON–OFF switch. Protein A is turned off rapidly by silencing the promoter and inducing degron-mediated proteolysis. Under the control of a different promoter and degron, protein B is activated independently by turning on the promoter and suppressing the degron. Critical parameters for protein A include achieving an expression similar to the endogenous protein (*A_2_*) and the tightness of the suppression (*A_3_*). Features of protein B should include a low uninduced expression (*B_3_*) and robust activation (*B_2_*). An ideal switch should also be rapid and should have a comparable rate between switching off A (*A_1_*) and turning on B (*B_1_*). (B) Overview of the conditional gene silencing approach. The endogenous locus of a gene (labeled A) is disrupted using CRISPR-Cas9 (note that cancer cell lines might possess multiple copies of the gene). The cDNA of the gene is put inside a SB transposon cassette and delivered to the genome to rescue the KO effects (ITR; inverted terminal repeat). Silence mutations are introduced into the cDNA to render the cDNA resistant to the CRISPR-Cas9. The tetracycline-controlled transcriptional activator (tTA) binds to the TRE in the promoter and activates the transcription of mAID-tagged cDNA in the absence of Dox. Addition of Dox turns off the transcription. In response to IAA, residual mAID fusion protein is targeted for degradation in cells expressing AFB2.

For the construct that is turned on initially (the rescue cDNA), major obstacles include the kinetics and tightness of the inducible suppression (Fig. 1A, *A_1_* and *A_3_*, respectively). Ideally, protein expression should be reduced significantly relative to the endogenous protein and within a fraction of the time of the cell cycle. Likewise, a low uninduced expression is crucial for the gene that is silenced initially (Fig. 1A, *B_3_*). As gene activation involving synthesis of mRNA and/or protein is generally slower than degron-mediated gene inactivation, another major parameter is the switch-on kinetics (Fig. 1A, *B_1_*). Finally, the protein expression achieved during activation of the two genes (Fig. 1A, *A_2_* and *B_2_*), either relative to the endogenous protein before CRISPR-Cas9-mediated KO or with each other, could possibly affect the experimental outcome.

To tackle these challenges, we first refined the tTA–AID dual transcription–degron conditional deficiency system to produce a more responsive gene-OFF switch. This was coupled with a compatible and independently controlled transcription–degron system (DBEcR–DD) for gene-ON activation. This facilitated the generation of KO-cell lines with a two-gene switch that could be flipped rapidly and tightly (<1% protein remaining in the OFF state for both genes). Expression of the two genes was tuneable and could be controlled independently.

## RESULTS

### A conditional gene-OFF system utilizing the tTA promoter and AID

We first further refined the tTA–AID dual transcription–degron system (Ng et al., 2019; Yeung et al., 2021) to reduce background protein turnover (summarized in Fig. 1B). Concurrent with the disruption of the gene of interest with CRISPR-Cas9, an AID-tagged cDNA of the gene (which was resistant to the CRISPR-Cas9 through the introducing silence mutations at the CRISPR-Cas9-targeting site) under the control of a Tet-Off promoter was integrated into the genome using Sleeping Beauty (SB) transposons. Transcription of the cDNA mediated by the tetracycline-controlled transcriptional activator (tTA) can be turned off using doxycycline (Dox). On the other hand, the presence of F-box proteins that target AID can facilitate rapid proteolysis when indole-3-acetic acid (IAA) is added (Nishimura et al., 2009).

The original AID system consists of AID or a minimal functional AID (mAID) (Brosh et al., 2016; Kubota et al., 2013; Morawska and Ulrich, 2013; Yesbolatova et al., 2019) and the *Oryza sativa* F-box protein TIR1. When expressed in mammalian cells, TIR1 can form a complex with an endogenous cullin protein, RBX1 and SKP1 to form a SCF-type ubiquitin ligase (Nishimura et al., 2009). However, a shortcoming of the AID–TIR1 system is the relatively high background turnover rate in the absence of IAA.

Several approaches have been devised to address this issue, including the use of homologous AID–TIR1 pairs from other species. Li et al. found that a minimal *Arabidopsis thaliana* degron IAA7 (mIAA7 herein) can be targeted to degradation by the *A. thaliana* F-box protein AFB2 with relatively low basal degradation (Li et al., 2019; Yunusova et al., 2021). Using the cyclin-dependent kinase CDK2 as an example, we generated HeLa cells stably expressing either ^mAID^CDK2 with TIR1 or ^mIAA7^CDK2 with AFB2. The endogenous CDK2 was at the same time disrupted with CRISPR-Cas9. As expected, both ^mIAA7^CDK2 and ^mAID^CDK2 could be depleted after incubation in medium containing Dox and IAA (DI herein) (Fig. S1A). However, a truncated product from ^mIAA7^CDK2 was present before DI treatment, which was absent in cells expressing ^mAID^CDK2.

Inspection of the aligned sequences revealed that a methionine (Met^52^) is present in mIAA7 instead of a valine in the homologous position in mAID (Val^32^) (Fig. S1B). One possibility is that mIAA7 Met^52^ was responsible for internal initiation during translation. In support of this hypothesis, the truncated product was largely eliminated when Met^52^ was mutated to valine [M52V; mIAA7(MV)] (Fig. S1C). Unlike full-length ^mIAA7^CDK2, the truncated product was not degraded after exposure to IAA (without Dox to silence transcription), further supporting the idea that most of the mIAA7 domain was missing in the truncated product (Fig. S2A). However, trace amounts of truncated products were still detected in the M52V-mutated ^mIAA7^CDK2 cells, especially when compared side-by-side with ^mAID^CDK2 (Fig. S2B,C). Furthermore, the low level of truncated product was present irrespective of whether ^mIAA7(MV)^CDK2 was co-expressed with AFB2 (Fig. S2C) or TIR1 (Fig. S2B). One possibility is that another methionine, such as Met^53^, was used for internal initiation in mIAA7(MV).

**Fig. 2.**
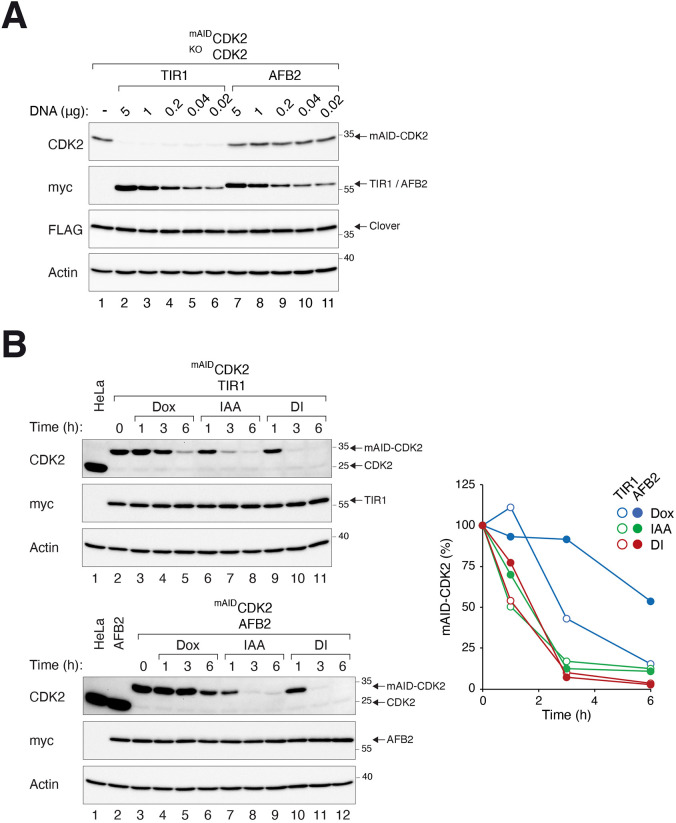
**mAID is effective as a degron in the presence of AFB2.** (A) Lower background degradation of ^mAID^CDK2 by AFB2 compared to TIR1. ^mAID^CDK2-expressing cells (in a CDK2^KO^ background) in 10-cm plates were transfected with different amounts of plasmids expressing TIR1 (plasmid 45) or AFB2 (plasmid 5). After antibiotic selection for 13 days to enrich transfected cells, lysates were prepared for immunoblotting analysis. The cell line also expressed FLAG-Clover, which served as a control for a protein not targeted by TIR1 or AFB2. (B) Gene silencing by combination of the TRE promoter and mAID–AFB2 degron system. CDK2-depleted cells expressing ^mAID^CDK2 and TIR1 (upper panel) or ^mAID^CDK2 and AFB2 (lower panel) were treated with Dox and/or IAA and harvested at the indicated time points. Lysates were prepared and analyzed with immunoblotting. Lysates from HeLa or cells expressing AFB2 alone (HtTB2) were loaded as controls. The signals on the blots were quantified using a serial dilution standard curve and standardized with actin signals (right panel). Blots and graph shown representative of two independent experiments.

In agreement with Li et al. (2019), AFB2 conferred a lower rate of background degradation in ^mAID^CDK2 than TIR1 (Fig. S2D). This was further verified by titrating different amounts of TIR1 or AFB2 into ^mAID^CDK2-expressing cells (Fig. 2A). Expression of TIR1 resulted in considerably lower basal levels of ^mAID^CDK2 in comparison to similar expression of AFB2. As a control, the abundance of FLAG–Clover in the same cell line was unaffected by TIR1 or AFB2. The lower expression of ^mAID^CDK2 in TIR1-expressing cells was not due to transcription, as the mRNA of ^mAID^CDK2 was in fact higher in TIR1- than in AFB2-expressing cells (Fig. S2E). We also found that in the presence of AFB2, the background turnover rate of ^mIAA7(MV)^CDK2 was higher than that of ^mAID^CDK2 (Fig. S2D). This was reflected in the lower protein expression of ^mIAA7(MV)^CDK2 comparing to ^mAID^CDK2 in cell lines, in spite of the higher mRNA expression of the former (Fig. S2E).

Given the issues of truncation and background degradation associated with mIAA7, we concentrated on the combination of mAID with AFB2 and further characterized the system in the context of dual transcription–degron regulation. Consistent with the relatively low background turnover associated with AFB2, ^mAID^CDK2 was reduced more slowly in AFB2 cells compared to in TIR1 cells after turning off the transcription with Dox alone. As we have shown previously (Yeung et al., 2021), ^mAID^CDK2 in TIR1-expressing cells could be silenced with DI, which triggered a more acute and complete depletion compared to that seen with the individual chemicals (Fig. 2B, upper panel). Importantly, ^mAID^CDK2 could be turned off by DI as rapidly in the presence of AFB2 as in the presence of TIR1 (Fig. 2B, lower panel).

Taken together, these results demonstrated that in combination with TRE transcriptional control, mAID can be used effectively with the F-box protein AFB2 to facilitate both rapid and tight gene silencing. This combination circumvents the truncation products associated with mIAA7 and the relatively high background degradation of TIR1. Vectors of the tTA–AID system that are available for generating stable cell lines are summarized in Fig. S3.

### A conditional gene-ON system based on DBEcR promoter and DD degron

**Fig. 3.**
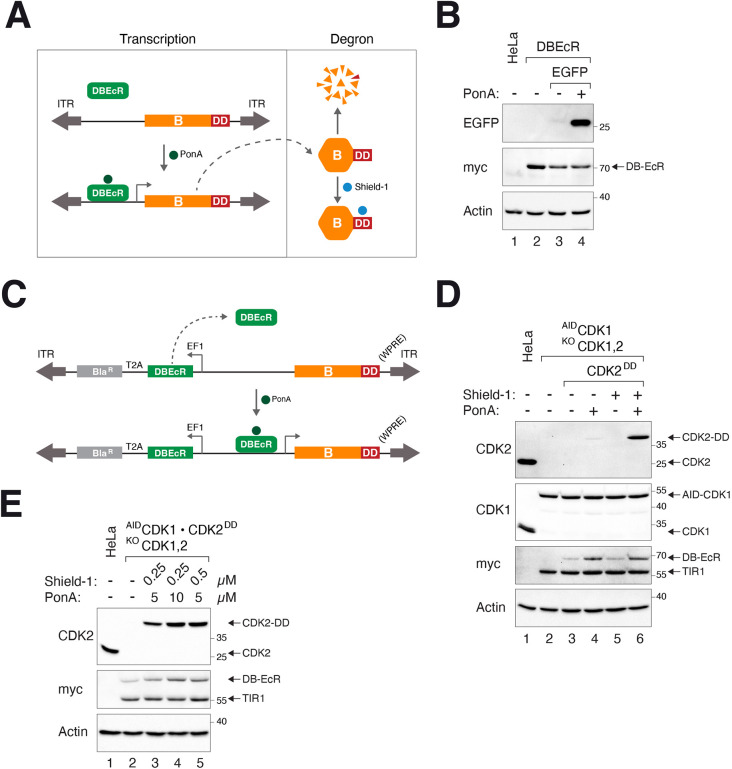
**A conditional gene-ON system using the DBEcR promoter and DD degron.** (A) Overview of the conditional gene activation approach. A DD-tagged gene of interest (labeled B) is put inside a SB transposon cassette and delivered to the genome (ITR, inverted terminal repeat). DBEcR activates a modified ecdysone promoter only in the presence of PonA. Residual protein is targeted for proteolysis by the DD domain. DD-tagged protein is stabilized by the addition of Shield-1. (B) Activation of DBEcR-mediated transcription by PonA. EGFP under the control of DBEcR-responsive promoter (plasmid 24) was transiently transfected into HeLa cells expressing DBEcR (Myc tagged). The cells were incubated with PonA (5 µM) for 24 h before harvested for immunoblotting analysis. (C) Organization of the DD-expressing plasmid. A gene of interest (labeled B) was cloned into a plasmid allowing it to be fused to the DD degron at the C-terminus. Bla^R^, blasticidin-resistance gene; EF1, EF1α promoter; ITR, SB inverted terminal repeat; T2A, T2A ribosomal skipping site. In some cases, a version of plasmid containing WPRE at the 3'-untranslated region was used to increase mRNA stability. (D) Synergistic action of PonA and Shield-1 on gene activation. CDK2^DD^-expressing cells (in a ^AID^CDK1, CDK1^KO^ or CDK2^KO^ background) were incubated with PonA (5 µM) and/or Shield-1 (0.25 µM). The cells were harvested after 24 h for immunoblotting analysis. (E) Tuneable activation of CDK2^DD^. CDK2^DD^-expressing cells (as in D) were incubated with different combination of PonA and Shield-1 for 24 h. Blots shown representative of two independent experiments.

Fig. 3A shows the setup of the dual transcription-degron gene-ON system. The inducible promoter was based on a hybrid *Drosophila*/*Bombyx* ecdysone receptor (DBEcR), which can transactivate a modified ecdysone promoter in the presence of the ecdysone agonist Ponasterone A (PonA) (Hoppe et al., 2000). The protein of interest is tagged with a mutated FK506-binding protein-12 (FKBP12; also known as FKBP1A)-derived destabilization domain (DD) to target it to proteolysis. DD-fused proteins can be stabilized by binding to the small molecule Shield-1 (Banaszynski et al., 2006). Addition of PonA and Shield-1 together (PS herein) increases transcription and protein stability, respectively.

We first generated cells that expressed EGFP under the control of DBEcR promoter and confirmed PonA-dependent induction of EGFP (Fig. 3B). The presence of detectable EGFP before PonA addition, however, suggested that additional control is required to obtain a tighter gene suppression. The DBEcR cassette was then put into the same plasmid that expressed DD-tagged proteins (Fig. 3C). The entire cassette (which also contained a blasticidin-resistance selection marker) was delivered into cells using the SB transposon.

To evaluate whether the DD tag indeed helps to suppress gene expression before induction, EGFP and EGFP^DD^ were put under the control of the DBEcR promoter and transfected into cells. Although both EGFP and EGFP^DD^ could be induced to a similar level, the background expression of EGFP was significantly higher in the absence of the DD tag (Fig. S4A). This indicates that the combination of inducible promoter and DD degron helped to maintain a low ‘leakiness’ while allowing strong induction.

Using CDK2 as a model once more, a cell line expressing CDK2^DD^ and ^AID^CDK1 in a CDK1^KO^CDK2^KO^ double-KO background was generated. Fig. 3D shows that whereas CDK2^DD^ only accumulated marginally when the cells were exposed to PonA or Shield-1, it was strongly activated when both chemicals were added together. Interestingly, although DBEcR was under the control of a constitutively active EF1α promoter, its level was also elevated by PonA (Fig. 3D). One possibility is that the modified ecdysone promoter was also able to promote the transcription of the nearby DBEcR. Notwithstanding, this might help to further enhance the inducible expression of DD-tagged proteins.

We next tested different concentrations of PonA and Shield-1 to determine whether graded induction of DD-tagged proteins could be achieved. CDK2^DD^ was only marginally induced by PonA alone or stabilized by Shield-1 alone at the maximum concentrations used (Fig. S4B). Significantly, high and dose-dependent expression of CDK2^DD^ could be achieved when PonA and Shield-1 were used together (Fig. 3E). Furthermore, Fig. S4C shows that after turning on CDK2^DD^ using PS (in a cell line with the CDK2^KO^ background), the expression of CDK2^DD^ did not diminish significantly over the next 10 days without replenishing PS, suggesting that the gene-ON system can be sustained over a long period of time.

**Fig. 4.**
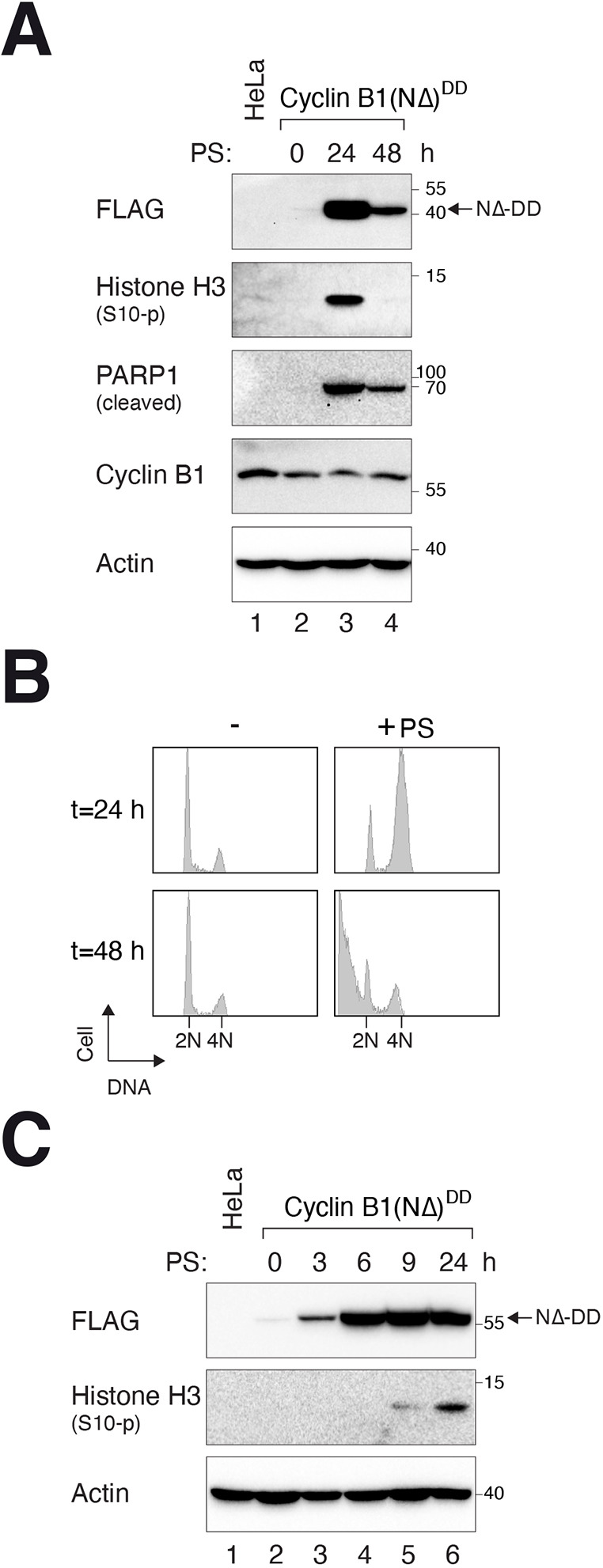
**Tight regulation of the DBEcR–DD gene-ON system.** (A) Activation of cyclin B1(NΔ)^DD^. HeLa cells expressing DBEcR and cyclin B1(NΔ)^DD^ were generated. The cells were incubated with PS and harvested at the indicated time points for immunoblotting analysis. Note that the cyclin B1(NΔ)^DD^ was detected with antibodies against FLAG tag as it was not recognized by our monoclonal antibody against cyclin B1. (B) Expression of cyclin B1(NΔ)^DD^ induces mitotic arrest and apoptosis. Cells were treated with PS as in A. At different time points, the cells were harvested for flow cytometry analysis. The positions of 2N and 4N DNA contents are indicated. (C) Rapid accumulation of cyclin B1(NΔ)^DD^. Cells were incubated with PS and harvested at the indicated time points for immunoblotting analysis. Blots and flow cytometry analysis representative of three independent experiments.

To evaluate whether the gene-ON system was sufficiently tightly suppressed before induction for practical use, a cytotoxic cDNA [an N-terminally truncated (NΔ88) cyclin B1 lacking the D-box] was cloned into the DD-tagged vector. As cyclin B1(NΔ) cannot be degraded by the anaphase-promoting complex or cyclosome (APC/C), its expression results in a mitotic block (Wolf et al., 2006). Indeed, stable cell lines that conditionally expressed cyclin B1(NΔ)^DD^ could be generated. Cyclin B1(NΔ)^DD^ was robustly induced with PS and blocked cells in mitosis, as indicated by the accumulation of histone H3^Ser10^ phosphorylation (Fig. 4A) and G_2_/M DNA contents (Fig. 4B). Apoptosis associated with prolonged mitotic block was indicated by the accumulation of cleaved PARP1 and sub-G_1_ DNA. The induction of cyclin B1(NΔ)^DD^ was relatively rapid, accumulating to a saturated level at ∼6 h after addition of PS (Fig. 4C).

Finally, we found that for some cDNAs [e.g. cyclin B1(NΔ)^DD^], more sustained activation of the DD-tagged proteins could be achieved by appending a woodchuck hepatitis virus posttranscriptional regulatory element (WPRE) to the 3′-untranslated region to increase mRNA stability (Donello et al., 1998). Versions of DD-containing vectors with or without WPRE were generated to provide flexibility to express different genes (Fig. S5).

Collectively, these data show that the DBEcR–DD gene-ON system can achieve robust and tuneable gene activation with a low uninduced expression. Fig. S5 summarizes the SB vectors of the DBEcR–DD system available for generating stable cell lines.

### Combining gene-ON and gene-OFF systems for rapid gene switching

**Fig. 5.**
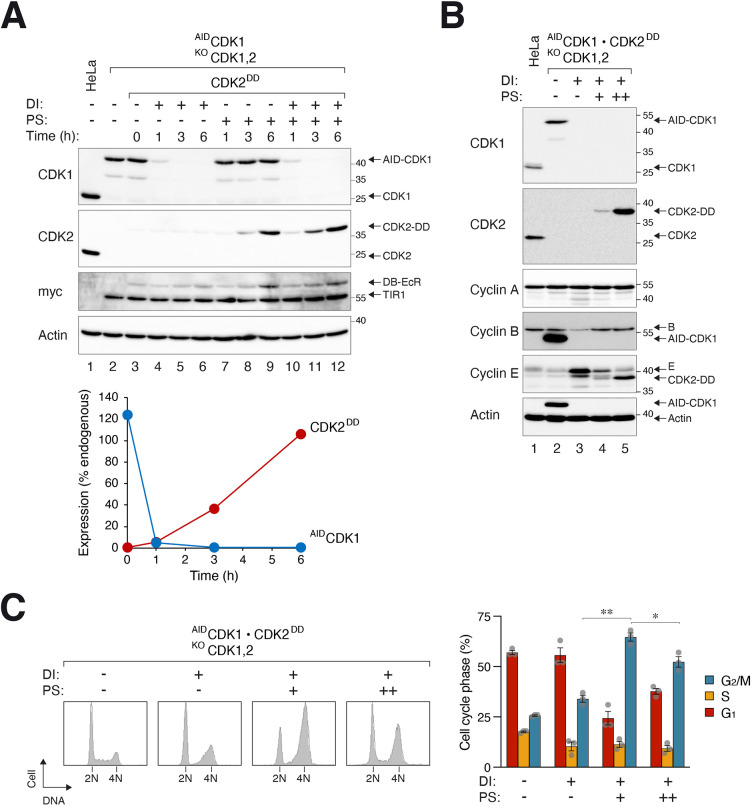
**Combination use of the gene ON–OFF switch demonstrates the essential function of CDK1 can be replaced by overexpressed CDK2.** (A) CDK gene ON–OFF switch. HeLa cells lacking endogenous CDK1 and CDK2 were generated by expressing ^AID^CDK1 under the control of the gene-OFF (tTA–AID) system and CRISPR-Cas9 against CDK1 and CDK2. CDK2^DD^ under the control of the gene-ON (DBEcR–DD) system was further engineered into the cell line. The cells were incubated with DI and PS (to turn off ^AID^CDK1 and turn on CDK2^DD^, respectively) and harvested at the indicated time points for immunoblotting analysis. The parental cells (HeLa and ^AID^CDK1-expressing cells) were also loaded as controls. The signals on the immunoblots were quantified using densitometry with serial dilution standard curves and standardized with actin signals (lower panel). (B) The use of the DBEcR–DD system to express different levels of CDK2. CDK1 and CDK2 double-KO cells capable of expressing ^AID^CDK1 and/or CDK2^DD^ were as described in A. The cells were treated with DI and/or PS (+, 5 µM PonA and 0.25 µM Shield-1; ++, 10 µM PonA and 2.5 µM Shield-1) for 24 h. Lysates were prepared and analyzed with immunoblotting. Blots shown in A and B representative of two independent experiments. (C) Overexpression of CDK2 can complement the cell cycle functions of CDK1. ^AID^CDK1 and/or CDK2^DD^ were expressed in CDK1- and CDK2-deficient cells as described in B before being analyzed with flow cytometry. Although the G_1_ arrest caused by CDK1 deficiency (with DI) could be rescued with low expression of CDK2^DD^, the G_2_/M arrest was reduced only in the presence of overexpressed CDK2^DD^. The distribution of different cell cycle phases was quantified (left hand panel). Mean±s.e.m. from three independent experiments. **P<0.01; *P<0.05 (Mann–Whitney test).

To test the gene-ON and gene-OFF systems within the same cell line, CDK1 and CDK2 were placed under the control of the tTA–AID and the DBEcR–DD system, respectively. As the endogenous CDK1 and CDK2 loci were disrupted with CRISPR-Cas9, switching off ^AID^CDK1 yielded cells containing neither CDK1 nor CDK2. Alternatively, activation of CDK2^DD^ produced cells containing both CDK1 and CDK2. Finally, treating the cells with DI and PS together enabled a switch from a CDK1-only to a CDK2-only environment. Before DI treatment, ^AID^CDK1 was expressed to a level similar to that of the endogenous CDK1 (Fig. 5A, compare lanes 1 and 3). Gene silencing was relatively rapid, with ∼5% of the ^AID^CDK1 remaining after 1 h (and below the detectable range by 3 h). CDK2^DD^ was activated and reached a level comparable to the endogenous CDK2 after 5 h. Although four chemicals (Dox, IAA, PonA and Sheild-1) were involved in toggling the gene ON–OFF switch, colony formation assays indicated that they exerted no significant effect on long-term survival in HeLa (Fig. S6A) or RPE1 (Fig. S6B) cells.

To determine whether the system was tuneable, we next used different concentrations of PS to control the relative abundance of CDK2^DD^. By varying the concentration of PonA and Shield-1, CDK2^DD^ could be stabilized either to levels lower or higher than the endogenous CDK2 (Fig. 5B).

Finally, the reversibility of the gene switch was examined by using a cell line that expressed ^mAID^CDK2 and CDK2^DD^. ^mAID^CDK2 could be switched to CDK2^DD^ after addition of DI and PS together (Fig. S6C). After washing out the chemicals, CDK2^DD^ expression returned to basal expression between 3–6 h (Fig. S6D). Resetting the ^mAID^CDK2 expression took longer, requiring 9–24 h for ^mAID^CDK2 to re-accumulate to its original level.

Collectively, these results show that the gene-ON and gene-OFF systems can be independently controlled in the same cell line, are tuneable (for gene-ON) and can be reset to their initial state.

### Independent switches of CDK1 and CDK2 validates a quantitative model of CDK control of the cell cycle

We recently showed that although cells can progress through the cell cycle normally in the absence of CDK2, they undergo mitosis with massive chromosome alignment defects without CDK1. These findings indicated that CDK1 can compensate for the loss of CDK2 but not vice versa (Lau et al., 2021). Overexpression of CDK2, however, can compensate for the loss of CDK1, giving support of a quantitative model of CDK function in the cell cycle (Fig. S6E).

By placing CDK1 and CDK2 in the gene ON–OFF system, we found that cells lacking endogenous CDK1 and CDK2 and expressing ^AID^CDK1 (with CDK2^DD^ turned off) displayed a normal cell cycle profile (Fig. 5C). Turning off the ^AID^CDK1 induced cell cycle arrest in both G_2_/M and G_1_/S (as cells contain neither CDK1 nor CDK2). Turning on CDK2^DD^ to a level lower than the endogenous CDK2 overcame the G_1_/S but not the G_2_/M cell cycle arrest caused by the absence of CDK1. By contrast, overexpression of CDK2^DD^ (by using higher concentrations of PonA and Shield-1) was able to reduce the G_2_/M delay as well.

These data demonstrated that the tuneable gene ON–OFF switch can facilitate the switching between two genes with overlapping functions and provided further evidence to support the quantitative CDK model for cell cycle control.

### Toggling between wild-type and a kinase-dead mutant reveals the importance of CDK2

The platform developed here could facilitate the switching from a wild-type (WT) to an inactive form of an enzyme. CDK2^KO^ cells were engineered to express ^mAID^CDK2 initially. Either a WT or a kinase-dead mutant (K33R) of CDK2^DD^ was then turned on at the same time as ^mAID^CDK2 inactivation.

Fig. S6F shows that cyclin A (a cyclin that normally interacts with both CDK1 and CDK2; Pagano et al., 1992) switched from binding to ^mAID^CDK2 to CDK2^DD^. As expected, the loss of CDK2 resulted in an increase in endogenous CDK1 binding to cyclin A, consistent with a redistribution of cyclin A to CDK1 in the absence of CDK2. Expression of CDK2^DD^ (WT or K33R) returned the cyclin A–CDK1 complexes to the normal level.

**Fig. 6.**
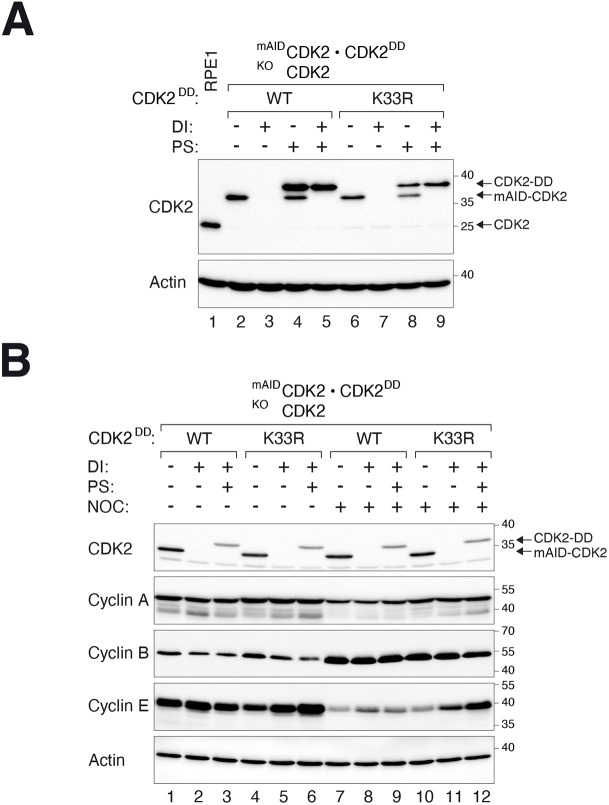
**Switching CDK2 from WT to a kinase-dead mutant in RPE1.** (A) RPE1 cells lacking endogenous CDK2 and expressing ^mAID^CDK2 under the control of the gene-OFF system and CDK2^DD^ (WT or K33R) under the control of the gene-ON system were generated. The cells were incubated with DI and/or PS for 24 h. Lysates were prepared and analyzed with immunoblotting. (B) Replacement of CDK2 with a kinase-inactive mutant impairs S phase progression in RPE1. Cells were generated as described in A. After treating with DI and PS in the presence or absence of nocodazole for 18 h, the cells were harvested for immunoblotting. Blots shown representative of two independent experiments.

Similar cell lines expressing WT or the K33R mutant of CDK2^DD^ were generated using normal epithelial cell line RPE1. As in HeLa cells, ^mAID^CDK2 could be destroyed and CDK2^DD^ turned on independently using DI and PS, respectively (Fig. 6A). To determine whether expression of the K33R mutant could modulate the cell cycle, the expression of different cyclins was examined after ^mAID^CDK2 was switched to CDK2^DD^. Neither the expression of the cyclins nor the cell cycle distribution was altered significantly in asynchronous cells without CDK2 (DI-treated) or expressing CDK2^K33R^ only (DI and PS) (Fig. 6B). Normal cells trapped in mitosis with nocodazole had a low expression of cyclin E and high cyclin B. By contrast, less cyclin B accumulation and more cyclin E were detected in nocodazole-treated CDK2^K33R^-containing cells, suggesting an impairment in S phase progression after acute replacement of CDK2 with CDK2^K33R^.

Collectively, these analyses demonstrated that the gene ON–OFF switch can be used to investigate the functions of different proteins or mutants in both normal and cancer cells.

## DISCUSSION

Gene switches are essential tools for understanding gene functions and in applications including synthetic biology. Dual independent gene ON–OFF switches have numerous potential applications. A rapid and tightly controlled gene-OFF system is well suited for studying essential genes. Various whole-genome studies have estimated that there are 1500 to over 2000 essential genes in human cell lines (Bertomeu et al., 2018; Hart et al., 2015; Wang et al., 2015). As the tTA–AID system described here is already a rescue system by design (Fig. 1B), the effects after the AID-tagged gene is turned off can be confidently attributed to the functions of the gene rather than the off-target effects from CRISPR. The system should also be a versatile tool for investigating the functions of non-essential genes, as the acute loss of a gene avoids the complexity of interpretation caused by possible long-term compensation in response to a gene KO.

In agreement with other studies (Li et al., 2019; Yunusova et al., 2021), we found that AFB2 has a lower background degradation rate for AID than the original TIR1 (Fig. 2). This might prevent an artificially high turnover rate of the AID-tagged proteins. Nonetheless, the relatively high background turnover of AID-tagged proteins in the presence of TIR1 might be useful in some studies, especially when combined with inducible promoters as described here, as it can provide a more rapid silencing of the AID-tagged proteins. Several studies have revealed the advantage of mIAA7 over mAID in terms of degradation dynamics and basal degradation (Li et al., 2019; Yunusova et al., 2021). We prefer the use of mAID over mIAA7 owing to the possible internal translational initiation in mIAA7 (Fig. S2). Moreover, we found that the background degradation of mIAA7 was higher than mAID in the presence of AFB2 (Fig. S2D,E). We have not tested whether tagging mIAA7 to the C-terminus of protein of interest could circumvent the possible internal translational initiation or reduce its background degradation.

After inducible depletion of an essential gene, a second cDNA under the control of the DBEcR–DD system can then be switched on (Fig. 3A). Similar to the tTA–AID gene-OFF system, in which the inducible promoter and degron together produced a more acute and complete depletion compared to either of the elements alone (Fig. 2B), the combination of inducible promoter and DD degron helped to maintain a lower ‘leakiness’ for the DBEcR–DD gene-ON system than the individual elements (Fig. S4A).

One application of the gene switch could be to switch between a WT and a mutant version of the same gene, which might reveal the functionality of the mutant. The effects of KO could be different from inhibiting the activity of the corresponding protein. For example, it is possible that KO of CDK2 does not affect the cell cycle because its cyclin subunits are able to redistribute to bind other CDKs. Here, we generated cell lines that allowed switching from an CDK2 to an CDK2^K33R^-only environment in RPE1 (Fig. 6A) or HeLa (Fig. S6F) cells (and also allowed both CDK2 and CDK2^K33R^ to be expressed together). Our data revealed that the delay in S phase progression in RPE1 cells containing CDK2^K33R^ was more severe than after CDK2 was removed (Fig. 6B).

Another possible application of a gene ON–OFF switch is to examine the relative functions of different isoforms of a gene or closely related genes. For example, we found that CDK2^DD^ could replace the functions of CDK1 after ^AID^CDK1 was switched off (Fig. 6). The tuneable nature of the DBEcR–DD system allowed us to demonstrate that overexpressed but not physiological levels of CDK2^DD^ could compensate for the functions of CDK1 (Fig. 5C).

A shortcoming of the gene ON–OFF system is the relatively slow accumulation of DD-tagged proteins compared to the rapid destruction of mAID-tagged proteins (*B_1_* versus *A_1_* in Fig. 1A). This is not unexpected as *de novo* synthesis of mRNA and protein is generally slower than degron-mediated gene inactivation. Because the DBEcR–DD system was designed with a low uninduced expression in mind (*B_3_* in Fig. 1A), a compromised feature was the relatively slow activation rate. For the example of CDK1 and CDK2, there was a window of several hours when ^AID^CDK1 was destroyed and CDK2^DD^ had not yet accumulated to a level similar to the endogenous CDK2 (Fig. 5A). A similar gap was observed in another cell line after turning off ^mAID^CDK2 and turning on CDK2^DD^ (Fig. S6C). As the gene ON–OFF switch can be controlled independently, one possible solution is to delay the addition of DI for several hours to allow DD-tagged proteins to accumulate first.

Another possible shortcoming of the gene ON–OFF system is the relative inefficient reversibility. In our hands, the switches could be reset by washing out the chemicals (DI and PS). Although some DD-tagged proteins could be turned off within a few hours after PS was removed (Fig. S6D), other DD-tagged proteins, including cyclin B1(NΔ), required more than 8 h to return to basal expression (our unpublished data). The reversibility of the tTA–AID system was generally slow (Fig. S6D). As excessive DI was used in our studies to achieve rapid and tight gene switch off, it is possible that reducing the concentration of DI could allow a more rapid reset of mAID-tagged proteins to basal expression after washing.

The precise maximum expression of the degron-tagged constructs should be determined empirically (which in turns affects the background expression and time to reach a threshold). An advantage of the SB transposon-based strategy described here in comparison to tagging degrons onto endogenous genes includes the avoidance of integration into the endogenous loci using homologous recombination, which is generally inefficient. This is particularly useful for studies involving cancer cell lines because they frequently possess multiple gene copies. This also allows the titration of different amounts of plasmids in the initial transfection to obtain clones with desired expression. Expression can further be controlled by increasing mRNA stability using versions of the plasmids containing WPRE (Fig. S5). It is also possible that a different expression level of the gene-ON relative to the gene-OFF construct is required for some applications (*B_2_* versus *A_2_*, respectively in Fig. 1A). For example, by varying the concentration of PonA and Shield-1, we demonstrated that CDK2^DD^ could be expressed to a level either lower or higher than the endogenous CDK2 (Fig. 5B). Only overexpressed CDK2 could overcome the cell cycle defects caused by CDK1 depletion (Fig. 5C). As with other degrons in general, it is possible that protein functions are affected by the presence of the degrons. Whether it is more suitable to tag the degrons at the at N- or C-terminus of the proteins should be determined empirically for each case.

In this study, we have constructed a series of plasmids with different antibiotic selection markers that can be used to generate both gene-OFF and gene-ON cells (Figs S3, S5). Although it is possible to perform a single transfection with all the constructs together to generate gene ON–OFF cell lines, we generally first created cell lines (from HeLa or RPE1 cells) that stably expressed tTA and AFB2. This workflow facilitated a more direct comparison of expression of different clones after the subsequent integration of degron-tagged gene of interest.

Collectively, by leveraging the power of two independent inducible promoter–degron pairs, we have designed a robust dual gene ON–OFF switch. Rapid and tightly controlled gene switches should have many applications including gene function analysis and synthetic biology.

## MATERIALS AND METHODS

### Plasmids and oligonucleotides

See Table S1 for a list of plasmids, and Table S2 for a list of oligonucleotides.

### Cell lines

RPE1 (hTERT-immortalized retinal pigment epithelial) cells were obtained from American Type Culture Collection (Manassas, VA, USA). Unless stated otherwise, HeLa (cervical carcinoma) cells used in this study were a clone expressing tTA (Yam et al., 2000). AFB2 and histone H2B–Clover were integrated into the AAVS1 safe harbor locus by transfecting HeLa with pAFB2-H2B-Clover/Zeo (plasmid 14), pCas9-sgAAVS1-1 (plasmid 48), pCas9-sgAAVS1-2 (plasmid 49), and a plasmid expressing puromycin-resistant gene (plasmid 51). Cells were selected with puromycin (see below for details of selection drugs used) for 48 h to enrich the transfected cells, followed by selection with zeocin for 2 weeks. Clover-positive cells were enriched using flow cytometry before single clones were isolated by limiting dilution in 96-well plates. The resulting HeLa cell line expressing AFB2 and histone H2B–Clover was called HtTB2.

CDK2^KO^ cells expressing ^mAID^CDK2 and TIR1 (or AFB2) were generated by transfecting HeLa cells with mAID-CDK2 in pUHD-SB-mAID/Hyg (plasmid 2), CDK2 CRISPR-Cas9 (plasmid 46), SB transposase (plasmid 47), pSBbi-TIR1/Pur (plasmid 45) [pSBbi-AFB2/Pur (plasmid 5) for AFB2] and a plasmid expressing a blasticidin-resistant gene (plasmid 50). Cells were selected with blasticidin for 36 h to enrich the transfected cells, followed by selection with hygromycin B and puromycin for 2 weeks. Alternatively, CDK2^KO^ cells expressing ^mAID^CDK2 and AFB2 were generated by transfecting HtTB2 with mAID-CDK2 in pUHD-SB-mAID/Hyg (plasmid 2), CDK2 CRISPR-Cas9 (plasmid 46), and SB transposase (plasmid 47). After 72 h, the cells were selected with hygromycin B for 2 weeks (for Fig. 2B).

CDK2^KO^ cells expressing AFB2 and ^mIAA7^CDK2 or ^mIAA7(MV)^CDK2 were generated by transfecting cells with mAID-CDK2 in pUHD-SB-mAID/Hyg (plasmid 2) or mIAA7(MV)-CDK2 in pUHD-SB-mIAA7(MV)/Hyg (plasmid 4), respectively, pSBbi-AFB2/Pur (plasmid 5), CDK2 CRISPR-Cas9 (plasmid 46), SB transposase (plasmid 47), and a plasmid expressing a blasticidin-resistant gene (plasmid 50). Cells were selected with blasticidin for 36 h to enrich the transfected cells, followed by selection with hygromycin B and puromycin for 2 weeks. CDK2^KO^ cells expressing ^mIAA7^CDK2 and TIR1 were generated similarly except that pSBbi-TIR1/Pur (plasmid 45) was used instead of pSBbi-AFB2/Pur.

CDK2^KO^ cells expressing ^mAID^CDK2 (without TIR1) were generated by transfecting cells with mAID-CDK2 in pUHD-SB-mAID/Hyg (plasmid 2), CDK2 CRISPR-Cas9 (plasmid 46), pSBbi-mRuby2-Clover/Bla (plasmid 44), and SB transposase (plasmid 47). Hygromycin B and blasticidin were added at 72 h after transfection. After 2 weeks, single clones were isolated with limiting dilution in 96-well plates.

Cells expressing DBEcR were generated by transfecting HeLa with pDBEcR/Bla (plasmid 23) and SB transposase (plasmid 47). After 72 h, the cells were selected with blasticidin for 2 weeks.

Cells expressing CDK2^DD^ and ^AID^CDK1 in a CDK1,2^KO^ background (together with TIR1) were generated by transfecting ^AID^CDK1•CDK1,2^KO^ cells (Lau et al., 2021) with CDK2-DD in pDBEcR-pIND(SP1)/Bla (plasmid 29) and SB transposase (plasmid 47). After 72 h, the cells were selected with blasticidin for 2 weeks.

HeLa expressing CDK2^DD^ were generated by transfecting HtTB2 cells with CDK2-DD in pDBEcR-pIND(SP1)/Bla (plasmid 29) and CDK2 CRISPR-Cas9 (plasmid 46), SB transposase (plasmid 47). After 72 h, the cells were selected with blasticidin for 2 weeks.

HeLa expressing CDK2^DD^ or CDK2 (K33R)^DD^ and ^mAID^CDK2 in a CDK2^KO^ background (together with AFB2) were generated by transfecting CDK2^KO^ cells expressing ^mAID^CDK2 and AFB2 (from HtTB2; see above) with CDK2-DD in pDBEcR-pIND(SP1)/Bla (plasmid 29) or CDK2 (K33R)-DD in pDBEcR-pIND(SP1)/Bla (plasmid 30), respectively, and SB transposase (plasmid 47). After 72 h, the cells were selected with blasticidin for 2 weeks.

Cyclin B1(NΔ)^DD^-expressing cells were generated by transfecting HtTB2 with FLAG-3C-cyclin B1(NΔ88)-DD in pDBEcR-pIND(SP1)-DD-W/Bla (plasmid 37) and SB transposase (plasmid 47). After 72 h, the cells were selected with blasticidin for 2 weeks.

RPE1 expressing tTA and AFB2 were generated by transfecting RPE1 with pSBbi-AFB2-tTA/Neo (plasmid 54) and SB transposase (plasmid 47). After 8 days, the cells were selected with neomycin for 2 weeks. Single clones were isolated by limiting dilution in 96-well plates.

RPE1 cells expressing CDK2^DD^ or CDK2 (K33R)^DD^ and ^mAID^CDK2 in a CDK2^KO^ background (together with AFB2) were generated by transfecting tTA-AFB2-expressing RPE1 cells (see above) with mAID-CDK2 in pUHD-SB-mAID/Pur (plasmid 53), CDK2-DD in pDBEcR-pIND(SP1)/Bla (plasmid 29) or CDK2 (K33R)-DD in pDBEcR-pIND(SP1)/Bla (plasmid 30), respectively, and SB transposase (plasmid 47). After 72 h, the cells were selected with blasticidin and puromycin for 2 weeks.

### Cell culture

HeLa cells were propagated in Dulbecco's modified Eagle's medium (DMEM) supplemented with 10% (v/v) calf serum and 50 U/ml of penicillin-streptomycin (Thermo Fisher Scientific, Waltham, MA, USA). RPE1 cells were propagated in DMEM-F12 medium supplemented with 10% (v/v) fetal bovine serum, 50 U/ml of penicillin-streptomycin, and 10 mg/ml of hygromycin B. Cells were cultured in humidified incubators at 37°C with 5% CO_2_.

Unless stated otherwise, cells were treated with the following reagents at the indicated final concentration: blasticidin (Thermo Fisher Scientific; for HeLa cells, 3.75 µg/ml for transient selection and 2.5 µg/ml for stable selection; for RPE1 cells, 7.5 µg/ml), CHX (Sigma-Aldrich, St. Louis, MO, USA; 10 µg/ml), doxycycline (Dox) (Sigma-Aldrich; 2 µg/ml), hygromycin B (Thermo Fisher Scientific; 0.25 mg/ml), indole-3-acetic acid (IAA) (Sigma-Aldrich; 50 µg/ml), neomycin (Santa Cruz Biotechnology, Santa Cruz, CA, USA; 0.75 mg/ml), Ponasterone A (Santa Cruz Biotechnology, Santa Cruz, CA, USA; 5 µM), puromycin (Sigma-Aldrich; for HeLa cells, 0.75 µg/ml for transient selection and 0.3 µg/ml for stable selection; for RPE1 cells, 1.5 µg/ml), Shield-1 (0.5 µM; AOBIOUS, Gloucester, MA, USA), and zeocin (Thermo Fisher Scientific; 0.04 mg/ml).

HeLa cells were transfected using a calcium phosphate precipitation method (Ausubel et al., 1995). RPE1 was transfected using PolyJet *in vitro* DNA transfection reagent (SignaGen Laboratories, Frederick, MD, USA) or Lipofectamine 3000 (Thermo Fisher Scientific). Cell-free extracts were prepared as previously described (Poon et al., 1995). Briefly, cells were harvested by trypsinization and centrifugation. After washed with PBS and collected by centrifugation, the cells were mixed with twice the pellet volume of a lysis buffer (50 mM Tris-HCl pH 7.5, 250 mM NaCl, 5 mM EDTA, 50 mM NaF, 0.2% NonidetP-40, 1 µg/ml leupeptin, 2 µg/ml aprotinin, 15 µg/ml benzamidine, 10 µg/ml pepstatin, and 10 µg/ml soybean trypsin inhibitor). The suspension was incubated at 4°C for 30 min and cell debris was removed by centrifugation in a microfuge at 4°C for 30 min. Protein concentration of the cell lysates was measured with bicinchoninic acid protein assay system (Thermo Fisher Scientific) using bovine serum albumin (BSA) as a standard.

For colony formation assay, 200 cells were seeded onto 60-mm plates and treated with the indicated chemicals. After 14 days, colonies were fixed with methanol:acetic acid (2:1 v/v) and stained with 2% (w/v) Crystal Violet (Sigma-Aldrich) for visualization.

### Quantitative real-time PCR

Total RNA extraction, reverse transcription PCR, and real-time PCR were performed as previously described (Ng et al., 2019), except that primers against mAID- and mIAA7(MV)-tagged CDK2 (5′-AAGTTGTACCTCCCCTGGAT-3′ and 5′-GTACCGAGCTCGAATTCCTA-3′) were used for real-time PCR.

### Flow cytometry

Flow cytometry analysis after propidium iodide staining was performed as previously described (Mak et al., 2020). Briefly, cells were trypsinised and washed with PBS. The cells were then fixed with ice-cold 70% ethanol and stained with a solution containing 40 µg/ml of propidium iodide and 40 µg/ml of RNaseA at 37°C for 30 min. DNA contents of 5000–10,000 cells were analyzed with FACSAria™ III (BD Biosciences, Franklin Lakes, NJ, USA).

### Antibodies and immunoblotting

Immunoblotting was performed as previously described (Ng et al., 2019). Briefly, samples were separated on SDS-PAGE before transferring to Immobilon PVDF membrane (Merck Millipore, Darmstadt, Germany). The membrane was blocked with 10 mM Tris-HCl pH 8.0, 150 mM NaCl and 0.05% Tween-20 (TBST) containing 4% dry milk or 1% BSA at 25°C for 30 min. The membrane was incubated with primary antibodies (see below) in TBST 2% dry milk or 1% BSA at 4°C overnight. After washing with TBST, the membrane was incubated with horseradish peroxidase-conjugated anti-mouse or anti-rabbit immunoglobulin (Thermo Fisher Scientific) at 25°C for 2 h. The membrane was then washed three times with TBST, developed using ECL chemiluminescence, and detected using a ChemiDoc Touch imaging system (Bio-Rad, Hercules, CA, USA). The positions of molecular size standards (in kDa) are indicated in the Figures. Quantification of signals on immunoblotting was conducted with Image Lab software (version 5.1 build 8, Bio-Rad Laboratories, Hercules, CA, USA).

Immunoprecipitation was performed as previously described (Lau et al., 2021). Briefly, 900 µg of cell extracts were incubated with 1.5 µg of purified antibodies at 4°C for 2 h. The reaction was diluted with 200 µl of bead buffer (25 mM Tris-HCl pH 7.4, 2.5 mM NaF, 125 mM NaCl, 2.5 mM EDTA, 2.5 mM EGTA, 0.05% Nonidet P-40, 1 µg/ml aprotinin, 7.5 µg/ml benzamidine, 0.5 µg/ml leupeptin, and 5 µg/ml soybean trypsin inhibitor) and added to 10 µl of protein A/G PLUS-Agarose (Santa Cruz Biotechnology). After incubation at 4°C for 60 min with end-over-end rotation, the beads were spun down in a microfuge and washed four times (1 ml each time) with bead buffer. The beads were then mixed with 50 µl of SDS-sample buffer and boiled for 5 min.

The following antibodies were obtained from the indicated sources: monoclonal antibodies against β-actin (A5316, Sigma-Aldrich, 1:40,000), CDK1 (sc-54, Santa Cruz Biotechnology, 1:1000), CDK2 (sc-6248, Santa Cruz Biotechnology, 1:1000; or ab32147, Abcam, Cambridge, UK, 1:1000), cyclin A2 (AT10, a gift from Tim Hunt, Cancer Research UK, 1:1000), cyclin B1 (sc-245, Santa Cruz Biotechnology, 1:1000), cyclin E1 (sc-247, Santa Cruz Biotechnology, 1:1000), GFP (sc-9996, Santa Cruz Biotechnology, 1:1000), FLAG (F1804, Sigma-Aldrich, 1:1000), MYC (sc-40, Santa Cruz Biotechnology, 1:1000), cleaved PARP1 (552597, BD Biosciences, 1:10,000); PSTAIRE (a gift from Masakane Yamashita, Hokkaido University, Japan, 1:1000); and polyclonal antibodies against CDK1 (Li et al., 2002, 1:1000) and phosphorylated histone H3^Ser10^ (sc-8656R, Santa Cruz Biotechnology, 1:1000).

## Supplementary Material

10.1242/joces.260754_sup1 Supplementary information
