# Supplementary material for: A robust dual gene ON–OFF toggle directed by two independent promoter–degron pairs
Source: J Cell Sci. 2023 Apr 19;136(8):jcs260754. doi: 10.1242/jcs.260754 (PMC10198621; doi:10.1242/jcs.260754)
Supplement: Supplementary information [file joces-136-260754-s1.pdf]

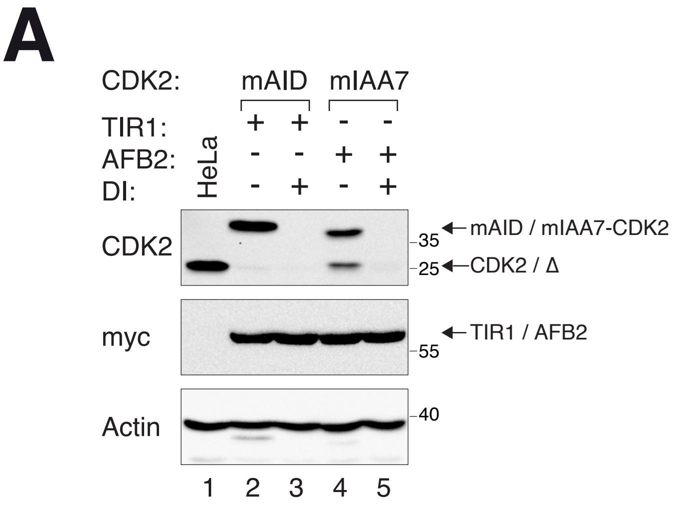

B

```
mAID                 MAGAKEKSACPK------DPAKPPAKAQVVGWPPVRSYRK
mIAA7   MLNLQSNKEGSVDLKNVSAVPKEKTTLKDPSKPPAKAQVVGWPPVRNYRK

mAID    NVMVSCQKSSGGPEAAAFVKVSMDGAPYLRKIDLRMYKASMAREFELGTR
mIAA7   NMMTQQKTSS------------------------------MAREFELGTR

mAID    GSRHDKIDRHDKIH
mIAA7   GSRHDKIDRHDKIH
```

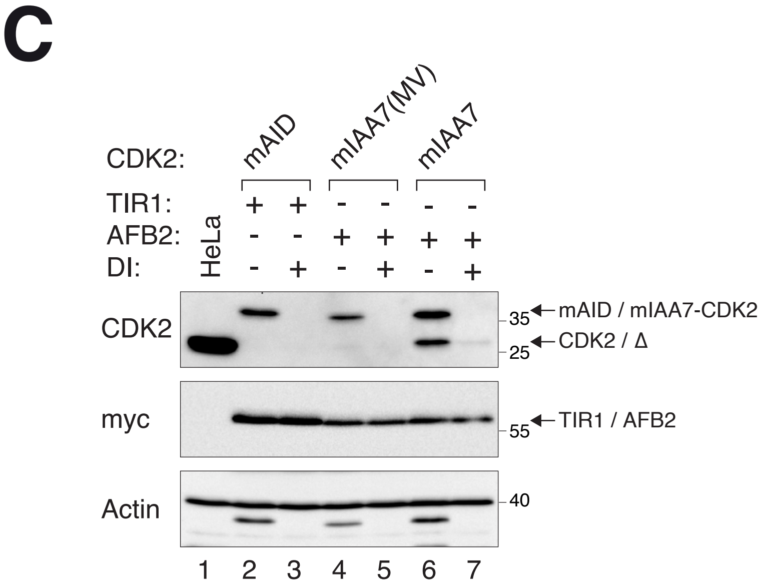

Journal of Cell Science • Supplementary information

Fig. S1. An improved gene-OFF system involving transcriptional control and AFB2-mediated degradation of mAID.

(A) Aberrant expression of truncated products from the mIAA7 degron. HeLa cells stably expressing either ^{mAID}CDK2 or ^{mIAA7}CDK2 together with TIR1 or AFB2 (both myc-tagged), respectively, were generated. The endogenous CDK2 was at the same time disrupted with CRISPR-Cas9. The cells were cultured in either the presence or absence of Dox and IAA (DI) for 24 h to silence the degron-tagged CDK2. Lysates were prepared and analyzed with immunoblotting. Lysates from HeLa were also loaded to indicate the expression of endogenous CDK2. Note the presence of a truncated product from ^{mIAA7}CDK2 (Δ), which is slightly bigger than endogenous CDK2. Equal loading of lysates was confirmed by immunoblotting for actin.

(B) mIAA7 possesses an internal methionine that is absent in mAID. Alignment of the amino acid sequences between mAID and mIAA7 is shown. Identical amino acids are highlighted in grey. Met^{52} of mIAA7 and Val^{36} in mAID are highlighted in yellow.

(C) Mutation of Met^{52} largely abolishes the expression of truncated products from mIAA7. Point mutation M52V was introduced into mIAA7 to generate mIAA7(MV). Cell lines expressing mAID-, mIAA7-, or mIAA7(MV)-tagged CDK2 in combination with TIR1 or AFB2 were either untreated or incubated with DI for 24 h before analyzed with immunoblotting. The lower bands in the actin blot were signals from the previous CDK2 blot.

Journal of Cell Science • Supplementary information

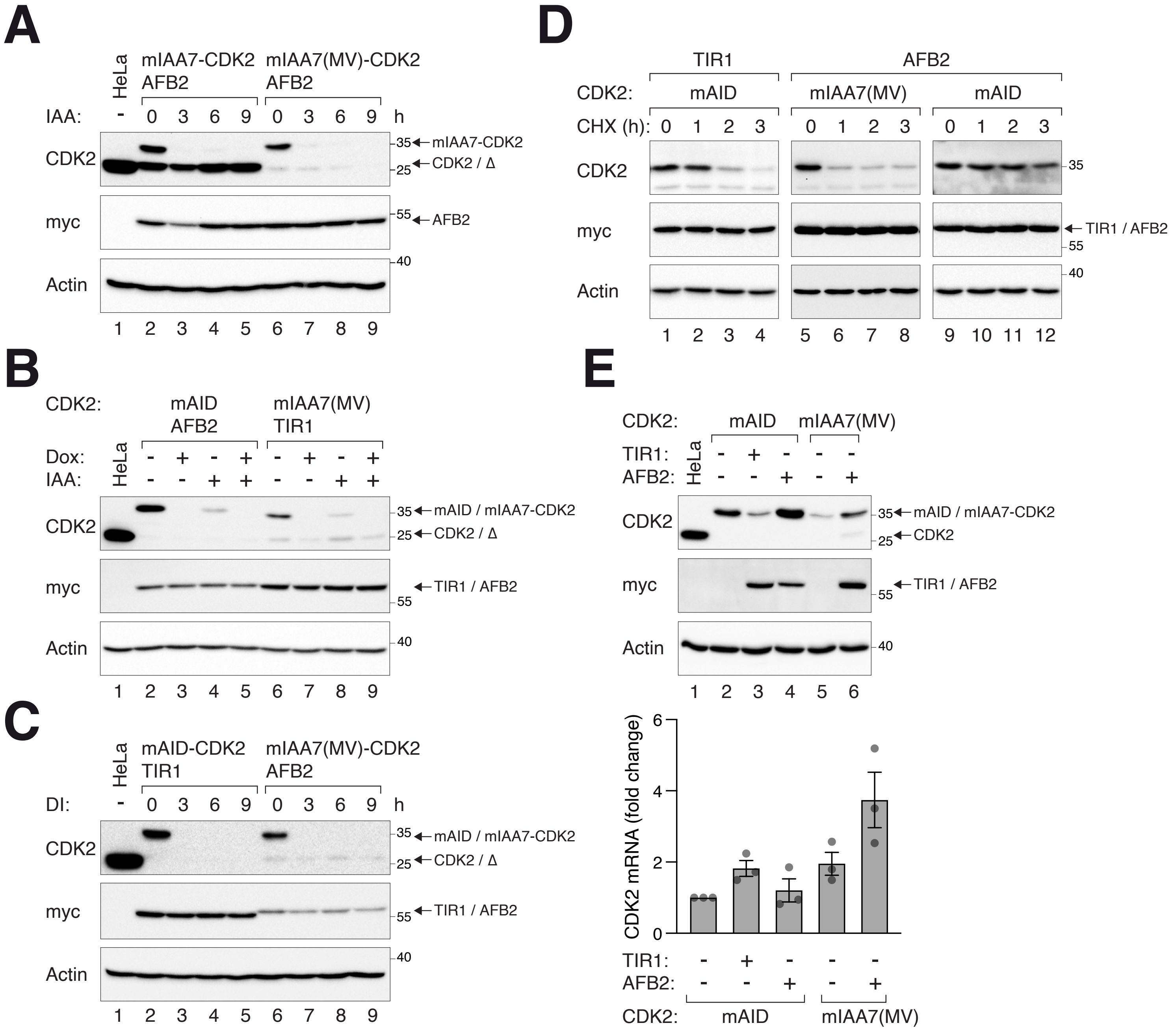

Journal of Cell Science • Supplementary information

Fig. S2. Mutation of Met^{52} largely abolishes the expression of truncated products from mIAA7.

(A) Mutation of Met^{52} largely abolishes the appearance of truncated products from mIAA7. Point mutation M52V was introduced into mIAA7 to generate mIAA7(MV). Cell lines expressing mIAA7- or mIAA7(MV)-tagged CDK2 in combination with AFB2 were generated. The cells were incubated with IAA and harvested at the indicated time points immunoblotting analysis. Equal loading of lysates was confirmed by immunoblotting for actin.

(B) Degrons (mAID and mIAA7) and F-box proteins (TIR1 and AFB2) are interchangeable. HeLa cells expressing ^{mAID}CDK2 and AFB2 or $^{mIAA7(MV)}$CDK2 with TIR1 were generated (endogenous CDK2 was also disrupted with CRISPR-Cas9). The cells were treated with Dox and IAA individually or together for 24 h before analyzed with immunoblotting.

(C) Trace amounts of truncated products is still present in M52V-mutated mIAA7. Cell lines expressing mAID- or mIAA7(MV)-tagged CDK2 in combination with TIR1 or AFB2, respectively, were generated. The cells were incubated with DI and harvested at the indicated time points for immunoblotting analysis.

(D) Relatively low background degradation for mAID-tagged proteins in the presence of AFB2. Cell lines expressing the indicated combination of ^{mAID}CDK2, $^{mIAA7(MV)}$CDK2, TIR1, and AFB2 were treated with cycloheximide (CHX) to abolish de novo protein synthesis. The cells were harvested at different time points and analyzed with immunoblotting.

(E) mIAA7 has a higher background degradation rate than mAID. Cell lines expressing different configurations of mAID- or mIAA7(MV)-tagged CDK2 and TIR1 or AFB2 were generated. Protein expression was analyzed using immunoblotting (upper panel). The relative expression of recombinant CDK2 mRNA was quantified using RT-PCR (lower panel; same primers were used to detect both mAID- and mIAA7(MV)-tagged CDK2 mRNAs). Mean ± SEM from three independent experiments.

Journal of Cell Science • Supplementary information

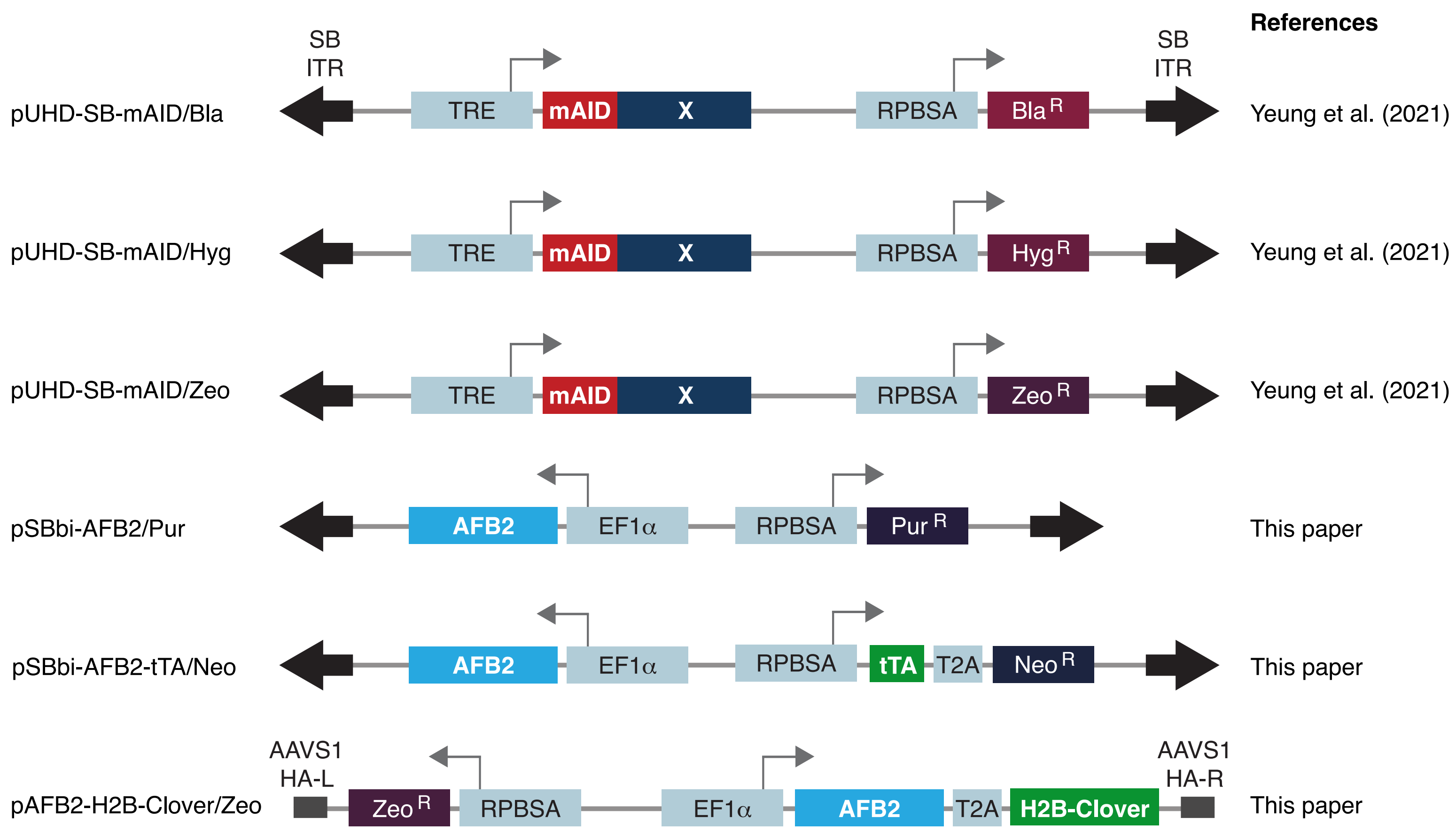

Fig. S3. Constructs for generating conditional gene silencing cell lines using the tTA–AID dual transcription–degron system.

The pUHD-SB-mAID series were as described in Yeung et al. (2021), containing TRE-driven mAID-fusion cDNA (X) placed in between the ITRs of SB transposon. The pSBbi-AFB2/Pur vector contains AFB2 driven by a constitutive EF1α promoter (also for SB transposon). The pSBbi-AFB2-tTA/Neo vector contains AFB2 driven by a constitutive EF1α promoter and tTA driven by a RPBSA promoter. pAFB2-H2B-Clover/Zeo expresses both AFB2 and histone H2B-Clover placed in between the 5' homology arm (HA-L) and 3' homology arm (HA-R) of AAVS1. Antibiotic resistance genes for blasticidin (Bla^R), hygromycin (Hyg^R), puromycin (Pur^R), zeocin (Zeo^R), or neomycin (Neo^R) are driven by a RPBSA promoter. T2A: T2A ribosomal skipping site.

Journal of Cell Science • Supplementary information

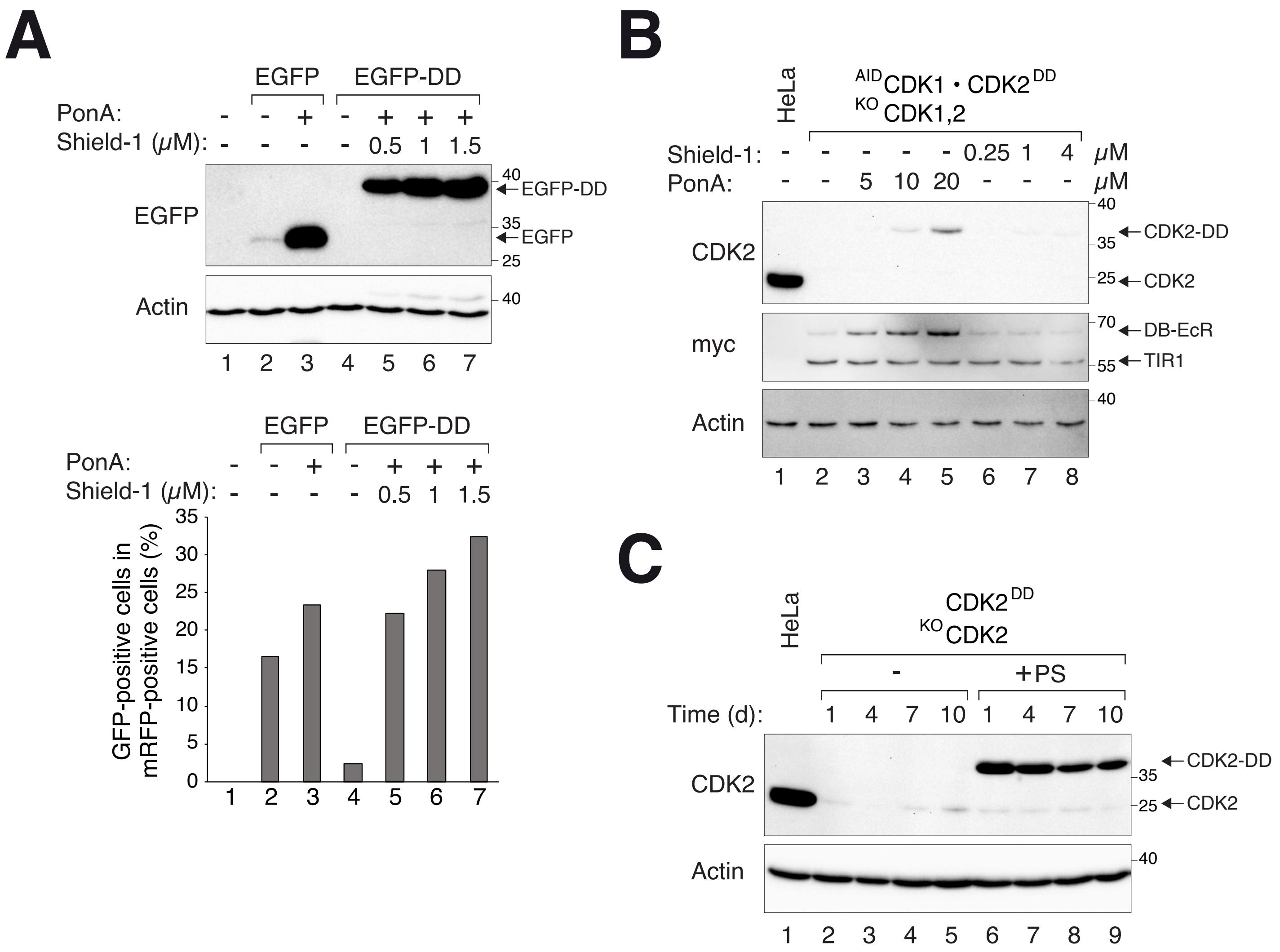

Fig. S4. Tight regulation of the DBEcR–DD dual transcription–degron system.

(A) DD-tag ensures low background expression. EGFP and EGFP^{DD} were put under the control of DBEcR (plasmid 25 and plasmid 31) and transfected into HeLa cells (an mRuby2-expressing plasmid was co-transfected in all samples, plasmid 44). The cells were treated with PonA and the indicated concentrations of Shield-1. After 24 h, the cells were harvested and analyzed with immunoblotting (upper panel). Note the expression of EGFP but not EGFP-DD in the absence of PonA or Shield-1 (lanes 2 and 4). The cells were also harvested for flow cytometry analysis (lower panel). The percentage of cells expressing GFP in mRuby2-expressing cells is shown.

(B) PonA or Shield-1 alone only weakly induces gene activation. CDK2^{DD}-expressing cells (in an ^{AID}CDK1, CDK1- and CDK2-KO background) were incubated with different concentrations of PonA or Shield-1. The cells were harvested after 24 h and analyzed with immunoblotting.

(C) Sustained activation of DD-tagged proteins. CDK2^{DD}-expressing cells in a CDK2-KO background were either untreated or incubated with PS. Lysates were prepared on different days and analyzed with immunoblotting. Note that cells were plated initially at low density; and PS was not replenished during the experiment.

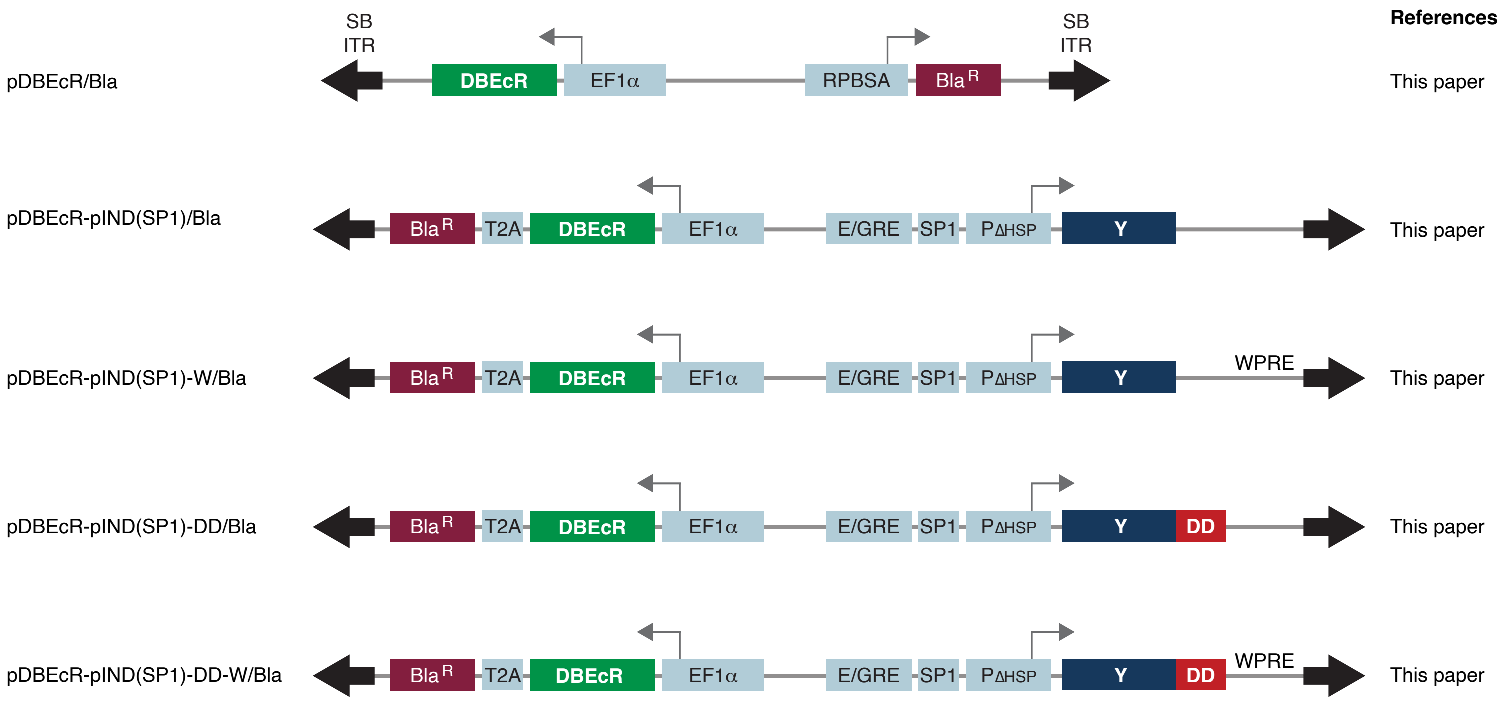

Fig. S5. Constructs for generating conditional gene activating cell lines using the DBEcR–DD dual transcription–degron system.
The pDBEcR series of vectors contain pDBEcR (myc-tagged) driven by a constitutive EF1**α** promoter and placed in between the ITRs of SB transposon. The pDBEcR-pIND(SP1) series further include the gene of interest (Y) under the control of a promoter containing E/GRE (ecdysone/glucocorticoid response element x6), SP1
(Specificity Protein 1 enhancer x3), and $P_{\Delta HSP}$ (minimal heat shock protein promoter). Some versions of the vectors contain DD degron (mutated FKBP12-derived destabilization domain degron) fused to the C-terminus of "Y". All available vectors contain EGFP as "Y", which can be excised to replace with a gene of interest. Blasticidin (Bla^R) resistance gene is driven either by a RPBSA promoter or T2A ribosomal skipping site. Some versions of the vectors contain WPRE at the 3'-UTR of "Y". Sequence information of pDBEcR-pIND(SP1)/Bla, pDBEcR-pIND(SP1)-W/Bla, pDBEcR-pIND(SP1)-DD/Bla, and pDBEcR-pIND(SP1)-DD/Bla are shown below.

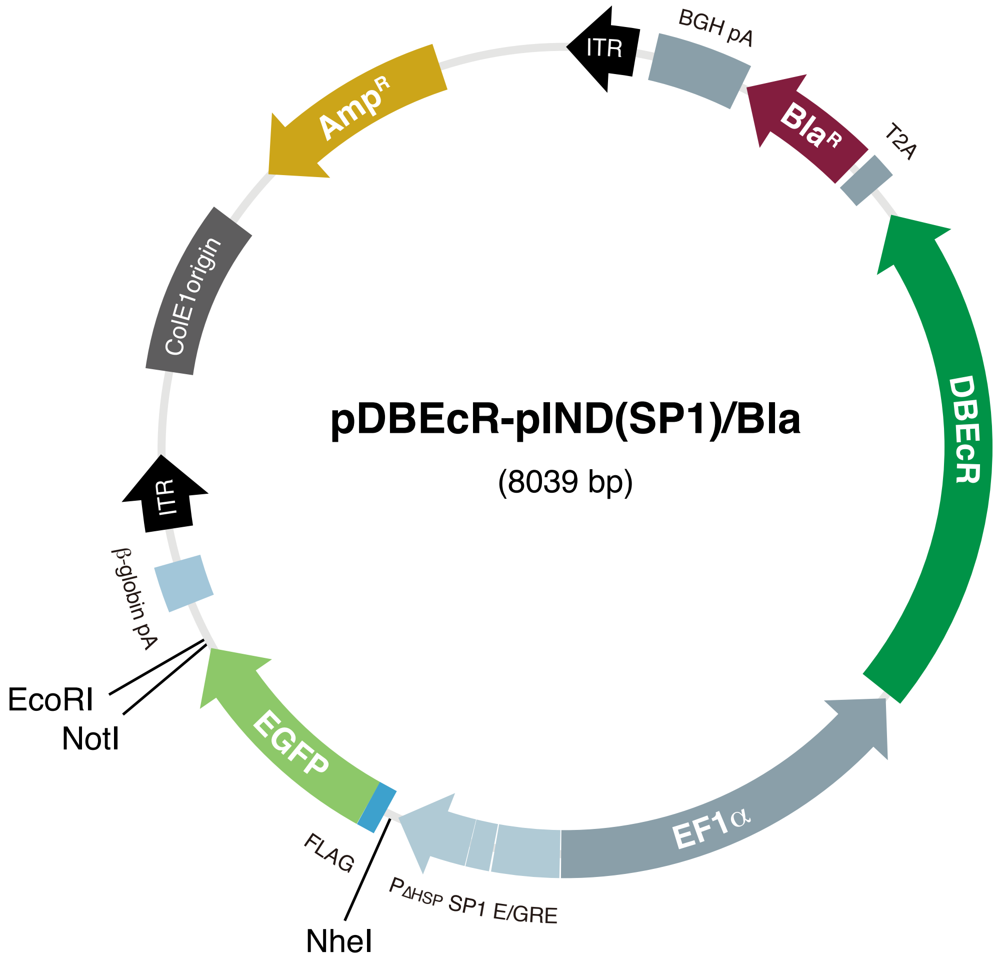

Feature	Location (bases)
Sleeping Beauty 5′ inverted terminal repeat	1–227
BGH polyadenylation signal	283–558
Blasticidin resistance gene	595–993
T2A ribosomal skipping site	997–1059
DBEcR	1207–2799
EF1**α** promoter	2821–4013
E/GRE ecdysone/glucocorticoid response element x6	4058–4231
SP1 specificity Protein 1 enhancer x3	4265–4312
$P_{\Delta HSP}$ minimal heat shock protein promoter	4327–4565
FLAG-tagged EGFP	4618–5394
**β**-globin polyadenylation signal	5578–5632
Sleeping Beauty 3′ inverted terminal repeat	5783–6008
ColE1 origin	6196–6784
Ampicillin resistance gene	6958–7617

Journal of Cell Science • Supplementary information

pDBEcR-pIND(SP1)/Bla

Region containing FLAG (grey) and EGFP (green).
Unique restriction enzyme sites are indicated.

```
                 FLAG                                                       EGFP
  NheI        M  D  Y  K  D  D  D  D  K  A  M  L  E  V  L  F  Q  G  P  M  V  S  K  G  E  E
  GCTAGCCACAATGGACTACAAAGACGATGACGATAAAGCCATGCTGGAAGTTCTGTTTCAGGGGCCCATGGTGAGCAAGGGCGAGGAG

 L  F  T  G  V  V  P  I  L  V  E  L  D  G  D  V  N  G  H  K  F  S  V  S  G  E  G  E  G  D
CTGTTCACCGGGGTGGTGCCCATCCTGGTCGAGCTGGACGGCGACGTAAACGGCCACAAGTTCAGCGTGTCCGGCGAGGGCGAGGGCGAT

 A  T  Y  G  K  L  T  L  K  F  I  C  T  T  G  K  L  P  V  P  W  P  T  L  V  T  T  L  T  Y
GCCACCTACGGCAAGCTGACCCTGAAGTTCATCTGCACCACCGGCAAGCTGCCCGTGCCCTGGCCCACCCTCGTGACCACCCTGACCTAC

 G  V  Q  C  F  S  R  Y  P  D  H  M  K  Q  H  D  F  F  K  S  A  M  P  E  G  Y  V  Q  E  R
GGCGTGCAGTGCTTCAGCCGCTACCCCGACCACATGAAGCAGCACGACTTCTTCAAGTCCGCCATGCCCGAAGGCTACGTCCAGGAGCGC

 T  I  F  F  K  D  D  G  N  Y  K  T  R  A  E  V  K  F  E  G  D  T  L  V  N  R  I  E  L  K
ACCATCTTCTTCAAGGACGACGGCAACTACAAGACCCGCGCCGAGGTGAAGTTCGAGGGCGACACCCTGGTGAACCGCATCGAGCTGAAG

 G  I  D  F  K  E  D  G  N  I  L  G  H  K  L  E  Y  N  Y  N  S  H  N  V  Y  I  M  A  D  K
GGCATCGACTTCAAGGAGGACGGCAACATCCTGGGGCACAAGCTGGAGTACAACTACAACAGCCACAACGTCTATATCATGGCCGACAAG

 Q  K  N  G  I  K  V  N  F  K  I  R  H  N  I  E  D  G  S  V  Q  L  A  D  H  Y  Q  Q  N  T
CAGAAGAACGGCATCAAGGTGAACTTCAAGATCCGCCACAACATCGAGGACGGCAGCGTGCAGCTCGCCGACCACTACCAGCAGAACACC

 P  I  G  D  G  P  V  L  L  P  D  N  H  Y  L  S  T  Q  S  A  L  S  K  D  P  N  E  K  R  D
CCCATCGGCGACGGCCCCGTGCTGCTGCCCGACAACCACTACCTGAGCACCCAGTCCGCCCTGAGCAAAGACCCCAACGAGAAGCGCGAT

 H  M  V  L  L  E  F  V  T  A  A  G  I  T  L  G  M  D  E  L  Y  K  *  NotI
CACATGGTCCTGCTGGAGTTCGTGACCGCCGCCGGGATCACTCTCGGCATGGACGAGCTGTACAAGTAAAGCGGCCGCGACTCTAGAGGA
              EcoRI
TCCAGACATGATAAGAATTC
```

Journal of Cell Science • Supplementary information

pDBEcR-pIND(SP1)/Bla

CAGTTGAAGTCGGAAGTTTACATACACTTAAGTTGGAGTCATTAAAACTCGTTTTTCAACTACTCCACAAATTTC
TTGTTAACAAACAATAGTTTTGGCAAGTCAGTTAGGACATCTACTTTGTGCATGACACAAGTCATTTTTCCAACA
ATTGTTTACAGACAGATTATTTCACTTATAATTCACTGTATCACAATTCCAGTGGGTCAGAAGTTTACATACACT
AAGTTCGACTCCTCTGCAGAATGCGGCGATGTTTCGGTAAGGGGTCCGCTATCTAGTAGGCCCAGCTGGTTCTTT
CCGCCTCAGAAGCCATAGAGCCCACCGCATCCCCAGCATGCCTGCTATTGTCTTCCCAATCCTCCCCCTTGCTGT
CCTGCCCCACCCCACCCCCCAGAATAGAATGACACCTACTCAGACAATGCGATGCAATTTCCTCATTTTATTAGG
AAAGGACAGTGGGAGTGGCACCTTCCAGGGTCAAGGAAGGCACGGGGGAGGGGCAAACAACAGATGGCTGGCAAC
TAGAAGGCACAGTCGAGGCTGATCAGCGAGCTCTAGAGAATTGATCCCCAAGCTTCGGCCACGAAGTGCTTAGCC
CTCCCACACATAACCAGAGGGCAGCAATTCACGAATCCCAACTGCCGTCGGCTGTCCATCACTGTCCTTCACTAT
GGCTTTGATCCCAGGATGCAGATCGAGAAGCACCTGTCGGCACCGTCCGCAGGGGCTCAAGATGCCCCTGTTCTC
ATTTCCGATCGCGACGATACAAGTCAGGTTGCCAGCTGCCGCAGCAGCAGCAGTGCCCAGCACCACGAGTTCTGC
ACAAGGTCCCCCAGTAAAATGATATACATTGACACCAGTGAAGATGCGGCCGTCGCTAGAGAGAGCTGCGCTGGC
GACGCTGTAGTCTTCAGAGATGGGGATGCTGTTGATTGTAGCCGTTGCTCTTTCAATGAGGGTGGATTCTTCTTG
AGACAAAGGCTTGGCCATCGATGGGCCAGGATTCTCCTCGACGTCACCGCATGTTAGCAGACTTCCTCTGCCCTC
TCCACTACCAGACTTGCTAGTGGATCCGTTCAAGTCTTCTTCTGAGATTAATTTTTGTTCACCGTTCAAGTCTTC
CTCGGAGATTAGCTTTTGTTCACCGTTCAAATCTTCTTCAGAAATCAACTTTTGTTCACCACTTGCGGCAGAACC
GGATCCCAGCACCACGGGGTTGGTGGGGGGCAGCACGGTGGGGTGGGTGGTGGCCACCTCGGCCACGTCCCAGAT
CTCCTCCAGGAAGGGGGGCAGCTTGCGGTTCTTCAGCTTCAGGCTGATGCACATGTTGCTGTTCTGGGTGCCCAG
GGTGCGCAGCTCGGTCAGCACGCTCAGGATGCGGCCGTAGATCACGGCGCAGCGGCTGCTGGCGCTGTTCTGGTT
GATGATGTAGATGCGCAGGGTGTTCAGGTAGTAGCGCTGGATCTCCTCCACCAGGCTGGGCTGCTCCAGGCCGGG
GCGGTCGCTGAAGATCACGATGGCGGTCAGCAGGGCGAAGTGCACGTTGTCCATGCCCATCGCGAACATGCAGCG
GCAGAAGTGCAGCAGGTCCTCGATCACGTAGGCCATGCCGCCCTGGCGGTAGTTGTCGCGGGTGTAGGCCTTGTT
GTTGGCGAACAGCACGCTGTCGCTGGCGGCGTCGTAGCGGCGGGCCACGCGCAGCATCATCACCTCGCTGCTGCT
GGCCTTCAGCAGGGTGATCTGGTCGCTCTGGCTGATCTTGCTGAAGCCGGGCAGGCCCTTGGCGAACTCCACGAT
CAGCTGCACGGTCAGGATGGTCATCTCGGTGATCTGGCGGAAGGGCAGGTCGCTCTCCTCGTCCTCCTCGTCGCT
CTGCCAGGTCTGGGTCACGCGCTTCAGGTCCTCGTCGCTGGGCTGCTCGTAGCCCTCCTGGTACCACACCAGGCG
GGCGATCAGGCTCTTCTGGTTGGCGCTCAGGGGGGGGATGTTCTTCTGGCGGTTCTGCTCCATCAGCTTCTCGCT
CAGGTAGCGGGGCACCACCTCGTGGATGCGGGCGGCCTCGGGGGGGGGGGGGTCGCACTGCATGATGGGGGGCAT
GTGGTCCTCCACGGTGGTGGTGCTCACGGGCAGCAGGATGCCCTTGTCCTTCTTCTGGCGCTGGCGGTCCTTGTT
CTTGCTGGGCTCCTGTATGACGCATTCCGGCCGCATACCCACGGCCAGGCACTTTTTCAGGCGGCACTCCTGACA
CTTTCGCCTCATGTACATGTCCATTTCGCAGGCGCGCCCGAACTTGCAGCAGTAGACGGCGCTCTTCGTAACGCT
GCGTCGAAAGAACACCTTGCAGGATCCACAGGTGAGGGCGTTGTAGTGGTAGCCGGAGGCCCTGTCGCCGCAAAC
CAGGCACAGCTCCTCTTGCACCCGTGGCGCAGGTCCCTTCTTGCTCTTCTTCGCATCGCAGCTTTCGTTCGCCGA
GTATCCGTTCAAGCTGCTCGAAGGCGAGAGATCATCGCGACCTGAAGATATAGAATTTGATATTCTTCTAGAGGT
ACCTAGAAGCTTCCCACCGTACTCGTCAATTCCAAGGGCATCGGTAAACATCTGCTCAAACTCGAAGTCGGCCAT
ATCCAGAGCGCCGTAGGGGGCGGAGTCGTGGGGGGTAAATCCCGGACCTGGGGAATCCCCGTCCCCCAACATGTC
CAGATCGAAATCGTCTAGCGCGTCGGCATGCGCCATCGCCACGTCCTCGCCGTCTAAGTGGAGTTCGTCCCCCAG
GCTGACATCGGTCGGGGGGGCCATGGTGGCCTCAGAGGCCAGCTTGGGGTAGTTTTCACGACACCTTAAATGGAA
GAAAAAAACTTTGAACCACTGTCTGAGGCTTGAGAATGAACCAAGATCCAAACTCAAAAAGGGCAAATTCCAAGG
AGAATTACATCAAGTGCCAAGCTGGCCTAACTTCAGTCTCCACCCACTCAGTATGGGGAAACTCCATCGCATAAA
ACCCCTCCCCCCAACCTAAAGACGACGTACTCCAAAAGCTCGAGAACTAATCGAGGTGCCTGGACGGCGCCCGGT
ACTCCGTGGAGTCACATGAAGCGACGGCTGAGGACGGAAAGGCCCTTTTCCTTTGTGTGGGTGACTCACCCGCCC
GCTCTCCCGAGCGCCGCGTCCTCCATTTTGAGCTCCCTGCAGCAGGGCCGGGAAGCGGCCATCTTTCCGCTCACG
CAACTGGTGCCGACCGGGCCAGCCTTGCCGCCCAGGGCGGGGCGATACACGGCGGCGCGAGGCCAGGCACCAGAG
CAGGCCGGCCAGCTTGAGACTACCCCCGTCCGATTCTCGGTGGCCGCGCTCGCAGGCCCCGCCTCGCCGAACATG
TGCGCTGGGACGCACGGGCCCCGTCGCCGCCCGCGGCCCCAAAAACCGAAATACCAGTGTGCAGATCTTGGCCCG
CATTTACAAGACTATCTTGCCAGAAAAAAAGCGTCGCAGCAGGTCATCAAAAATTTTAAATGGCTAGAGACTTAT
CGAAAGCAGCGAGACAGGCGCGAAGGTGCCACCAGATTCGCACGCGGCGGCCCCAGCGCCCAGGCCAAGCCTCAA
CTCAAGCACGAGGCGAAGGGGCTCCTTAAGCGCAAGGCCTCGAACTCTCCCACCCACTTCCAACCCGAAGCTCGG
GATCAAGAATCACGTACTGCAGCCAGGGGCGTGGAAGTAATTCAAGGCACGCAAGGGCCATAACCCGTAAAGAGG
CCAGGCCCGCGGGAACCACACACGGCACTTACCTGTGTTCTGGCGGCAAACCCGTTGCGAAAAAGAACGTTCACG
GCGACTACTGCACTTATATACGGTTCTCCCCCACCCTCGGGAAAAAGGCGGAGCCAGTACACGACATCACTTTCC
CAGTTTACCCCGCGCCACCTTCTCTAGGCACCGGTTCAATTGCCGACCCCTCCCCCCAACTTCTCGGGGACTGTG
GGCGATGTGCGCTCTGCCCACTGACGGGCACCGGAGCCTCTAGGGTCGACCTCGACGGATCGGGAGATCTCGGCC
GCATATTAAGTGCATTGTTCTCGATACCGCTAAGTGCATTGTTCTCGTTAGCTCGATGGACAAGTGCATTGTTCT
CTTGCTGAAAGCTCGATGGACAAGTGCATTGTTCTCTTGCTGAAAGCTCGATGGACAAGTGCATTGTTCTCTTGC
TGAAAGCTCGATGGACAAGTGCATTGTTCTCTTGCTGAAAGCTCAGTACCCGGGTCGGAGTACTGCCCCGCCCCT
AGCGATTAGCCCCGGCCCCGCATAGCTCCGCCCCGGGAGTACCCTCGACCGCCGGAGTATAAATAGAGGCGCTTC
GTCTACGGAGCGACAATTCAATTCAAACAAGCAAAGTGAACACGTCGCTAAGCGAAAGCTAAGCAAATAAACAAG
CGCAGCTGAACAAGCTAAACAATCTGCAGTAAAGTGCAAGTTAAAGTGAATCAATTAAAAGTAACCAGCAACCAA

Journal of Cell Science • Supplementary information

GTAAATCAACTGCAACTACTGAAATCTGCCAAGAAGTAATTATTGAATACAAGAAGAGAACTCTGAATACTTTCA
ACAAGTTACCGAGAAAGAAGAACTCACACACAGCTAGCCACAATGGACTACAAAGACGATGACGATAAAGCCATG
CTGGAAGTTCTGTTTCAGGGGCCCATGGTGAGCAAGGGCGAGGAGCTGTTCACCGGGGTGGTGCCCATCCTGGTC
GAGCTGGACGGCGACGTAAACGGCCACAAGTTCAGCGTGTCCGGCGAGGGCGAGGGCGATGCCACCTACGGCAAG
CTGACCCTGAAGTTCATCTGCACCACCGGCAAGCTGCCCGTGCCCTGGCCCACCCTCGTGACCACCCTGACCTAC
GGCGTGCAGTGCTTCAGCCGCTACCCCGACCACATGAAGCAGCACGACTTCTTCAAGTCCGCCATGCCCGAAGGC
TACGTCCAGGAGCGCACCATCTTCTTCAAGGACGACGGCAACTACAAGACCCGCGCCGAGGTGAAGTTCGAGGGC
GACACCCTGGTGAACCGCATCGAGCTGAAGGGCATCGACTTCAAGGAGGACGGCAACATCCTGGGGCACAAGCTG
GAGTACAACTACAACAGCCACAACGTCTATATCATGGCCGACAAGCAGAAGAACGGCATCAAGGTGAACTTCAAG
ATCCGCCACAACATCGAGGACGGCAGCGTGCAGCTCGCCGACCACTACCAGCAGAACACCCCCATCGGCGACGGC
CCCGTGCTGCTGCCCGACAACCACTACCTGAGCACCCAGTCCGCCCTGAGCAAAGACCCCAACGAGAAGCGCGAT
CACATGGTCCTGCTGGAGTTCGTGACCGCCGCCGGGATCACTCTCGGCATGGACGAGCTGTACAAGTAAAGCGGC
CGCGACTCTAGAGGATCCAGACATGATAAGAATTCACTCCTCAGGTGCAGGCTGCCTATCAGAAGGTGGTGGCTG
GTGTGGCCAATGCCCTGGCTCACAAATACCACTGAGATCTTTTTCCCTCTGCCAAAAATTATGGGGACATCATGA
AGCCCCTTGAGCATCTGACTTCTGGCTAATAAAGGAAATTTATTTTCATTGCAATAGTGTGTTGGAATTTTTTGT
GTCTCTCACTCGGAAGGACATATGGGAGGGCAAATCATTTAAAACATCAGAATGAGTATTTGGTTTAGAGTTTGG
CAACATATGCCATATGCTGGCTGCCATGAACTAGCTACTCGGGACCCCTTACCGAAACATCGCCGCATTCTGCAG
AGGAGTCGAGTGTATGTAAACTTCTGACCCACTGGGAATGTGATGAAAGAAATAAAAGCTGAAATGAATCATTCT
CTCTACTATTATTCTGATATTTCACATTCTTAAAATAAAGTGGTGATCCTAACTGACCTAAGACAGGGAATTTTT
ACTAGGATTAAATGTCAGGAATTGTGAAAAAGTGAGTTTAAATGTATTTGGCTAAGGTGTATGTAAACTTCCGAC
TTCAACTGTATAGGGATCCGCTTCCTCGCTCACTGACTCGCTGCGCTCGGTCGTTCGGCTGCGGCGAGCGGTATC
AGCTCACTCAAAGGCGGTAATACGGTTATCCACAGAATCAGGGGATAACGCAGGAAAGAACATGTGAGCAAAAGG
CCAGCAAAAGGCCAGGAACCGTAAAAAGGCCGCGTTGCTGGCGTTTTTCCATAGGCTCCGCCCCCCTGACGAGCA
TCACAAAAATCGACGCTCAAGTCAGAGGTGGCGAAACCCGACAGGACTATAAAGATACCAGGCGTTTCCCCCTGG
AAGCTCCCTCGTGCGCTCTCCTGTTCCGACCCTGCCGCTTACCGGATACCTGTCCGCCTTTCTCCCTTCGGGAAG
CGTGGCGCTTTCTCATAGCTCACGCTGTAGGTATCTCAGTTCGGTGTAGGTCGTTCGCTCCAAGCTGGGCTGTGT
GCACGAACCCCCCGTTCAGCCCGACCGCTGCGCCTTATCCGGTAACTATCGTCTTGAGTCCAACCCGGTAAGACA
CGACTTATCGCCACTGGCAGCAGCCACTGGTAACAGGATTAGCAGAGCGAGGTATGTAGGCGGTGCTACAGAGTT
CTTGAAGTGGTGGCCTAACTACGGCTACACTAGAAGAACAGTATTTGGTATCTGCGCTCTGCTGAAGCCAGTTAC
CTTCGGAAAAAGAGTTGGTAGCTCTTGATCCGGCAAACAAACCACCGCTGGTAGCGGTGGTTTTTTTGTTTGCAA
GCAGCAGATTACGCGCAGAAAAAAAGGATCTCAAGAAGATCCTTTGATCTTTTCTACGGGGTCTGACGCTCAGTG
GAACGAAAACTCACGTTAAGGGATTTTGGTCATGAGATTATCAAAAAGGATCTTCACCTAGATCCTTTTAAATTA
AAAATGAAGTTTTAAATCAATCTAAAGTATATATGAGTAAACTTGGTCTGACAGTTACCAATGCTTAATCAGTGA
GGCACCTATCTCAGCGATCTGTCTATTTCGTTCATCCATAGTTGCCTGACTCCCCGTCGTGTAGATAACTACGAT
ACGGGAGGGCTTACCATCTGGCCCCAGTGCTGCAATGATACCGCGAGACCCACGCTCACCGGCTCCAGATTTATC
AGCAATAAACCAGCCAGCCGGAAGGGCCGAGCGCAGAAGTGGTCCTGCAACTTTATCCGCCTCCATCCAGTCTAT
TAATTGTTGCCGGGAAGCTAGAGTAAGTAGTTCGCCAGTTAATAGTTTGCGCAACGTTGTTGCCATTGCTACAGG
CATCGTGGTGTCACGCTCGTCGTTTGGTATGGCTTCATTCAGCTCCGGTTCCCAACGATCAAGGCGAGTTACATG
ATCCCCCATGTTGTGCAAAAAAGCGGTTAGCTCCTTCGGTCCTCCGATCGTTGTCAGAAGTAAGTTGGCCGCAGT
GTTATCACTCATGGTTATGGCAGCACTGCATAATTCTCTTACTGTCATGCCATCCGTAAGATGCTTTTCTGTGAC
TGGTGAGTACTCAACCAAGTCATTCTGAGAATAGTGTATGCGGCGACCGAGTTGCTCTTGCCCGGCGTCAATACG
GGATAATACCGCGCCACATAGCAGAACTTTAAAAGTGCTCATCATTGGAAAACGTTCTTCGGGGCGAAAACTCTC
AAGGATCTTACCGCTGTTGAGATCCAGTTCGATGTAACCCACTCGTGCACCCAACTGATCTTCAGCATCTTTTAC
TTTCACCAGCGTTTCTGGGTGAGCAAAAACAGGAAGGCAAAATGCCGCAAAAAAGGGAATAAGGGCGACACGGAA
ATGTTGAATACTCATACTCTTCCTTTTTCAATATTATTGAAGCATTTATCAGGGTTATTGTCTCATGAGCGGATA
CATATTTGAATGTATTTAGAAAAATAAACAAATAGGGGTTCCGCGCACATTTCCCCGAAAAGTGCCACCTGATGC
GGTGTGAAATACCGCACAGATGCGTAAGGAGAAAATACCGCATCAGGAAATTGTAAGCGTTAATATTTTGTTAAA
ATTGGATCCCTATA

Fig. S5 cont.

pDBEcR-pIND(SP1)/Bla.

Graphic map, cloning sites, locations of different elements, and complete DNA sequence. The vector expresses FLAG-tagged EGFP under the control of a modified ecdysone promoter. DBEcR (myc-tagged at C-terminus) and blasticidin resistance gene are expressed from a constitutive EF1α promoter. FLAG-EGFP can be excised with NheI and NotI/EcoRI and replaced with other cDNAs.

Journal of Cell Science • Supplementary information

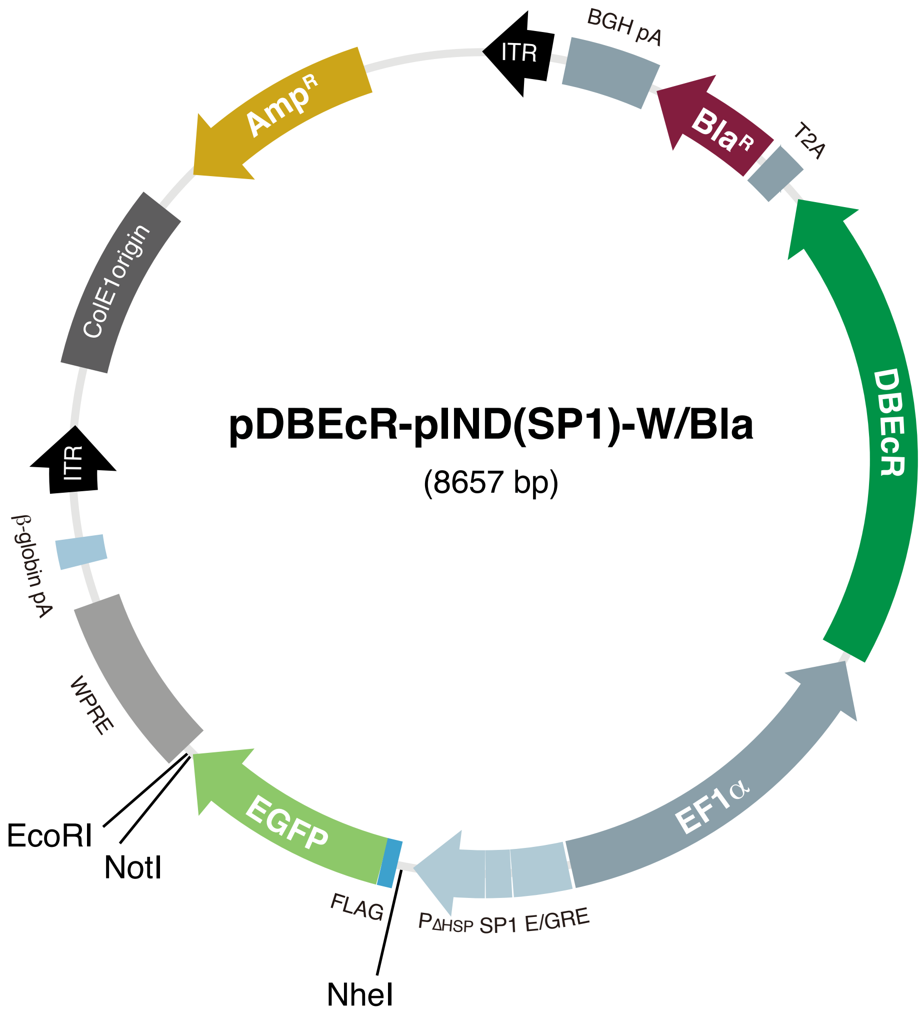

Feature	Location (bases)
Sleeping Beauty 5′ inverted terminal repeat	1–227
BGH polyadenylation signal	283–558
Blasticidin resistance gene	595–993
T2A ribosomal skipping site	997–1059
DBEcR	1207–2799
EF1**α** promoter	2821–4013
E/GRE ecdysone/glucocorticoid response element x6	4058–4231
SP1 specificity Protein 1 enhancer x3	4265–4312
$P_{\Delta HSP}$ minimal heat shock protein promoter	4327–4565
FLAG-tagged EGFP	4618–5394
WPRE	5446–6034
**β**-globin polyadenylation signal	6196–6250
Sleeping Beauty 3′ inverted terminal repeat	6401–6626
ColE1 origin	6814–7402
Ampicillin resistance gene	7576–8235

pDBEcR-pIND(SP1)-W/Bla

Region containing FLAG (grey) and EGFP (green).

Unique restriction enzyme sites are indicated.

```
                   FLAG                                                           EGFP
 NheI        M  D  Y  K  D  D  D  D  K  A  M  L  E  V  L  F  Q  G  P  M  V  S  K  G  E  E
 GCTAGCCACAATGGACTACAAAGACGATGACGATAAAGCCATGCTGGAAGTTCTGTTTCAGGGGCCCATGGTGAGCAAGGGCGAGGAG

 L  F  T  G  V  V  P  I  L  V  E  L  D  G  D  V  N  G  H  K  F  S  V  S  G  E  G  E  G  D
CTGTTCACCGGGGTGGTGCCCATCCTGGTCGAGCTGGACGGCGACGTAAACGGCCACAAGTTCAGCGTGTCCGGCGAGGGCGAGGGCGAT

 A  T  Y  G  K  L  T  L  K  F  I  C  T  T  G  K  L  P  V  P  W  P  T  L  V  T  T  L  T  Y
GCCACCTACGGCAAGCTGACCCTGAAGTTCATCTGCACCACCGGCAAGCTGCCCGTGCCCTGGCCCACCCTCGTGACCACCCTGACCTAC

 G  V  Q  C  F  S  R  Y  P  D  H  M  K  Q  H  D  F  F  K  S  A  M  P  E  G  Y  V  Q  E  R
GGCGTGCAGTGCTTCAGCCGCTACCCCGACCACATGAAGCAGCACGACTTCTTCAAGTCCGCCATGCCCGAAGGCTACGTCCAGGAGCGC

 T  I  F  F  K  D  D  G  N  Y  K  T  R  A  E  V  K  F  E  G  D  T  L  V  N  R  I  E  L  K
ACCATCTTCTTCAAGGACGACGGCAACTACAAGACCCGCGCCGAGGTGAAGTTCGAGGGCGACACCCTGGTGAACCGCATCGAGCTGAAG

 G  I  D  F  K  E  D  G  N  I  L  G  H  K  L  E  Y  N  Y  N  S  H  N  V  Y  I  M  A  D  K
GGCATCGACTTCAAGGAGGACGGCAACATCCTGGGGCACAAGCTGGAGTACAACTACAACAGCCACAACGTCTATATCATGGCCGACAAG

 Q  K  N  G  I  K  V  N  F  K  I  R  H  N  I  E  D  G  S  V  Q  L  A  D  H  Y  Q  Q  N  T
CAGAAGAACGGCATCAAGGTGAACTTCAAGATCCGCCACAACATCGAGGACGGCAGCGTGCAGCTCGCCGACCACTACCAGCAGAACACC

 P  I  G  D  G  P  V  L  L  P  D  N  H  Y  L  S  T  Q  S  A  L  S  K  D  P  N  E  K  R  D
CCCATCGGCGACGGCCCCGTGCTGCTGCCCGACAACCACTACCTGAGCACCCAGTCCGCCCTGAGCAAAGACCCCAACGAGAAGCGCGAT

 H  M  V  L  L  E  F  V  T  A  A  G  I  T  L  G  M  D  E  L  Y  K  *  NotI
CACATGGTCCTGCTGGAGTTCGTGACCGCCGCCGGGATCACTCTCGGCATGGACGAGCTGTACAAGTAAAGCGGCCGCGACTCTAGAGGA
              EcoRI
 S  R  H  D  K  N  S  L  I  G
TCCAGACATGATAAGAATTCTCTTATCGGT
```

Journal of Cell Science • Supplementary information

pDBEcR-pIND(SP1)-W/Bla

CAGTTGAAGTCGGAAGTTTACATACACTTAAGTTGGAGTCATTAAAACTCGTTTTTCAACTACTCCACAAATTTC
TTGTTAACAAACAATAGTTTTGGCAAGTCAGTTAGGACATCTACTTTGTGCATGACACAAGTCATTTTTCCAACA
ATTGTTTACAGACAGATTATTTCACTTATAATTCACTGTATCACAATTCCAGTGGGTCAGAAGTTTACATACACT
AAGTTCGACTCCTCTGCAGAATGCGGCGATGTTTCGGTAAGGGGTCCGCTATCTAGTAGGCCCAGCTGGTTCTTT
CCGCCTCAGAAGCCATAGAGCCCACCGCATCCCCAGCATGCCTGCTATTGTCTTCCCAATCCTCCCCCTTGCTGT
CCTGCCCCACCCCACCCCCCAGAATAGAATGACACCTACTCAGACAATGCGATGCAATTTCCTCATTTTATTAGG
AAAGGACAGTGGGAGTGGCACCTTCCAGGGTCAAGGAAGGCACGGGGGAGGGGCAAACAACAGATGGCTGGCAAC
TAGAAGGCACAGTCGAGGCTGATCAGCGAGCTCTAGAGAATTGATCCCCAAGCTTCGGCCACGAAGTGCTTAGCC
CTCCCACACATAACCAGAGGGCAGCAATTCACGAATCCCAACTGCCGTCGGCTGTCCATCACTGTCCTTCACTAT
GGCTTTGATCCCAGGATGCAGATCGAGAAGCACCTGTCGGCACCGTCCGCAGGGGCTCAAGATGCCCCTGTTCTC
ATTTCCGATCGCGACGATACAAGTCAGGTTGCCAGCTGCCGCAGCAGCAGCAGTGCCCAGCACCACGAGTTCTGC
ACAAGGTCCCCCAGTAAAATGATATACATTGACACCAGTGAAGATGCGGCCGTCGCTAGAGAGAGCTGCGCTGGC
GACGCTGTAGTCTTCAGAGATGGGGATGCTGTTGATTGTAGCCGTTGCTCTTTCAATGAGGGTGGATTCTTCTTG
AGACAAAGGCTTGGCCATCGATGGGCCAGGATTCTCCTCGACGTCACCGCATGTTAGCAGACTTCCTCTGCCCTC
TCCACTACCAGACTTGCTAGTGGATCCGTTCAAGTCTTCTTCTGAGATTAATTTTTGTTCACCGTTCAAGTCTTC
CTCGGAGATTAGCTTTTGTTCACCGTTCAAATCTTCTTCAGAAATCAACTTTTGTTCACCACTTGCGGCAGAACC
GGATCCCAGCACCACGGGGTTGGTGGGGGGCAGCACGGTGGGGTGGGTGGTGGCCACCTCGGCCACGTCCCAGAT
CTCCTCCAGGAAGGGGGGCAGCTTGCGGTTCTTCAGCTTCAGGCTGATGCACATGTTGCTGTTCTGGGTGCCCAG
GGTGCGCAGCTCGGTCAGCACGCTCAGGATGCGGCCGTAGATCACGGCGCAGCGGCTGCTGGCGCTGTTCTGGTT
GATGATGTAGATGCGCAGGGTGTTCAGGTAGTAGCGCTGGATCTCCTCCACCAGGCTGGGCTGCTCCAGGCCGGG
GCGGTCGCTGAAGATCACGATGGCGGTCAGCAGGGCGAAGTGCACGTTGTCCATGCCCATCGCGAACATGCAGCG
GCAGAAGTGCAGCAGGTCCTCGATCACGTAGGCCATGCCGCCCTGGCGGTAGTTGTCGCGGGTGTAGGCCTTGTT
GTTGGCGAACAGCACGCTGTCGCTGGCGGCGTCGTAGCGGCGGGCCACGCGCAGCATCATCACCTCGCTGCTGCT
GGCCTTCAGCAGGGTGATCTGGTCGCTCTGGCTGATCTTGCTGAAGCCGGGCAGGCCCTTGGCGAACTCCACGAT
CAGCTGCACGGTCAGGATGGTCATCTCGGTGATCTGGCGGAAGGGCAGGTCGCTCTCCTCGTCCTCCTCGTCGCT
CTGCCAGGTCTGGGTCACGCGCTTCAGGTCCTCGTCGCTGGGCTGCTCGTAGCCCTCCTGGTACCACACCAGGCG
GGCGATCAGGCTCTTCTGGTTGGCGCTCAGGGGGGGGATGTTCTTCTGGCGGTTCTGCTCCATCAGCTTCTCGCT
CAGGTAGCGGGGCACCACCTCGTGGATGCGGGCGGCCTCGGGGGGGGGGGGTCGCACTGCATGATGGGGGGCAT
GTGGTCCTCCACGGTGGTGGTGCTCACGGGCAGCAGGATGCCCTTGTCCTTCTTCTGGCGCTGGCGGTCCTTGTT
CTTGCTGGGCTCCTGTATGACGCATTCCGGCCGCATACCCACGGCCAGGCACTTTTTCAGGCGGCACTCCTGACA
CTTTCGCCTCATGTACATGTCCATTTCGCAGGCGCGCCCGAACTTGCAGCAGTAGACGGCGCTCTTCGTAACGCT
GCGTCGAAAGAACACCTTGCAGGATCCACAGGTGAGGGCGTTGTAGTGGTAGCCGGAGGCCCTGTCGCCGCAAAC
CAGGCACAGCTCCTCTTGCACCCGTGGCGCAGGTCCCTTCTTGCTCTTCTTCGCATCGCAGCTTTCGTTCGCCGA
GTATCCGTTCAAGCTGCTCGAAGGCGAGAGATCATCGCGACCTGAAGATATAGAATTTGATATTCTTCTAGAGGT
ACCTAGAAGCTTCCCACCGTACTCGTCAATTCCAAGGGCATCGGTAAACATCTGCTCAAACTCGAAGTCGGCCAT
ATCCAGAGCGCCGTAGGGGGCGGAGTCGTGGGGGGTAAATCCCGGACCTGGGGAATCCCCGTCCCCCAACATGTC
CAGATCGAAATCGTCTAGCGCGTCGGCATGCGCCATCGCCACGTCCTCGCCGTCTAAGTGGAGTTCGTCCCCCAG
GCTGACATCGGTCGGGGGGGCCATGGTGGCCTCAGAGGCCAGCTTGGGGTAGTTTTCACGACACCTTAAATGGAA
GAAAAAAACTTTGAACCACTGTCTGAGGCTTGAGAATGAACCAAGATCCAAACTCAAAAAGGGCAAATTCCAAGG
AGAATTACATCAAGTGCCAAGCTGGCCTAACTTCAGTCTCCACCCACTCAGTATGGGGAAACTCCATCGCATAAA
ACCCCTCCCCCCAACCTAAAGACGACGTACTCCAAAAGCTCGAGAACTAATCGAGGTGCCTGGACGGCGCCCGGT
ACTCCGTGGAGTCACATGAAGCGACGGCTGAGGACGGAAAGGCCCTTTTCCTTTGTGTGGGTGACTCACCCGCCC
GCTCTCCCGAGCGCCGCGTCCTCCATTTTGAGCTCCCTGCAGCAGGGCCGGGAAGCGGCCATCTTTCCGCTCACG
CAACTGGTGCCGACCGGGCCAGCCTTGCCGCCCAGGGCGGGGCGATACACGGCGGCGCGAGGCCAGGCACCAGAG
CAGGCCGGCCAGCTTGAGACTACCCCCGTCCGATTCTCGGTGGCCGCGCTCGCAGGCCCCGCCTCGCCGAACATG
TGCGCTGGGACGCACGGGCCCCGTCGCCGCCCGCGGCCCCAAAAACCGAAATACCAGTGTGCAGATCTTGGCCCG
CATTTACAAGACTATCTTGCCAGAAAAAAAGCGTCGCAGCAGGTCATCAAAAATTTTAAATGGCTAGAGACTTAT
CGAAAGCAGCGAGACAGGCGCGAAGGTGCCACCAGATTCGCACGCGGCGGCCCCAGCGCCCAGGCCAAGCCTCAA
CTCAAGCACGAGGCGAAGGGGCTCCTTAAGCGCAAGGCCTCGAACTCTCCCACCCACTTCCAACCCGAAGCTCGG
GATCAAGAATCACGTACTGCAGCCAGGGGCGTGGAAGTAATTCAAGGCACGCAAGGGCCATAACCCGTAAAGAGG
CCAGGCCCGCGGGAACCACACACGGCACTTACCTGTGTTCTGGCGGCAAACCCGTTGCGAAAAAGAACGTTCACG
GCGACTACTGCACTTATATACGGTTCTCCCCCACCCTCGGGAAAAAGGCGGAGCCAGTACACGACATCACTTTCC
CAGTTTACCCCGCGCCACCTTCTCTAGGCACCGGTTCAATTGCCGACCCCTCCCCCCAACTTCTCGGGGACTGTG
GGCGATGTGCGCTCTGCCCACTGACGGGCACCGGAGCCTCTAGGGTCGACCTCGACGGATCGGGAGATCTCGGCC
GCATATTAAGTGCATTGTTCTCGATACCGCTAAGTGCATTGTTCTCGTTAGCTCGATGGACAAGTGCATTGTTCT
CTTGCTGAAAGCTCGATGGACAAGTGCATTGTTCTCTTGCTGAAAGCTCGATGGACAAGTGCATTGTTCTCTTGC
TGAAAGCTCGATGGACAAGTGCATTGTTCTCTTGCTGAAAGCTCAGTACCCGGGTCGGAGTACTGCCCCGCCCCT
AGCGATTAGCCCCGGCCCCGCATAGCTCCGCCCCGGGAGTACCCTCGACCGCCGGAGTATAAATAGAGGCGCTTC
GTCTACGGAGCGACAATTCAATTCAAACAAGCAAAGTGAACACGTCGCTAAGCGAAAGCTAAGCAAATAAACAAG
CGCAGCTGAACAAGCTAAACAATCTGCAGTAAAGTGCAAGTTAAAGTGAATCAATTAAAAGTAACCAGCAACCAA

Journal of Cell Science • Supplementary information

GTAAATCAACTGCAACTACTGAAATCTGCCAAGAAGTAATTATTGAATACAAGAAGAGAACTCTGAATACTTTCA
ACAAGTTACCGAGAAAGAAGAACTCACACACAGCTAGCCACAATGGACTACAAAGACGATGACGATAAAGCCATG
CTGGAAGTTCTGTTTCAGGGGCCCATGGTGAGCAAGGGCGAGGAGCTGTTCACCGGGGTGGTGCCCATCCTGGTC
GAGCTGGACGGCGACGTAAACGGCCACAAGTTCAGCGTGTCCGGCGAGGGCGAGGGCGATGCCACCTACGGCAAG
CTGACCCTGAAGTTCATCTGCACCACCGGCAAGCTGCCCGTGCCCTGGCCCACCCTCGTGACCACCCTGACCTAC
GGCGTGCAGTGCTTCAGCCGCTACCCCGACCACATGAAGCAGCACGACTTCTTCAAGTCCGCCATGCCCGAAGGC
TACGTCCAGGAGCGCACCATCTTCTTCAAGGACGACGGCAACTACAAGACCCGCGCCGAGGTGAAGTTCGAGGGC
GACACCCTGGTGAACCGCATCGAGCTGAAGGGCATCGACTTCAAGGAGGACGGCAACATCCTGGGGCACAAGCTG
GAGTACAACTACAACAGCCACAACGTCTATATCATGGCCGACAAGCAGAAGAACGGCATCAAGGTGAACTTCAAG
ATCCGCCACAACATCGAGGACGGCAGCGTGCAGCTCGCCGACCACTACCAGCAGAACACCCCCATCGGCGACGGC
CCCGTGCTGCTGCCCGACAACCACTACCTGAGCACCCAGTCCGCCCTGAGCAAAGACCCCAACGAGAAGCGCGAT
CACATGGTCCTGCTGGAGTTCGTGACCGCCGCCGGGATCACTCTCGGCATGGACGAGCTGTACAAGTAAAGCGGC
CGCGACTCTAGAGGATCCAGACATGATAAGAATTCTCTTATCGGTAATCAACCTCTGGATTACAAAATTTGTGAA
AGATTGACTGGTATTCTTAACTATGTTGCTCCTTTTACGCTATGTGGATACGCTGCTTTAATGCCTTTGTATCAT
GCTATTGCTTCCCGTATGGCTTTCATTTTCTCCTCCTTGTATAAATCCTGGTTGCTGTCTCTTTATGAGGAGTTG
TGGCCCGTTGTCAGGCAACGTGGCGTGGTGTGCACTGTGTTTGCTGACGCAACCCCCACTGGTTGGGGCATTGCC
ACCACCTGTCAGCTCCTTTCCGGGACTTTCGCTTTCCCCCTCCCTATTGCCACGGCGGAACTCATCGCCGCCTGC
CTTGCCCGCTGCTGGACAGGGGCTCGGCTGTTGGGCACTGACAATTCCGTGGTGTTGTCGGGGAAATCATCGTCC
TTTCCTTGGCTGCTCGCCTGTGTTGCCACCTGGATTCTGCGCGGGACGTCCTTCTGCTACGTCCCTTCGGCCCTC
AATCCAGCGGACCTTCCTTCCCGCGGCCTGCTGCCGGCTCTGCGGCCTCTTCCGCGTCTTCGCCTTCGCCCTCAG
ACGAGTCGGATCTCCCTTTGGGCCGCCTCCCCGCATCGATACCGTCGCAATTCACTCCTCAGGTGCAGGCTGCCT
ATCAGAAGGTGGTGGCTGGTGTGGCCAATGCCCTGGCTCACAAATACCACTGAGATCTTTTTCCCTCTGCCAAAA
ATTATGGGGACATCATGAAGCCCCTTGAGCATCTGACTTCTGGCTAATAAAGGAAATTTATTTTCATTGCAATAG
TGTGTTGGAATTTTTTGTGTCTCTCACTCGGAAGGACATATGGGAGGGCAAATCATTTAAAACATCAGAATGAGT
ATTTGGTTTAGAGTTTGGCAACATATGCCATATGCTGGCTGCCATGAACTAGCTACTCGGGACCCCTTACCGAAA
CATCGCCGCATTCTGCAGAGGAGTCGAGTGTATGTAAACTTCTGACCCACTGGGAATGTGATGAAAGAAATAAAA
GCTGAAATGAATCATTCTCTCTACTATTATTCTGATATTTCACATTCTTAAAATAAAGTGGTGATCCTAACTGAC
CTAAGACAGGGAATTTTTACTAGGATTAAATGTCAGGAATTGTGAAAAAGTGAGTTTAAATGTATTTGGCTAAGG
TGTATGTAAACTTCCGACTTCAACTGTATAGGGATCCGCTTCCTCGCTCACTGACTCGCTGCGCTCGGTCGTTCG
GCTGCGGCGAGCGGTATCAGCTCACTCAAAGGCGGTAATACGGTTATCCACAGAATCAGGGGATAACGCAGGAAA
GAACATGTGAGCAAAAGGCCAGCAAAAGGCCAGGAACCGTAAAAAGGCCGCGTTGCTGGCGTTTTTCCATAGGCT
CCGCCCCCCTGACGAGCATCACAAAAATCGACGCTCAAGTCAGAGGTGGCGAAACCCGACAGGACTATAAAGATA
CCAGGCGTTTCCCCCTGGAAGCTCCCTCGTGCGCTCTCCTGTTCCGACCCTGCCGCTTACCGGATACCTGTCCGC
CTTTCTCCCTTCGGGAAGCGTGGCGCTTTCTCATAGCTCACGCTGTAGGTATCTCAGTTCGGTGTAGGTCGTTCG
CTCCAAGCTGGGCTGTGTGCACGAACCCCCCGTTCAGCCCGACCGCTGCGCCTTATCCGGTAACTATCGTCTTGA
GTCCAACCCGGTAAGACACGACTTATCGCCACTGGCAGCAGCCACTGGTAACAGGATTAGCAGAGCGAGGTATGT
AGGCGGTGCTACAGAGTTCTTGAAGTGGTGGCCTAACTACGGCTACACTAGAAGAACAGTATTTGGTATCTGCGC
TCTGCTGAAGCCAGTTACCTTCGGAAAAAGAGTTGGTAGCTCTTGATCCGGCAAACAAACCACCGCTGGTAGCGG
TGGTTTTTTTGTTTGCAAGCAGCAGATTACGCGCAGAAAAAAAGGATCTCAAGAAGATCCTTTGATCTTTTCTAC
GGGGTCTGACGCTCAGTGGAACGAAAACTCACGTTAAGGGATTTTGGTCATGAGATTATCAAAAAGGATCTTCAC
CTAGATCCTTTTAAATTAAAAATGAAGTTTTAAATCAATCTAAAGTATATATGAGTAAACTTGGTCTGACAGTTA
CCAATGCTTAATCAGTGAGGCACCTATCTCAGCGATCTGTCTATTTCGTTCATCCATAGTTGCCTGACTCCCCGT
CGTGTAGATAACTACGATACGGGAGGGCTTACCATCTGGCCCCAGTGCTGCAATGATACCGCGAGACCCACGCTC
ACCGGCTCCAGATTTATCAGCAATAAACCAGCCAGCCGGAAGGGCCGAGCGCAGAAGTGGTCCTGCAACTTTATC
CGCCTCCATCCAGTCTATTAATTGTTGCCGGGAAGCTAGAGTAAGTAGTTCGCCAGTTAATAGTTTGCGCAACGT
TGTTGCCATTGCTACAGGCATCGTGGTGTCACGCTCGTCGTTTGGTATGGCTTCATTCAGCTCCGGTTCCCAACG
ATCAAGGCGAGTTACATGATCCCCCATGTTGTGCAAAAAAGCGGTTAGCTCCTTCGGTCCTCCGATCGTTGTCAG
AAGTAAGTTGGCCGCAGTGTTATCACTCATGGTTATGGCAGCACTGCATAATTCTCTTACTGTCATGCCATCCGT
AAGATGCTTTTCTGTGACTGGTGAGTACTCAACCAAGTCATTCTGAGAATAGTGTATGCGGCGACCGAGTTGCTC
TTGCCCGGCGTCAATACGGGATAATACCGCGCCACATAGCAGAACTTTAAAAGTGCTCATCATTGGAAAACGTTC
TTCGGGGCGAAAACTCTCAAGGATCTTACCGCTGTTGAGATCCAGTTCGATGTAACCCACTCGTGCACCCAACTG
ATCTTCAGCATCTTTTACTTTCACCAGCGTTTCTGGGTGAGCAAAAACAGGAAGGCAAAATGCCGCAAAAAAGGG
AATAAGGGCGACACGGAAATGTTGAATACTCATACTCTTCCTTTTTCAATATTATTGAAGCATTTATCAGGGTTA
TTGTCTCATGAGCGGATACATATTTGAATGTATTTAGAAAAATAAACAAATAGGGGTTCCGCGCACATTTCCCCG
AAAAGTGCCACCTGATGCGGTGTGAAATACCGCACAGATGCGTAAGGAGAAAATACCGCATCAGGAAATTGTAAG
CGTTAATATTTTGTTAAAATTGGATCCCTATA

Fig. S5 cont.

pDBEcR-pIND(SP1)-W/Bla.

Graphic map, cloning sites, locations of different elements, and complete DNA sequence. The vector expresses FLAG-tagged EGFP under the control of a modified ecdysone promoter. An WPRE element is present in at the 3' UTR. DBEcR (myc-tagged at C-terminus) and blasticidin resistance gene are expressed from a constitutive EF1α promoter. FLAG-EGFP can be excised with NheI and NotI/EcoRI and replaced with other cDNAs.

Journal of Cell Science • Supplementary information

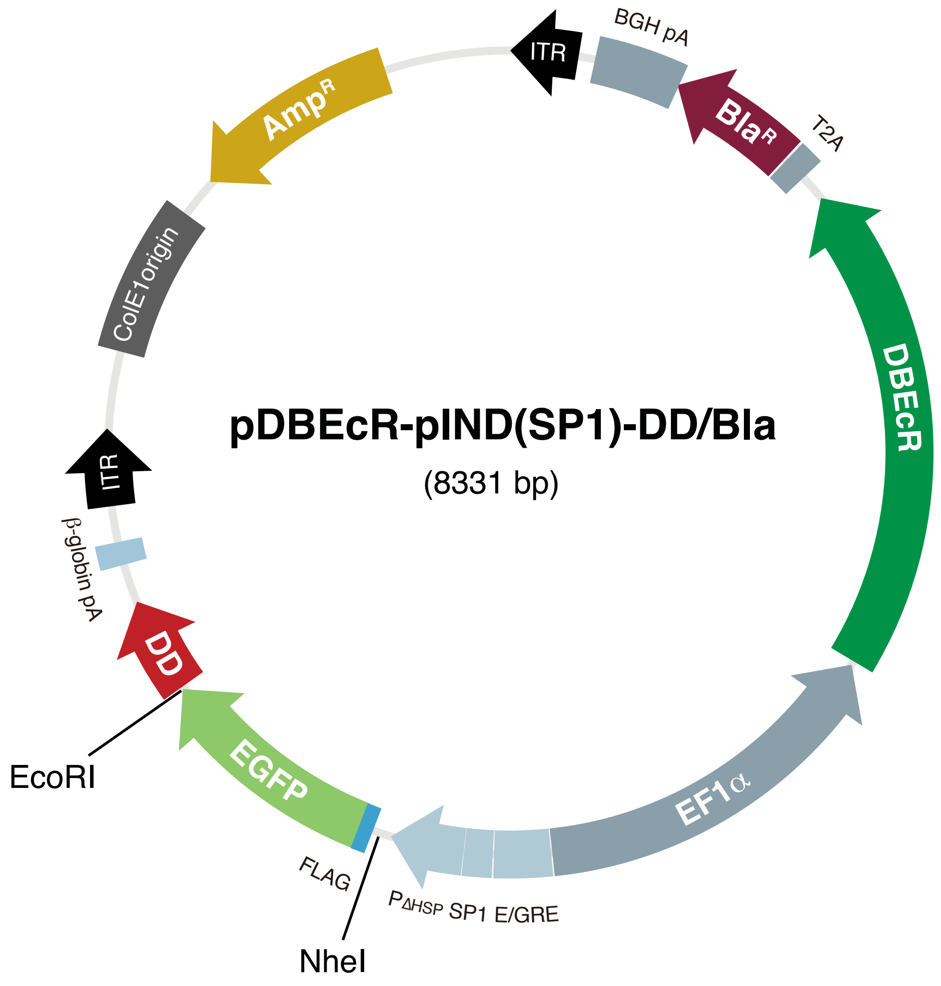

Feature	Location (bases)
Sleeping Beauty 5′ inverted terminal repeat	1–227
BGH polyadenylation signal	283–558
Blasticidin resistance gene	595–993
T2A ribosomal skipping site	997–1059
DBEcR	1207–2799
EF1**α** promoter	2821–4013
E/GRE ecdysone/glucocorticoid response element x6	4058–4231
SP1 specificity Protein 1 enhancer x3	4265–4312
$P_{\Delta HSP}$ minimal heat shock protein promoter	4327–4565
FLAG-tagged EGFP	4618–5391
DD-tag	5398–5724
**β**-globin polyadenylation signal	5870–5924
Sleeping Beauty 3′ inverted terminal repeat	6075–6300
ColE1 origin	6488–7076
Ampicillin resistance gene	7250–7909

pDBEcR-pIND(SP1)-DD/Bla

Region containing FLAG (grey), EGFP (green), and DD-tag (red).
Unique restriction enzyme sites are indicated.

```
 NheI          FLAG                                                EGFP
           M  D  Y  K  D  D  D  D  K  A  M  L  E  V  L  F  Q  G  P  M  V  S  K  G  E  E
 GCTAGCCACAATGGACTACAAAGACGATGACGATAAAGCCATGCTGGAAGTTCTGTTTCAGGGGCCCATGGTGAGCAAGGGCGAGGAG

 L  F  T  G  V  V  P  I  L  V  E  L  D  G  D  V  N  G  H  K  F  S  V  S  G  E  G  E  G  D
CTGTTCACCGGGGTGGTGCCCATCCTGGTCGAGCTGGACGGCGACGTAAACGGCCACAAGTTCAGCGTGTCCGGCGAGGGCGAGGGCGAT

 A  T  Y  G  K  L  T  L  K  F  I  C  T  T  G  K  L  P  V  P  W  P  T  L  V  T  T  L  T  Y
GCCACCTACGGCAAGCTGACCCTGAAGTTCATCTGCACCACCGGCAAGCTGCCCGTGCCCTGGCCCACCCTCGTGACCACCCTGACCTAC

 G  V  Q  C  F  S  R  Y  P  D  H  M  K  Q  H  D  F  F  K  S  A  M  P  E  G  Y  V  Q  E  R
GGCGTGCAGTGCTTCAGCCGCTACCCCGACCACATGAAGCAGCACGACTTCTTCAAGTCCGCCATGCCCGAAGGCTACGTCCAGGAGCGC

 T  I  F  F  K  D  D  G  N  Y  K  T  R  A  E  V  K  F  E  G  D  T  L  V  N  R  I  E  L  K
ACCATCTTCTTCAAGGACGACGGCAACTACAAGACCCGCGCCGAGGTGAAGTTCGAGGGCGACACCCTGGTGAACCGCATCGAGCTGAAG

 G  I  D  F  K  E  D  G  N  I  L  G  H  K  L  E  Y  N  Y  N  S  H  N  V  Y  I  M  A  D  K
GGCATCGACTTCAAGGAGGACGGCAACATCCTGGGGCACAAGCTGGAGTACAACTACAACAGCCACAACGTCTATATCATGGCCGACAAG

 Q  K  N  G  I  K  V  N  F  K  I  R  H  N  I  E  D  G  S  V  Q  L  A  D  H  Y  Q  Q  N  T
CAGAAGAACGGCATCAAGGTGAACTTCAAGATCCGCCACAACATCGAGGACGGCAGCGTGCAGCTCGCCGACCACTACCAGCAGAACACC

 P  I  G  D  G  P  V  L  L  P  D  N  H  Y  L  S  T  Q  S  A  L  S  K  D  P  N  E  K  R  D
CCCATCGGCGACGGCCCCGTGCTGCTGCCCGACAACCACTACCTGAGCACCCAGTCCGCCCTGAGCAAAGACCCCAACGAGAAGCGCGAT
                                                                      EcoRI DD
 H  M  V  L  L  E  F  V  T  A  A  G  I  T  L  G  M  D  E  L  Y  K  E  F  G  V  Q  V  E  T
CACATGGTCCTGCTGGAGTTCGTGACCGCCGCCGGGATCACTCTCGGCATGGACGAGCTGTACAAGGAATTCGGAGTGCAGGTGGAAACC

 I  S  P  G  D  G  R  T  F  P  K  R  G  Q  T  C  V  V  H  Y  T  G  M  L  E  D  G  K  K  V
ATCTCCCCAGGAGACGGGCGCACCTTCCCCAAGCGCGGCCAGACCTGTGTGGTGCACTACACCGGGATGCTTGAAGATGGAAAGAAAGTC

 D  S  S  R  D  R  N  K  P  F  K  F  M  L  G  K  Q  E  V  I  R  G  W  E  E  G  V  A  Q  M
GATTCCTCCCGGGACAGAAACAAGCCCTTTAAGTTTATGCTAGGCAAGCAGGAGGTGATCCGAGGCTGGGAAGAAGGGGTTGCCCAGATG

 S  V  G  Q  R  A  K  L  T  I  S  P  D  Y  A  Y  G  A  T  G  H  P  G  I  I  P  P  H  A  T
AGTGTGGGTCAGAGAGCCAAACTGACTATATCTCCAGATTATGCCTATGGTGCCACTGGGCACCCAGGCATCATCCCACCACATGCCACT

 L  V  F  D  V  E  L  L  K  P  E  A  *
CTCGTCTTCGATGTGGAGCTTCTAAAACCGGAAGCCTAG
```

Journal of Cell Science • Supplementary information

pDBEcR-pIND(SP1)-DD/Bla

CAGTTGAAGTCGGAAGTTTACATACACTTAAGTTGGAGTCATTAAAACTCGTTTTTCAACTACTCCACAAATTTC
TTGTTAACAAACAATAGTTTTGGCAAGTCAGTTAGGACATCTACTTTGTGCATGACACAAGTCATTTTTCCAACA
ATTGTTTACAGACAGATTATTTCACTTATAATTCACTGTATCACAATTCCAGTGGGTCAGAAGTTTACATACACT
AAGTTCGACTCCTCTGCAGAATGCGGCGATGTTTCGGTAAGGGGTCCGCTATCTAGTAGGCCCAGCTGGTTCTTT
CCGCCTCAGAAGCCATAGAGCCCACCGCATCCCCAGCATGCCTGCTATTGTCTTCCCAATCCTCCCCCTTGCTGT
CCTGCCCCACCCCACCCCCCAGAATAGAATGACACCTACTCAGACAATGCGATGCAATTTCCTCATTTTATTAGG
AAAGGACAGTGGGAGTGGCACCTTCCAGGGTCAAGGAAGGCACGGGGGAGGGGCAAACAACAGATGGCTGGCAAC
TAGAAGGCACAGTCGAGGCTGATCAGCGAGCTCTAGAGAATTGATCCCCAAGCTTCGGCCACGAAGTGCTTAGCC
CTCCCACACATAACCAGAGGGCAGCAATTCACGAATCCCAACTGCCGTCGGCTGTCCATCACTGTCCTTCACTAT
GGCTTTGATCCCAGGATGCAGATCGAGAAGCACCTGTCGGCACCGTCCGCAGGGGCTCAAGATGCCCCTGTTCTC
ATTTCCGATCGCGACGATACAAGTCAGGTTGCCAGCTGCCGCAGCAGCAGCAGTGCCCAGCACCACGAGTTCTGC
ACAAGGTCCCCCAGTAAAATGATATACATTGACACCAGTGAAGATGCGGCCGTCGCTAGAGAGAGCTGCGCTGGC
GACGCTGTAGTCTTCAGAGATGGGGATGCTGTTGATTGTAGCCGTTGCTCTTTCAATGAGGGTGGATTCTTCTTG
AGACAAAGGCTTGGCCATCGATGGGCCAGGATTCTCCTCGACGTCACCGCATGTTAGCAGACTTCCTCTGCCCTC
TCCACTACCAGACTTGCTAGTGGATCCGTTCAAGTCTTCTTCTGAGATTAATTTTTGTTCACCGTTCAAGTCTTC
CTCGGAGATTAGCTTTTGTTCACCGTTCAAATCTTCTTCAGAAATCAACTTTTGTTCACCACTTGCGGCAGAACC
GGATCCCAGCACCACGGGGTTGGTGGGGGGCAGCACGGTGGGGTGGGTGGTGGCCACCTCGGCCACGTCCCAGAT
CTCCTCCAGGAAGGGGGGCAGCTTGCGGTTCTTCAGCTTCAGGCTGATGCACATGTTGCTGTTCTGGGTGCCCAG
GGTGCGCAGCTCGGTCAGCACGCTCAGGATGCGGCCGTAGATCACGGCGCAGCGGCTGCTGGCGCTGTTCTGGTT
GATGATGTAGATGCGCAGGGTGTTCAGGTAGTAGCGCTGGATCTCCTCCACCAGGCTGGGCTGCTCCAGGCCGGG
GCGGTCGCTGAAGATCACGATGGCGGTCAGCAGGGCGAAGTGCACGTTGTCCATGCCCATCGCGAACATGCAGCG
GCAGAAGTGCAGCAGGTCCTCGATCACGTAGGCCATGCCGCCCTGGCGGTAGTTGTCGCGGGTGTAGGCCTTGTT
GTTGGCGAACAGCACGCTGTCGCTGGCGGCGTCGTAGCGGCGGGCCACGCGCAGCATCATCACCTCGCTGCTGCT
GGCCTTCAGCAGGGTGATCTGGTCGCTCTGGCTGATCTTGCTGAAGCCGGGCAGGCCCTTGGCGAACTCCACGAT
CAGCTGCACGGTCAGGATGGTCATCTCGGTGATCTGGCGGAAGGGCAGGTCGCTCTCCTCGTCCTCCTCGTCGCT
CTGCCAGGTCTGGGTCACGCGCTTCAGGTCCTCGTCGCTGGGCTGCTCGTAGCCCTCCTGGTACCACACCAGGCG
GGCGATCAGGCTCTTCTGGTTGGCGCTCAGGGGGGGGATGTTCTTCTGGCGGTTCTGCTCCATCAGCTTCTCGCT
CAGGTAGCGGGGCACCACCTCGTGGATGCGGGCGGCCTCGGGGGGGGGGGGGTCGCACTGCATGATGGGGGGCAT
GTGGTCCTCCACGGTGGTGGTGCTCACGGGCAGCAGGATGCCCTTGTCCTTCTTCTGGCGCTGGCGGTCCTTGTT
CTTGCTGGGCTCCTGTATGACGCATTCCGGCCGCATACCCACGGCCAGGCACTTTTTCAGGCGGCACTCCTGACA
CTTTCGCCTCATGTACATGTCCATTTCGCAGGCGCGCCCGAACTTGCAGCAGTAGACGGCGCTCTTCGTAACGCT
GCGTCGAAAGAACACCTTGCAGGATCCACAGGTGAGGGCGTTGTAGTGGTAGCCGGAGGCCCTGTCGCCGCAAAC
CAGGCACAGCTCCTCTTGCACCCGTGGCGCAGGTCCCTTCTTGCTCTTCTTCGCATCGCAGCTTTCGTTCGCCGA
GTATCCGTTCAAGCTGCTCGAAGGCGAGAGATCATCGCGACCTGAAGATATAGAATTTGATATTCTTCTAGAGGT
ACCTAGAAGCTTCCCACCGTACTCGTCAATTCCAAGGGCATCGGTAAACATCTGCTCAAACTCGAAGTCGGCCAT
ATCCAGAGCGCCGTAGGGGGCGGAGTCGTGGGGGGTAAATCCCGGACCTGGGGAATCCCCGTCCCCCAACATGTC
CAGATCGAAATCGTCTAGCGCGTCGGCATGCGCCATCGCCACGTCCTCGCCGTCTAAGTGGAGTTCGTCCCCCAG
GCTGACATCGGTCGGGGGGGCCATGGTGGCCTCAGAGGCCAGCTTGGGGTAGTTTTCACGACACCTTAAATGGAA
GAAAAAAACTTTGAACCACTGTCTGAGGCTTGAGAATGAACCAAGATCCAAACTCAAAAAGGGCAAATTCCAAGG
AGAATTACATCAAGTGCCAAGCTGGCCTAACTTCAGTCTCCACCCACTCAGTATGGGGAAACTCCATCGCATAAA
ACCCCTCCCCCCAACCTAAAGACGACGTACTCCAAAAGCTCGAGAACTAATCGAGGTGCCTGGACGGCGCCCGGT
ACTCCGTGGAGTCACATGAAGCGACGGCTGAGGACGGAAAGGCCCTTTTCCTTTGTGTGGGTGACTCACCCGCCC
GCTCTCCCGAGCGCCGCGTCCTCCATTTTGAGCTCCCTGCAGCAGGGCCGGGAAGCGGCCATCTTTCCGCTCACG
CAACTGGTGCCGACCGGGCCAGCCTTGCCGCCCAGGGCGGGGCGATACACGGCGGCGCGAGGCCAGGCACCAGAG
CAGGCCGGCCAGCTTGAGACTACCCCCGTCCGATTCTCGGTGGCCGCGCTCGCAGGCCCCGCCTCGCCGAACATG
TGCGCTGGGACGCACGGGCCCCGTCGCCGCCCGCGGCCCCAAAAACCGAAATACCAGTGTGCAGATCTTGGCCCG
CATTTACAAGACTATCTTGCCAGAAAAAAAGCGTCGCAGCAGGTCATCAAAAATTTTAAATGGCTAGAGACTTAT
CGAAAGCAGCGAGACAGGCGCGAAGGTGCCACCAGATTCGCACGCGGCGGCCCCAGCGCCCAGGCCAAGCCTCAA
CTCAAGCACGAGGCGAAGGGGCTCCTTAAGCGCAAGGCCTCGAACTCTCCCACCCACTTCCAACCCGAAGCTCGG
GATCAAGAATCACGTACTGCAGCCAGGGGCGTGGAAGTAATTCAAGGCACGCAAGGGCCATAACCCGTAAAGAGG
CCAGGCCCGCGGGAACCACACACGGCACTTACCTGTGTTCTGGCGGCAAACCCGTTGCGAAAAAGAACGTTCACG
GCGACTACTGCACTTATATACGGTTCTCCCCCACCCTCGGGAAAAAGGCGGAGCCAGTACACGACATCACTTTCC
CAGTTTACCCCGCGCCACCTTCTCTAGGCACCGGTTCAATTGCCGACCCCTCCCCCCAACTTCTCGGGGACTGTG
GGCGATGTGCGCTCTGCCCACTGACGGGCACCGGAGCCTCTAGGGTCGACCTCGACGGATCGGGAGATCTCGGCC
GCATATTAAGTGCATTGTTCTCGATACCGCTAAGTGCATTGTTCTCGTTAGCTCGATGGACAAGTGCATTGTTCT
CTTGCTGAAAGCTCGATGGACAAGTGCATTGTTCTCTTGCTGAAAGCTCGATGGACAAGTGCATTGTTCTCTTGC
TGAAAGCTCGATGGACAAGTGCATTGTTCTCTTGCTGAAAGCTCAGTACCCGGGTCGGAGTACTGCCCCGCCCCT
AGCGATTAGCCCCGGCCCCGCATAGCTCCGCCCCGGGAGTACCCTCGACCGCCGGAGTATAAATAGAGGCGCTTC
GTCTACGGAGCGACAATTCAATTCAAACAAGCAAAGTGAACACGTCGCTAAGCGAAAGCTAAGCAAATAAACAAG
CGCAGCTGAACAAGCTAAACAATCTGCAGTAAAGTGCAAGTTAAAGTGAATCAATTAAAAGTAACCAGCAACCAA

Journal of Cell Science • Supplementary information

```
GTAAATCAACTGCAACTACTGAAATCTGCCAAGAAGTAATTATTGAATACAAGAAGAGAACTCTGAATACTTTCA
ACAAGTTACCGAGAAAGAAGAACTCACACACAGCTAGCCACAATGGACTACAAAGACGATGACGATAAAGCCATG
CTGGAAGTTCTGTTTCAGGGGCCCATGGTGAGCAAGGGCGAGGAGCTGTTCACCGGGGTGGTGCCCATCCTGGTC
GAGCTGGACGGCGACGTAAACGGCCACAAGTTCAGCGTGTCCGGCGAGGGCGAGGGCGATGCCACCTACGGCAAG
CTGACCCTGAAGTTCATCTGCACCACCGGCAAGCTGCCCGTGCCCTGGCCCACCCTCGTGACCACCCTGACCTAC
GGCGTGCAGTGCTTCAGCCGCTACCCCGACCACATGAAGCAGCACGACTTCTTCAAGTCCGCCATGCCCGAAGGC
TACGTCCAGGAGCGCACCATCTTCTTCAAGGACGACGGCAACTACAAGACCCGCGCCGAGGTGAAGTTCGAGGGC
GACACCCTGGTGAACCGCATCGAGCTGAAGGGCATCGACTTCAAGGAGGACGGCAACATCCTGGGGCACAAGCTG
GAGTACAACTACAACAGCCACAACGTCTATATCATGGCCGACAAGCAGAAGAACGGCATCAAGGTGAACTTCAAG
ATCCGCCACAACATCGAGGACGGCAGCGTGCAGCTCGCCGACCACTACCAGCAGAACACCCCCATCGGCGACGGC
CCCGTGCTGCTGCCCGACAACCACTACCTGAGCACCCAGTCCGCCCTGAGCAAAGACCCCAACGAGAAGCGCGAT
CACATGGTCCTGCTGGAGTTCGTGACCGCCGCCGGGATCACTCTCGGCATGGACGAGCTGTACAAGGAATTCGGA
GTGCAGGTGGAAACCATCTCCCCAGGAGACGGGCGCACCTTCCCCAAGCGCGGCCAGACCTGTGTGGTGCACTAC
ACCGGGATGCTTGAAGATGGAAAGAAAGTCGATTCCTCCCGGGACAGAAACAAGCCCTTTAAGTTTATGCTAGGC
AAGCAGGAGGTGATCCGAGGCTGGGAAGAAGGGGTTGCCCAGATGAGTGTGGGTCAGAGAGCCAAACTGACTATA
TCTCCAGATTATGCCTATGGTGCCACTGGGCACCCAGGCATCATCCCACCACATGCCACTCTCGTCTTCGATGTG
GAGCTTCTAAAACCGGAAGCCTAGTTCACTCCTCAGGTGCAGGCTGCCTATCAGAAGGTGGTGGCTGGTGTGGCC
AATGCCCTGGCTCACAAATACCACTGAGATCTTTTTCCCTCTGCCAAAAATTATGGGGACATCATGAAGCCCCTT
GAGCATCTGACTTCTGGCTAATAAAGGAAATTTATTTTCATTGCAATAGTGTGTTGGAATTTTTTGTGTCTCTCA
CTCGGAAGGACATATGGGAGGGCAAATCATTTAAAACATCAGAATGAGTATTTGGTTTAGAGTTTGGCAACATAT
GCCATATGCTGGCTGCCATGAACTAGCTACTCGGGACCCCTTACCGAAACATCGCCGCATTCTGCAGAGGAGTCG
AGTGTATGTAAACTTCTGACCCACTGGGAATGTGATGAAAGAAATAAAAGCTGAAATGAATCATTCTCTCTACTA
TTATTCTGATATTTCACATTCTTAAAATAAAGTGGTGATCCTAACTGACCTAAGACAGGGAATTTTTACTAGGAT
TAAATGTCAGGAATTGTGAAAAAGTGAGTTTAAATGTATTTGGCTAAGGTGTATGTAAACTTCCGACTTCAACTG
TATAGGGATCCGCTTCCTCGCTCACTGACTCGCTGCGCTCGGTCGTTCGGCTGCGGCGAGCGGTATCAGCTCACT
CAAAGGCGGTAATACGGTTATCCACAGAATCAGGGGATAACGCAGGAAAGAACATGTGAGCAAAAGGCCAGCAAA
AGGCCAGGAACCGTAAAAAGGCCGCGTTGCTGGCGTTTTTCCATAGGCTCCGCCCCCCTGACGAGCATCACAAAA
ATCGACGCTCAAGTCAGAGGTGGCGAAACCCGACAGGACTATAAAGATACCAGGCGTTTCCCCCTGGAAGCTCCC
TCGTGCGCTCTCCTGTTCCGACCCTGCCGCTTACCGGATACCTGTCCGCCTTTCTCCCTTCGGGAAGCGTGGCGC
TTTCTCATAGCTCACGCTGTAGGTATCTCAGTTCGGTGTAGGTCGTTCGCTCCAAGCTGGGCTGTGTGCACGAAC
CCCCCGTTCAGCCCGACCGCTGCGCCTTATCCGGTAACTATCGTCTTGAGTCCAACCCGGTAAGACACGACTTAT
CGCCACTGGCAGCAGCCACTGGTAACAGGATTAGCAGAGCGAGGTATGTAGGCGGTGCTACAGAGTTCTTGAAGT
GGTGGCCTAACTACGGCTACACTAGAAGAACAGTATTTGGTATCTGCGCTCTGCTGAAGCCAGTTACCTTCGGAA
AAAGAGTTGGTAGCTCTTGATCCGGCAAACAAACCACCGCTGGTAGCGGTGGTTTTTTTGTTTGCAAGCAGCAGA
TTACGCGCAGAAAAAAAGGATCTCAAGAAGATCCTTTGATCTTTTCTACGGGGTCTGACGCTCAGTGGAACGAAA
ACTCACGTTAAGGGATTTTGGTCATGAGATTATCAAAAAGGATCTTCACCTAGATCCTTTTAAATTAAAAATGAA
GTTTTAAATCAATCTAAAGTATATATGAGTAAACTTGGTCTGACAGTTACCAATGCTTAATCAGTGAGGCACCTA
TCTCAGCGATCTGTCTATTTCGTTCATCCATAGTTGCCTGACTCCCCGTCGTGTAGATAACTACGATACGGGAGG
GCTTACCATCTGGCCCCAGTGCTGCAATGATACCGCGAGACCCACGCTCACCGGCTCCAGATTTATCAGCAATAA
ACCAGCCAGCCGGAAGGGCCGAGCGCAGAAGTGGTCCTGCAACTTTATCCGCCTCCATCCAGTCTATTAATTGTT
GCCGGGAAGCTAGAGTAAGTAGTTCGCCAGTTAATAGTTTGCGCAACGTTGTTGCCATTGCTACAGGCATCGTGG
TGTCACGCTCGTCGTTTGGTATGGCTTCATTCAGCTCCGGTTCCCAACGATCAAGGCGAGTTACATGATCCCCCA
TGTTGTGCAAAAAAGCGGTTAGCTCCTTCGGTCCTCCGATCGTTGTCAGAAGTAAGTTGGCCGCAGTGTTATCAC
TCATGGTTATGGCAGCACTGCATAATTCTCTTACTGTCATGCCATCCGTAAGATGCTTTTCTGTGACTGGTGAGT
ACTCAACCAAGTCATTCTGAGAATAGTGTATGCGGCGACCGAGTTGCTCTTGCCCGGCGTCAATACGGGATAATA
CCGCGCCACATAGCAGAACTTTAAAAGTGCTCATCATTGGAAAACGTTCTTCGGGGCGAAAACTCTCAAGGATCT
TACCGCTGTTGAGATCCAGTTCGATGTAACCCACTCGTGCACCCAACTGATCTTCAGCATCTTTTACTTTCACCA
GCGTTTCTGGGTGAGCAAAAACAGGAAGGCAAAATGCCGCAAAAAAGGGAATAAGGGCGACACGGAAATGTTGAA
TACTCATACTCTTCCTTTTTCAATATTATTGAAGCATTTATCAGGGTTATTGTCTCATGAGCGGATACATATTTG
AATGTATTTAGAAAAATAAACAAATAGGGGTTCCGCGCACATTTCCCCGAAAAGTGCCACCTGATGCGGTGTGAA
ATACCGCACAGATGCGTAAGGAGAAAATACCGCATCAGGAAATTGTAAGCGTTAATATTTTGTTAAAATTGGATC
CCTATA
```

Fig. S5 cont.

pDBEcR-pIND(SP1)-DD/Bla.

Graphic map, cloning sites, locations of different elements, and complete DNA sequence. The vector expresses FLAG-tagged EGFP-DD fusion protein under the control of a modified ecdysone promoter. DBEcR (myc-tagged at C-terminus) and blasticidin resistance gene are expressed from a constitutive EF1α promoter. FLAG-EGFP can be excised with NheI and NotI/EcoRI and replaced with other cDNAs.

Journal of Cell Science • Supplementary information

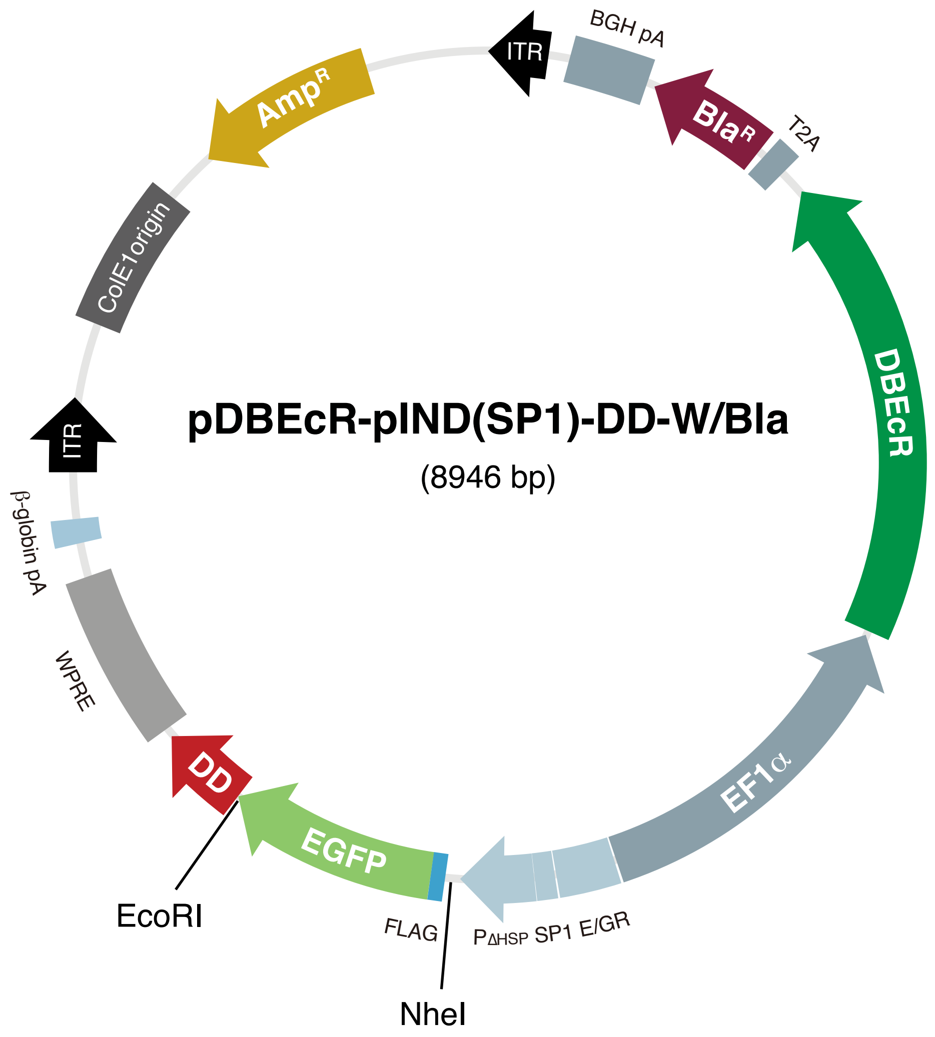

Feature	Location (bases)
Sleeping Beauty 5′ inverted terminal repeat	1–227
BGH polyadenylation signal	283–558
Blasticidin resistance gene	595–993
T2A ribosomal skipping site	997–1059
DBEcR	1207–2799
EF1α promoter	2821–4013
E/GRE ecdysone/glucocorticoid response element x6	4058–4231
SP1 specificity Protein 1 enhancer x3	4265–4312
$P_{\Delta HSP}$ minimal heat shock protein promoter	4327–4565
FLAG-tagged EGFP	4618–5391
DD-tag	5398–5724
WPRE	5735–6323
β-globin polyadenylation signal	6485–6539
Sleeping Beauty 3′ inverted terminal repeat	6690–6915
ColE1 origin	7103–7691
Ampicillin resistance gene	7865–8524

Journal of Cell Science • Supplementary information

pDBEcR-pIND(SP1)-DD-W/Bla

Region containing FLAG (grey), EGFP (green), and DD-tag (red).
Unique restriction enzyme sites are indicated.

```
 NheI          FLAG                                                 EGFP
             M  D  Y  K  D  D  D  D  K  A  M  L  E  V  L  F  Q  G  P  M  V  S  K  G  E  E
 GCTAGCCACAATGGACTACAAAGACGATGACGATAAAGCCATGCTGGAAGTTCTGTTTCAGGGGCCCATGGTGAGCAAGGGCGAGGAG

 L  F  T  G  V  V  P  I  L  V  E  L  D  G  D  V  N  G  H  K  F  S  V  S  G  E  G  E  G  D
CTGTTCACCGGGGTGGTGCCCATCCTGGTCGAGCTGGACGGCGACGTAAACGGCCACAAGTTCAGCGTGTCCGGCGAGGGCGAGGGCGAT

 A  T  Y  G  K  L  T  L  K  F  I  C  T  T  G  K  L  P  V  P  W  P  T  L  V  T  T  L  T  Y
GCCACCTACGGCAAGCTGACCCTGAAGTTCATCTGCACCACCGGCAAGCTGCCCGTGCCCTGGCCCACCCTCGTGACCACCCTGACCTAC

 G  V  Q  C  F  S  R  Y  P  D  H  M  K  Q  H  D  F  F  K  S  A  M  P  E  G  Y  V  Q  E  R
GGCGTGCAGTGCTTCAGCCGCTACCCCGACCACATGAAGCAGCACGACTTCTTCAAGTCCGCCATGCCCGAAGGCTACGTCCAGGAGCGC

 T  I  F  F  K  D  D  G  N  Y  K  T  R  A  E  V  K  F  E  G  D  T  L  V  N  R  I  E  L  K
ACCATCTTCTTCAAGGACGACGGCAACTACAAGACCCGCGCCGAGGTGAAGTTCGAGGGCGACACCCTGGTGAACCGCATCGAGCTGAAG

 G  I  D  F  K  E  D  G  N  I  L  G  H  K  L  E  Y  N  Y  N  S  H  N  V  Y  I  M  A  D  K
GGCATCGACTTCAAGGAGGACGGCAACATCCTGGGGCACAAGCTGGAGTACAACTACAACAGCCACAACGTCTATATCATGGCCGACAAG

 Q  K  N  G  I  K  V  N  F  K  I  R  H  N  I  E  D  G  S  V  Q  L  A  D  H  Y  Q  Q  N  T
CAGAAGAACGGCATCAAGGTGAACTTCAAGATCCGCCACAACATCGAGGACGGCAGCGTGCAGCTCGCCGACCACTACCAGCAGAACACC

 P  I  G  D  G  P  V  L  L  P  D  N  H  Y  L  S  T  Q  S  A  L  S  K  D  P  N  E  K  R  D
CCCATCGGCGACGGCCCCGTGCTGCTGCCCGACAACCACTACCTGAGCACCCAGTCCGCCCTGAGCAAAGACCCCAACGAGAAGCGCGAT
                                                                           EcoRI  DD
 H  M  V  L  L  E  F  V  T  A  A  G  I  T  L  G  M  D  E  L  Y  K  E  F  G  V  Q  V  E  T
CACATGGTCCTGCTGGAGTTCGTGACCGCCGCCGGGATCACTCTCGGCATGGACGAGCTGTACAAGGAATTCGGAGTGCAGGTGGAAACC

 I  S  P  G  D  G  R  T  F  P  K  R  G  Q  T  C  V  V  H  Y  T  G  M  L  E  D  G  K  K  V
ATCTCCCCAGGAGACGGGCGCACCTTCCCCAAGCGCGGCCAGACCTGTGTGGTGCACTACACCGGGATGCTTGAAGATGGAAAGAAAGTC

 D  S  S  R  D  R  N  K  P  F  K  F  M  L  G  K  Q  E  V  I  R  G  W  E  E  G  V  A  Q  M
GATTCCTCCCGGGACAGAAACAAGCCCTTTAAGTTTATGCTAGGCAAGCAGGAGGTGATCCGAGGCTGGGAAGAAGGGGTTGCCCAGATG

 S  V  G  Q  R  A  K  L  T  I  S  P  D  Y  A  Y  G  A  T  G  H  P  G  I  I  P  P  H  A  T
AGTGTGGGTCAGAGAGCCAAACTGACTATATCTCCAGATTATGCCTATGGTGCCACTGGGCACCCAGGCATCATCCCACCACATGCCACT

 L  V  F  D  V  E  L  L  K  P  E  A  *
CTCGTCTTCGATGTGGAGCTTCTAAAACCGGAAGCCTAG
```

Journal of Cell Science • Supplementary information

pDBEcR-pIND(SP1)-DD-W/Bla

CAGTTGAAGTCGGAAGTTTACATACACTTAAGTTGGAGTCATTAAAACTCGTTTTTCAACTACTCCACAAATTTC
TTGTTAACAAACAATAGTTTTGGCAAGTCAGTTAGGACATCTACTTTGTGCATGACACAAGTCATTTTTCCAACA
ATTGTTTACAGACAGATTATTTCACTTATAATTCACTGTATCACAATTCCAGTGGGTCAGAAGTTTACATACACT
AAGTTCGACTCCTCTGCAGAATGCGGCGATGTTTCGGTAAGGGGTCCGCTATCTAGTAGGCCCAGCTGGTTCTTT
CCGCCTCAGAAGCCATAGAGCCCACCGCATCCCCAGCATGCCTGCTATTGTCTTCCCAATCCTCCCCCTTGCTGT
CCTGCCCCACCCCACCCCCCAGAATAGAATGACACCTACTCAGACAATGCGATGCAATTTCCTCATTTTATTAGG
AAAGGACAGTGGGAGTGGCACCTTCCAGGGTCAAGGAAGGCACGGGGGAGGGGCAAACAACAGATGGCTGGCAAC
TAGAAGGCACAGTCGAGGCTGATCAGCGAGCTCTAGAGAATTGATCCCCAAGCTTCGGCCACGAAGTGCTTAGCC
CTCCCACACATAACCAGAGGGCAGCAATTCACGAATCCCAACTGCCGTCGGCTGTCCATCACTGTCCTTCACTAT
GGCTTTGATCCCAGGATGCAGATCGAGAAGCACCTGTCGGCACCGTCCGCAGGGGCTCAAGATGCCCCTGTTCTC
ATTTCCGATCGCGACGATACAAGTCAGGTTGCCAGCTGCCGCAGCAGCAGCAGTGCCCAGCACCACGAGTTCTGC
ACAAGGTCCCCCAGTAAAATGATATACATTGACACCAGTGAAGATGCGGCCGTCGCTAGAGAGAGCTGCGCTGGC
GACGCTGTAGTCTTCAGAGATGGGGATGCTGTTGATTGTAGCCGTTGCTCTTTCAATGAGGGTGGATTCTTCTTG
AGACAAAGGCTTGGCCATCGATGGGCCAGGATTCTCCTCGACGTCACCGCATGTTAGCAGACTTCCTCTGCCCTC
TCCACTACCAGACTTGCTAGTGGATCCGTTCAAGTCTTCTTCTGAGATTAATTTTTGTTCACCGTTCAAGTCTTC
CTCGGAGATTAGCTTTTGTTCACCGTTCAAATCTTCTTCAGAAATCAACTTTTGTTCACCACTTGCGGCAGAACC
GGATCCCAGCACCACGGGGTTGGTGGGGGGCAGCACGGTGGGGTGGGTGGTGGCCACCTCGGCCACGTCCCAGAT
CTCCTCCAGGAAGGGGGGCAGCTTGCGGTTCTTCAGCTTCAGGCTGATGCACATGTTGCTGTTCTGGGTGCCCAG
GGTGCGCAGCTCGGTCAGCACGCTCAGGATGCGGCCGTAGATCACGGCGCAGCGGCTGCTGGCGCTGTTCTGGTT
GATGATGTAGATGCGCAGGGTGTTCAGGTAGTAGCGCTGGATCTCCTCCACCAGGCTGGGCTGCTCCAGGCCGGG
GCGGTCGCTGAAGATCACGATGGCGGTCAGCAGGGCGAAGTGCACGTTGTCCATGCCCATCGCGAACATGCAGCG
GCAGAAGTGCAGCAGGTCCTCGATCACGTAGGCCATGCCGCCCTGGCGGTAGTTGTCGCGGGTGTAGGCCTTGTT
GTTGGCGAACAGCACGCTGTCGCTGGCGGCGTCGTAGCGGCGGGCCACGCGCAGCATCATCACCTCGCTGCTGCT
GGCCTTCAGCAGGGTGATCTGGTCGCTCTGGCTGATCTTGCTGAAGCCGGGCAGGCCCTTGGCGAACTCCACGAT
CAGCTGCACGGTCAGGATGGTCATCTCGGTGATCTGGCGGAAGGGCAGGTCGCTCTCCTCGTCCTCCTCGTCGCT
CTGCCAGGTCTGGGTCACGCGCTTCAGGTCCTCGTCGCTGGGCTGCTCGTAGCCCTCCTGGTACCACACCAGGCG
GGCGATCAGGCTCTTCTGGTTGGCGCTCAGGGGGGGGATGTTCTTCTGGCGGTTCTGCTCCATCAGCTTCTCGCT
CAGGTAGCGGGGCACCACCTCGTGGATGCGGGCGGCCTCGGGGGGGGGGGGTCGCACTGCATGATGGGGGGCAT
GTGGTCCTCCACGGTGGTGGTGCTCACGGGCAGCAGGATGCCCTTGTCCTTCTTCTGGCGCTGGCGGTCCTTGTT
CTTGCTGGGCTCCTGTATGACGCATTCCGGCCGCATACCCACGGCCAGGCACTTTTTCAGGCGGCACTCCTGACA
CTTTCGCCTCATGTACATGTCCATTTCGCAGGCGCGCCCGAACTTGCAGCAGTAGACGGCGCTCTTCGTAACGCT
GCGTCGAAAGAACACCTTGCAGGATCCACAGGTGAGGGCGTTGTAGTGGTAGCCGGAGGCCCTGTCGCCGCAAAC
CAGGCACAGCTCCTCTTGCACCCGTGGCGCAGGTCCCTTCTTGCTCTTCTTCGCATCGCAGCTTTCGTTCGCCGA
GTATCCGTTCAAGCTGCTCGAAGGCGAGAGATCATCGCGACCTGAAGATATAGAATTTGATATTCTTCTAGAGGT
ACCTAGAAGCTTCCCACCGTACTCGTCAATTCCAAGGGCATCGGTAAACATCTGCTCAAACTCGAAGTCGGCCAT
ATCCAGAGCGCCGTAGGGGGCGGAGTCGTGGGGGGTAAATCCCGGACCTGGGGAATCCCCGTCCCCCAACATGTC
CAGATCGAAATCGTCTAGCGCGTCGGCATGCGCCATCGCCACGTCCTCGCCGTCTAAGTGGAGTTCGTCCCCCAG
GCTGACATCGGTCGGGGGGGCCATGGTGGCCTCAGAGGCCAGCTTGGGGTAGTTTTCACGACACCTTAAATGGAA
GAAAAAAACTTTGAACCACTGTCTGAGGCTTGAGAATGAACCAAGATCCAAACTCAAAAAGGGCAAATTCCAAGG
AGAATTACATCAAGTGCCAAGCTGGCCTAACTTCAGTCTCCACCCACTCAGTATGGGGAAACTCCATCGCATAAA
ACCCCTCCCCCCAACCTAAAGACGACGTACTCCAAAAGCTCGAGAACTAATCGAGGTGCCTGGACGGCGCCCGGT
ACTCCGTGGAGTCACATGAAGCGACGGCTGAGGACGGAAAGGCCCTTTTCCTTTGTGTGGGTGACTCACCCGCCC
GCTCTCCCGAGCGCCGCGTCCTCCATTTTGAGCTCCCTGCAGCAGGGCCGGGAAGCGGCCATCTTTCCGCTCACG
CAACTGGTGCCGACCGGGCCAGCCTTGCCGCCCAGGGCGGGGCGATACACGGCGGCGCGAGGCCAGGCACCAGAG
CAGGCCGGCCAGCTTGAGACTACCCCCGTCCGATTCTCGGTGGCCGCGCTCGCAGGCCCCGCCTCGCCGAACATG
TGCGCTGGGACGCACGGGCCCCGTCGCCGCCCGCGGCCCCAAAAACCGAAATACCAGTGTGCAGATCTTGGCCCG
CATTTACAAGACTATCTTGCCAGAAAAAAAGCGTCGCAGCAGGTCATCAAAAATTTTAAATGGCTAGAGACTTAT
CGAAAGCAGCGAGACAGGCGCGAAGGTGCCACCAGATTCGCACGCGGCGGCCCCAGCGCCCAGGCCAAGCCTCAA
CTCAAGCACGAGGCGAAGGGGCTCCTTAAGCGCAAGGCCTCGAACTCTCCCACCCACTTCCAACCCGAAGCTCGG
GATCAAGAATCACGTACTGCAGCCAGGGGCGTGGAAGTAATTCAAGGCACGCAAGGGCCATAACCCGTAAAGAGG
CCAGGCCCGCGGGAACCACACACGGCACTTACCTGTGTTCTGGCGGCAAACCCGTTGCGAAAAAGAACGTTCACG
GCGACTACTGCACTTATATACGGTTCTCCCCCACCCTCGGGAAAAAGGCGGAGCCAGTACACGACATCACTTTCC
CAGTTTACCCCGCGCCACCTTCTCTAGGCACCGGTTCAATTGCCGACCCCTCCCCCCAACTTCTCGGGGACTGTG
GGCGATGTGCGCTCTGCCCACTGACGGGCACCGGAGCCTCTAGGGTCGACCTCGACGGATCGGGAGATCTCGGCC
GCATATTAAGTGCATTGTTCTCGATACCGCTAAGTGCATTGTTCTCGTTAGCTCGATGGACAAGTGCATTGTTCT
CTTGCTGAAAGCTCGATGGACAAGTGCATTGTTCTCTTGCTGAAAGCTCGATGGACAAGTGCATTGTTCTCTTGC
TGAAAGCTCGATGGACAAGTGCATTGTTCTCTTGCTGAAAGCTCAGTACCCGGGTCGGAGTACTGCCCCGCCCCT
AGCGATTAGCCCCGGCCCCGCATAGCTCCGCCCCGGGAGTACCCTCGACCGCCGGAGTATAAATAGAGGCGCTTC
GTCTACGGAGCGACAATTCAATTCAAACAAGCAAAGTGAACACGTCGCTAAGCGAAAGCTAAGCAAATAAACAAG
CGCAGCTGAACAAGCTAAACAATCTGCAGTAAAGTGCAAGTTAAAGTGAATCAATTAAAAGTAACCAGCAACCAA

Journal of Cell Science • Supplementary information

GTAAATCAACTGCAACTACTGAAATCTGCCAAGAAGTAATTATTGAATACAAGAAGAGAACTCTGAATACTTTCA
ACAAGTTACCGAGAAAGAAGAACTCACACACAGCTAGCCACAATGGACTACAAAGACGATGACGATAAAGCCATG
CTGGAAGTTCTGTTTCAGGGGCCCATGGTGAGCAAGGGCGAGGAGCTGTTCACCGGGGTGGTGCCCATCCTGGTC
GAGCTGGACGGCGACGTAAACGGCCACAAGTTCAGCGTGTCCGGCGAGGGCGAGGGCGATGCCACCTACGGCAAG
CTGACCCTGAAGTTCATCTGCACCACCGGCAAGCTGCCCGTGCCCTGGCCCACCCTCGTGACCACCCTGACCTAC
GGCGTGCAGTGCTTCAGCCGCTACCCCGACCACATGAAGCAGCACGACTTCTTCAAGTCCGCCATGCCCGAAGGC
TACGTCCAGGAGCGCACCATCTTCTTCAAGGACGACGGCAACTACAAGACCCGCGCCGAGGTGAAGTTCGAGGGC
GACACCCTGGTGAACCGCATCGAGCTGAAGGGCATCGACTTCAAGGAGGACGGCAACATCCTGGGGCACAAGCTG
GAGTACAACTACAACAGCCACAACGTCTATATCATGGCCGACAAGCAGAAGAACGGCATCAAGGTGAACTTCAAG
ATCCGCCACAACATCGAGGACGGCAGCGTGCAGCTCGCCGACCACTACCAGCAGAACACCCCCATCGGCGACGGC
CCCGTGCTGCTGCCCGACAACCACTACCTGAGCACCCAGTCCGCCCTGAGCAAAGACCCCAACGAGAAGCGCGAT
CACATGGTCCTGCTGGAGTTCGTGACCGCCGCCGGGATCACTCTCGGCATGGACGAGCTGTACAAGGAATTCGGA
GTGCAGGTGGAAACCATCTCCCCAGGAGACGGGCGCACCTTCCCCAAGCGCGGCCAGACCTGTGTGGTGCACTAC
ACCGGGATGCTTGAAGATGGAAAGAAAGTCGATTCCTCCCGGGACAGAAACAAGCCCTTTAAGTTTATGCTAGGC
AAGCAGGAGGTGATCCGAGGCTGGGAAGAAGGGGTTGCCCAGATGAGTGTGGGTCAGAGAGCCAAACTGACTATA
TCTCCAGATTATGCCTATGGTGCCACTGGGCACCCAGGCATCATCCCACCACATGCCACTCTCGTCTTCGATGTG
GAGCTTCTAAAACCGGAAGCCTAGTCTTATCGGTAATCAACCTCTGGATTACAAAATTTGTGAAAGATTGACTGG
TATTCTTAACTATGTTGCTCCTTTTACGCTATGTGGATACGCTGCTTTAATGCCTTTGTATCATGCTATTGCTTC
CCGTATGGCTTTCATTTTCTCCTCCTTGTATAAATCCTGGTTGCTGTCTCTTTATGAGGAGTTGTGGCCCGTTGT
CAGGCAACGTGGCGTGGTGTGCACTGTGTTTGCTGACGCAACCCCCACTGGTTGGGGCATTGCCACCACCTGTCA
GCTCCTTTCCGGGACTTTCGCTTTCCCCCTCCCTATTGCCACGGCGGAACTCATCGCCGCCTGCCTTGCCCGCTG
CTGGACAGGGGCTCGGCTGTTGGGCACTGACAATTCCGTGGTGTTGTCGGGGAAATCATCGTCCTTTCCTTGGCT
GCTCGCCTGTGTTGCCACCTGGATTCTGCGCGGGACGTCCTTCTGCTACGTCCCTTCGGCCCTCAATCCAGCGGA
CCTTCCTTCCCGCGGCCTGCTGCCGGCTCTGCGGCCTCTTCCGCGTCTTCGCCTTCGCCCTCAGACGAGTCGGAT
CTCCCTTTGGGCCGCCTCCCCGCATCGATACCGTCGCAATTCACTCCTCAGGTGCAGGCTGCCTATCAGAAGGTG
GTGGCTGGTGTGGCCAATGCCCTGGCTCACAAATACCACTGAGATCTTTTTCCCTCTGCCAAAAATTATGGGGAC
ATCATGAAGCCCCTTGAGCATCTGACTTCTGGCTAATAAAGGAAATTTATTTTCATTGCAATAGTGTGTTGGAAT
TTTTTGTGTCTCTCACTCGGAAGGACATATGGGAGGGCAAATCATTTAAAACATCAGAATGAGTATTTGGTTTAG
AGTTTGGCAACATATGCCATATGCTGGCTGCCATGAACTAGCTACTCGGGACCCCTTACCGAAACATCGCCGCAT
TCTGCAGAGGAGTCGAGTGTATGTAAACTTCTGACCCACTGGGAATGTGATGAAAGAAATAAAAGCTGAAATGAA
TCATTCTCTCTACTATTATTCTGATATTTCACATTCTTAAAATAAAGTGGTGATCCTAACTGACCTAAGACAGGG
AATTTTTACTAGGATTAAATGTCAGGAATTGTGAAAAAGTGAGTTTAAATGTATTTGGCTAAGGTGTATGTAAAC
TTCCGACTTCAACTGTATAGGGATCCGCTTCCTCGCTCACTGACTCGCTGCGCTCGGTCGTTCGGCTGCGGCGAG
CGGTATCAGCTCACTCAAAGGCGGTAATACGGTTATCCACAGAATCAGGGGATAACGCAGGAAAGAACATGTGAG
CAAAAGGCCAGCAAAAGGCCAGGAACCGTAAAAAGGCCGCGTTGCTGGCGTTTTTCCATAGGCTCCGCCCCCCTG
ACGAGCATCACAAAAATCGACGCTCAAGTCAGAGGTGGCGAAACCCGACAGGACTATAAAGATACCAGGCGTTTC
CCCCTGGAAGCTCCCTCGTGCGCTCTCCTGTTCCGACCCTGCCGCTTACCGGATACCTGTCCGCCTTTCTCCCTT
CGGGAAGCGTGGCGCTTTCTCATAGCTCACGCTGTAGGTATCTCAGTTCGGTGTAGGTCGTTCGCTCCAAGCTGG
GCTGTGTGCACGAACCCCCCGTTCAGCCCGACCGCTGCGCCTTATCCGGTAACTATCGTCTTGAGTCCAACCCGG
TAAGACACGACTTATCGCCACTGGCAGCAGCCACTGGTAACAGGATTAGCAGAGCGAGGTATGTAGGCGGTGCTA
CAGAGTTCTTGAAGTGGTGGCCTAACTACGGCTACACTAGAAGAACAGTATTTGGTATCTGCGCTCTGCTGAAGC
CAGTTACCTTCGGAAAAAGAGTTGGTAGCTCTTGATCCGGCAAACAAACCACCGCTGGTAGCGGTGGTTTTTTTG
TTTGCAAGCAGCAGATTACGCGCAGAAAAAAAGGATCTCAAGAAGATCCTTTGATCTTTTCTACGGGGTCTGACG
CTCAGTGGAACGAAAACTCACGTTAAGGGATTTTGGTCATGAGATTATCAAAAAGGATCTTCACCTAGATCCTTT
TAAATTAAAAATGAAGTTTTAAATCAATCTAAAGTATATATGAGTAAACTTGGTCTGACAGTTACCAATGCTTAA
TCAGTGAGGCACCTATCTCAGCGATCTGTCTATTTCGTTCATCCATAGTTGCCTGACTCCCCGTCGTGTAGATAA
CTACGATACGGGAGGGCTTACCATCTGGCCCCAGTGCTGCAATGATACCGCGAGACCCACGCTCACCGGCTCCAG
ATTTATCAGCAATAAACCAGCCAGCCGGAAGGGCCGAGCGCAGAAGTGGTCCTGCAACTTTATCCGCCTCCATCC
AGTCTATTAATTGTTGCCGGGAAGCTAGAGTAAGTAGTTCGCCAGTTAATAGTTTGCGCAACGTTGTTGCCATTG
CTACAGGCATCGTGGTGTCACGCTCGTCGTTTGGTATGGCTTCATTCAGCTCCGGTTCCCAACGATCAAGGCGAG
TTACATGATCCCCCATGTTGTGCAAAAAAGCGGTTAGCTCCTTCGGTCCTCCGATCGTTGTCAGAAGTAAGTTGG
CCGCAGTGTTATCACTCATGGTTATGGCAGCACTGCATAATTCTCTTACTGTCATGCCATCCGTAAGATGCTTTT
CTGTGACTGGTGAGTACTCAACCAAGTCATTCTGAGAATAGTGTATGCGGCGACCGAGTTGCTCTTGCCCGGCGT
CAATACGGGATAATACCGCGCCACATAGCAGAACTTTAAAAGTGCTCATCATTGGAAAACGTTCTTCGGGGCGAA
AACTCTCAAGGATCTTACCGCTGTTGAGATCCAGTTCGATGTAACCCACTCGTGCACCCAACTGATCTTCAGCAT
CTTTTACTTTCACCAGCGTTTCTGGGTGAGCAAAAACAGGAAGGCAAAATGCCGCAAAAAAGGGAATAAGGGCGA
CACGGAAATGTTGAATACTCATACTCTTCCTTTTTCAATATTATTGAAGCATTTATCAGGGTTATTGTCTCATGA
GCGGATACATATTTGAATGTATTTAGAAAAATAAACAAATAGGGGTTCCGCGCACATTTCCCCGAAAAGTGCCAC
CTGATGCGGTGTGAAATACCGCACAGATGCGTAAGGAGAAAATACCGCATCAGGAAATTGTAAGCGTTAATATTT
TGTTAAAATTGGATCCCTATA

Journal of Cell Science • Supplementary information

Fig. S5 cont.

pDBEcR-pIND(SP1)-DD/Bla.

Graphic map, cloning sites, locations of different elements, and complete DNA sequence. The vector expresses FLAG-tagged EGFP-DD fusion protein under the control of a modified ecdysone promoter. An WPRE element is present in at the 3' UTR. DBEcR (myc-tagged at C-terminus) and blasticidin resistance gene are expressed from a constitutive EF1α promoter. FLAG-EGFP can be excised with NheI and NotI/EcoRI and replaced with other cDNAs.

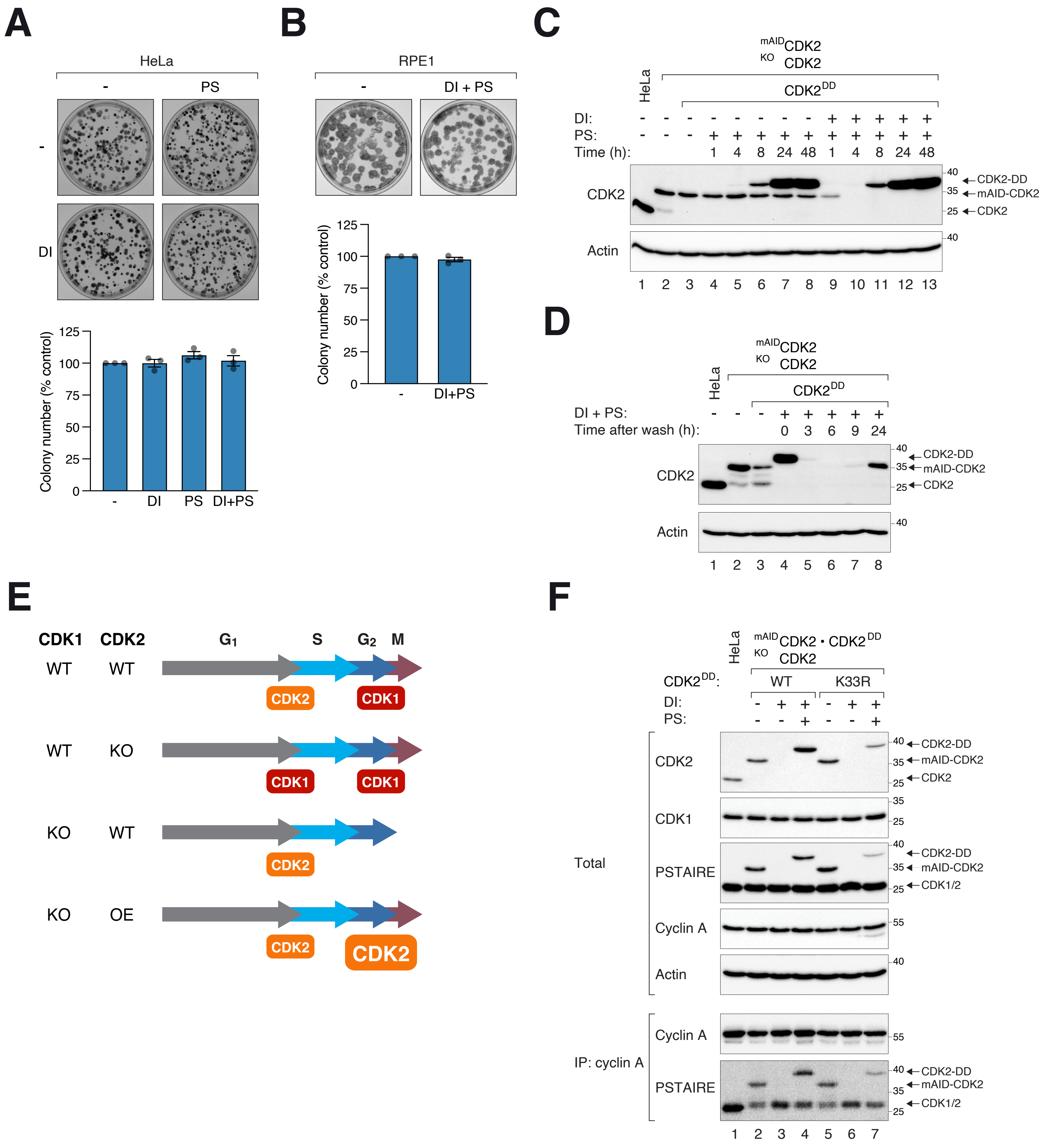

Journal of Cell Science • Supplementary information

Fig. S6. Gene-ON/OFF system.

(A) DI and PS do not affect long-term survival. HeLa cells were plated at low density and treated with DI and/or PS. After two weeks, the colonies were fixed and stained. Representative plates are shown (upper panel). The number of colonies was quantified (lower panel; mean -/+ SEM of three independent experiments).

(B) RPE1 cells were plated at low density and treated with DI and PS. After two weeks, the colonies were fixed and stained. Representative plates are shown (upper panel). The number of colonies was quantified (lower panel; mean -/+ SEM of three independent experiments).

(C) Independent on/off switches of CDK2. HeLa cells lacking endogenous CDK2 were generated by transfecting CRISPR-Cas9 and expressing ^{mAID}CDK2 under the control of the gene-OFF (tTA–AID) system. CDK2^{DD} under the control of the gene-ON (DBEcR–DD) system was further engineered into the cell line. The cells were incubated with DI and PS (10 μM PonA and 1 μM Shield-1; to turn off ^{mAID}CDK2 and turn on CDK2^{DD}, respectively) and harvested at the indicated time points for immunoblotting analysis. The parental cells (HeLa and ^{mAID}CDK2-expressing cells) were also loaded as controls.

(D) The reversibility of the gene-ON/OFF system. Cells expressing ^{mAID}CDK2 and CDK2^{DD} (see panel C) were incubated with DI and PS (10 μM PonA and 1 μM Shield-1) together for 24 h to turn off ^{mAID}CDK2 and turn on CDK2^{DD}, respectively. The cells were then washed with PBS (trypsinized, washed three times with PBS (10 ml each time)) and replated. The washing was repeated 1 h later. The cells were then harvested at the indicated time points (from first wash) for immunoblotting analysis.

(E) Model of the relationship between CDK1 and CDK2. The relationship between the expression of CDKs in KO cell lines according to Lau et al. (2021) [Cell Reports 37: 109808]. In normal HeLa cells, CDK1 and CDK2 are responsible in driving G_2/M and G_1/S, respectively. In the absence of CDK2, endogenous CDK1 is able to take over the role CDK2. However, CDK2 is insufficient to replace CDK1's G_2/M functions unless overexpressed (OE).

(F) Exchanging WT with kinase-dead mutant of CDK2. Two CDK2^{KO} cell lines expressing ^{mAID}CDK2 and either WT or K33R CDK2^{DD} were generated. The cells were treated with DI (to turn off ^{mAID}CDK2) and PS (to turn on CDK2^{DD}) for 24 h. Lysates were prepared and subjected to immunoprecipitation with cyclin A antibodies. Both total cell lysates and immunoprecipitates were analyzed with immunoblotting. The parental HeLa cells were also loaded as controls (containing endogenous CDK2). CDK1 and CDK2 were detected with both specific antibodies and a PSTAIRE monoclonal antibody, which recognizes an epitope present in both proteins. Endogenous CDK1 and CDK2 are similar in size. As lanes 2-7 do not contain endogenous CDK2, the PSTAIRE signals were from CDK1.

Journal of Cell Science • Supplementary information

Table S1. Plasmids.

Plasmid	Name	Purpose	Methods
1	pUHD-SB-mIAA7/Hyg	Tet-Off vector with SB ITRs for expressing mIAA7-tagged proteins; hygromycin resistance.	Ligation of NheI-NcoI-cut PCR product (primers 1-2; template: pSH-EFIRES-P-Seipin-miniIAA7-3XFlag (a gift from Elina Ikonen; Addgene #129722)) into NheI-NcoI-cut pUHD-SB-AID/Hyg (Yeung et al., 2021).
2	mAID-CDK2 in pUHD-SB-mAID/Hyg	Expression of ^{mAID}CDK2; hygromycin resistance.	Ligation of the NcoI-EcoRI fragment from FLAG-CDK2 in pUHD-P1 (Yam et al., 2000) into pUHD-SB-mAID/Hyg (Yeung et al., 2021).
3	mIAA7-CDK2 in pUHD-SB-mIAA7/Hyg	Expression of ^{mIAA7}CDK2; hygromycin resistance.	Ligation of the NcoI-EcoRI fragment from FLAG-CDK2 in pUHD-P1 (Yam et al., 2000) into Plasmid 1.
4	mIAA7(MV)-CDK2 in pUHD-SB-mIAA7(MV)/Hyg	Expression of $^{mIAA7(MV)}$CDK2; hygromycin resistance.	Insertion of double PCR products (first PCRs: primers 3-4 and 5-6, respectively; template: ^{mIAA7}CDK2 in pUHD-SB-mIAA7/Hyg; second PCR: primers 3-6) into NheI-cut Plasmid 3 using Seamless Ligation Cloning Extract (SLiCE) cloning method (Motohashi, 2015).
5	pSBbi-AFB2/Pur	Vector with SB ITRs for expressing AFB2 (myc-tagged); puromycin resistance.	Insertion of PCR product (primers 7-8; template: pSH-EFIRES-P-AtAFB2 (a gift from Elina Ikonen; Addgene #129715)) into NcoI-SalI-cut pSBbi-TIR1/Pur (Yeung et al., 2021) using SLiCE cloning (Motohashi, 2015).
6	Rosa26-LARA	Cloning intermediate.	Insertion of a double PCR fragment (first PCRs: primers 9-10 and 11-12, respectively; template: Rosa26_OsTIR1_Myc (a gift from Helfrid Hochegger, University of Sussex, UK); cut with AgeI followed by ligation; second PCR: primers 9-12) into BamHI-cut pSBbi-Pur (a gift from Eric Kowarz; Addgene #60523) using SLiCE cloning (Motohashi, 2015).
7	LacI-VP48 in Rosa26-LARA/Zeo	Vector with Rosa26 homology arms expressing LacI-VP48; zeocin resistance.	Insertion of AgeI-ClaI-cut PCR product (primers 13-14; template: pSBbi-LacI-VP48-TIR1/Pur (Lau et al., 2021)) and ClaI-XhoI cut PCR product (primers 13-14; template: pSBbi-TIR1-tTA/Zeo (Yeung et al., 2021)) into AgeI-SalI-cut Plasmid 6.
8	pmTol2-H2B-iRFP670nano/Zeo	Vector with miniTol2 ITRs for expressing histone H2B-iRFP670nano; zeocin resistance.	Insertion of two PCR products ((1) primers 15-16; template: Plasmid 7; (2) primers 17-18, template: pSBbi-LacI-VP48-TIR1/Pur (Lau et al., 2021)) and a double PCR product (primers 19-20; template: pH2B-miRFP670nano (a gift from Vladislav Verkhusha; Addgene #127438)) into BglII-NcoI-cut pminiTol2 (a gift from Stephen Ekker; Addgene #31829) using SLiCE cloning (Motohashi, 2015).
9	pmTol2-H2B-Clover/Zeo	Vector with miniTol2 ITRs for expressing histone H2B-Clover; zeocin resistance.	Insertion of BamHI-ClaI-cut double PCR product (first PCRs: primers 21-22; template: pAC1403-pCR8-Clover_NLSPUFc (a gift from Albert Cheng; Addgene #71901); primers 23-24; template: Plasmid 8; second PCR: primers 21-24) into BamHI-ClaI-cut Plasmid 8.
10	pmTol2-H2B-Clover-BGHpA/Zeo	Adding bovine growth hormone polyadenylation signal (BGHpA) to Plasmid 9.	PCR product (primers 25-26; template: pSBbi-Pur (a gift from Eric Kowarz; Addgene #60523)) was insert into HindIII-cut Plasmid 9 using SLiCE cloning (Motohashi, 2015).
11	pRPBSA-EF1-BGHpA	Cloning intermediate.	Insertion of SpeI-EcoRI-cut PCR product (primers 27-28; template: Plasmid 10) into SpeI-MfeI-cut pSH-EFIRES-P-AtAFB2 (a gift from Elina Ikonen; Addgene 129715).

Journal of Cell Science • Supplementary information

12	pRPBSA-EF1-AFB2-myc-BGHpA	Plasmid with AAVS1 homology arms expressing AFB2 (myc-tagged).	PCR product (primers 7-29; template: Plasmid 5) was inserted into NcoI-HindIII-cut Plasmid 11 using SLiCE cloning (Motohashi, 2015).
13	pRPBSA-EF1-AFB2-myc-BGHpA/Zeo	Plasmid with AAVS1 homology arms expressing AFB2 (myc-tagged); zeocin resistance.	Insertion of a PCR product (primers 30-31; template: Plasmid 10) into SpeI-cut Plasmid 12 using SLiCE cloning (Motohashi, 2015).
14	pAFB2-H2B-Clover/Zeo	Plasmid with AAVS1 homology arms expressing AFB2 and histone H2B-Clover; zeocin resistance.	Insertion of three PCR products ((1) primers 32-33; (2) primers 34-35; (3) primers 36-37; template: Plasmid 10) into HindIII-XbaI-cut Plasmid 13 using SLiCE cloning (Motohashi, 2015).
15	pSBbi-TIR1-tTA/Bla	Plasmid with SB ITRs for expressing TIR1 and tTA; blasticidin resistance.	Ligation of ClaI-EcoRI-cut PCR product (primers 38-39; template: CHK2(R145W)-V5 in pcDNA6/V5-HisA (a gift from Alexandra Brown, National Institutes of Health, USA)) into ClaI-EcoRI-cut pSBbi-TIR1-tTA/Pur (Yeung et al., 2021).
16	pSB-LacI-VP48-NLS-myc/Bla	Cloning intermediate.	Ligation of two PCR products ((1) primers 40-41; template pSBbi-TIR1/Pur (Yeung et al., 2021); then cut with EcoRI-SpeI; (2) first PCRs: primers 42-43; template: pSBbi-LacI-VP48-TIR1/Pur (Lau et al., 2021); primers 23-44; template: Plasmid 15; second PCR: primers 42-44; then cut with NheI-HindIII) into EcoRI-HindIII-cut pSBbi-TIR1/Pur (Yeung et al., 2021).
17	pSBbi-TIR1-myc-IKZF3/Bla	Cloning intermediate.	Insertion of PCR product (primers 45-46; template: pIRV-SB-IKZF3/Bla (Lau et al., 2021)) into HindIII-cut pSBbi-TIR1/Bla (Lau et al., 2021) using SLiCE cloning (Motohashi, 2015).
18	pSB-LacI-VP48-NLS-3xmyc-IKZF3/Bla	Cloning intermediate.	Insertion of double PCR product (first PCRs: primers 47-48; template: Plasmid 16; primers 49-50; template: Plasmid 17; second PCR: primers 47-50) and a PCR product (primers 51-52; template: Plasmid 16) into NotI-HindIII-cut Plasmid 16 using SLiCE cloning (Motohashi, 2015).
19	pSB-LacI-VP48-NLS-3xmyc-2xIKZF3/Bla	Cloning intermediate.	Insertion of two PCR products ((1) primers 53-54; template: Plasmid 18; (2) primers 47-55; template: Plasmid 18) into NotI-ClaI-cut Plasmid 18 using SLiCE cloning (Motohashi, 2015).
20	pSB-LacI-VP48-NLS-3xmyc/Bla	Cloning intermediate.	Insertion of two PCR products ((1) primers 47-56; template: Plasmid 19; (2) primers 23-51; template: Plasmid 19) into NotI-HindIII-cut Plasmid 16 using SLiCE cloning (Motohashi, 2015).
21	pSB-EF1-NcoI-3xmyc-T2A/Bla	Cloning intermediate.	Ligating of two PCR products ((1) primers 40-57; template: pSBbi-TIR1/Pur (Yeung et al., 2021); then cut with XhoI-NcoI; (2) primers 51-58; template: Plasmid 20; then cut with NcoI-HindIII) into XhoI-HindIII-cut Plasmid 20.
22	pDBEcR-RPBSA/Bla	Cloning intermediate.	Insertion of two PCR products ((1) primers 59-60; template: second half of DBEcR, synthesized by Sangon Biotech, Shanghai, China); (2) primers 61-62; template: pVgRXR, a gift from Dong-Yan Jin, The University of Hong Kong) into NcoI-SalI-cut pSBbi-TIR1/Bla (Lau et al., 2021).
23	pDBEcR/Bla	Plasmid with SB ITRs for expressing DBEcR (3xmyc-	Insertion of PCR product (primers 63-64; template: Plasmid 22) into NcoI-cut Plasmid 21 using SLiCE cloning (Motohashi, 2015).

Journal of Cell Science • Supplementary information

		tagged) under EF1α promoter; blasticidin resistance.	
24	EGFP in pIND(SP1)	Ecdysone-inducible vector for expressing EGFP.	Ligation of SalI-NheI-cut fragment from MAD2 in pIND(SP1) (a gift from Dong-Yan Jin, The University of Hong Kong) into XhoI-NheI-cut EGFP in pUHD-P3 (Ma et al., 2009).
25	pDBEcR-pIND(SP1)/Bla	Ecdysone-inducible vector with SB ITRs for expressing DBEcR (3xmyc-tagged) and FLAG-EGFP; blasticidin resistance.	Insertion of PCR product (primers 65-66; template: Plasmid 24) into EcoRI-cut Plasmid 23 using SLiCE cloning (Motohashi, 2015).
26	pDBEcR-pIND(SP1)-W/Bla	Ecdysone-inducible vector with SB ITRs for expressing FLAG-EGFP (WPRE in 3' UTR) and DBEcR (3xmyc-tagged); blasticidin resistance.	Insertion of PCR product (primers 67-68; template: lenti dCAS-VP64_Blast (GeneScript, Singapore) into EcoRI-cut Plasmid 25 using SLiCE cloning (Motohashi, 2015).
27	CDK2 in pDBEcR-pIND(SP1)/Bla	Ecdysone-inducible vector with SB ITRs for expressing CDK2 and DBEcR (3xmyc-tagged); blasticidin resistance.	Insertion of PCR product (primers 69-70; template: FLAG-CDK2 in pUHD-P1 (Yam et al., 2000) into NheI-EcoRI-cut Plasmid 25 using T5 exonuclease DNA assembly (TEDA) method (Xia et al., 2019).
28	CDK2(K33R) in pDBEcR-pIND(SP1)/Bla	Ecdysone-inducible vector with SB ITRs for expressing CDK2(K33R) and DBEcR (3xmyc-tagged); blasticidin resistance.	Insertion of PCR product (primers 69-70; template: FLAG-CDK2(K33R) in pUHD-P1 (Yam et al., 2000) into NheI-EcoRI-cut Plasmid 25 using T5 exonuclease DNA assembly (TEDA) method (Xia et al., 2019).
29	CDK2-DD in pDBEcR-pIND(SP1)/Bla	Ecdysone-inducible vector with SB ITRs for expressing $CDK2^{DD}$ and DBEcR (3xmyc-tagged); blasticidin resistance.	Insertion of PCR product (primers 71-72; template: pX330-BsaIx2-DD-Cas9 (a gift from Michele Calos; Addgene #75380)) into EcoRI-cut Plasmid 27 using SLiCE cloning (Motohashi, 2015).
30	CDK2(K33R)-DD in pDBEcR-pIND(SP1)/Bla	Ecdysone-inducible vector with SB ITRs for expressing $CDK2(K33R)^{DD}$ and DBEcR (3xmyc-tagged); blasticidin resistance.	Insertion of PCR product (primers 71-72; template: pX330-BsaIx2-DD-Cas9 (a gift from Michele Calos; Addgene #75380)) into EcoRI-cut Plasmid 28 using SLiCE cloning (Motohashi, 2015).
31	pDBEcR-pIND(SP1)-DD/Bla	Ecdysone-inducible vector with SB ITRs for expressing FLAG-$EGFP^{DD}$ and DBEcR (3xmyc-tagged); blasticidin resistance.	Insertion of PCR product (primers 73-74; template: Plasmid 29) into NotI-EcoRI-cut Plasmid 25 using SLiCE cloning (Motohashi, 2015).
32	pDBEcR-pIND(SP1)-DD-W/Bla	Ecdysone-inducible vector with SB ITRs for expressing FLAG-$EGFP^{DD}$ (WPRE in 3' UTR) and DBEcR (3xmyc-tagged); blasticidin resistance.	Insertion of PCR product (primers 73-75; template: Plasmid 29) into NotI-EcoRI-cut Plasmid 26 using SLiCE cloning (Motohashi, 2015).
33	FLAG-cyclin B1 in pUHD-P1/Pur	Tet-Off plasmid expressing FLAG-tagged cyclin B1; puromycin resistance.	Ligation of BamHI-cut fragment containing puromycin-resistant gene from pUR5(BamHI) (a gift from Katsumi Yamashita, Kanazawa University, Japan) into BamHI-cut FLAG-cyclin B1 in pUHD-P1 (Fung et al., 2005).

Journal of Cell Science • Supplementary information

34	FLAG-cyclin B1 in pUHD-P1	Tet-Off plasmid expressing FLAG-cyclin B1.	Ligation of NcoI-BamHI-cut double PCR product (first PCRs: primers 76-77 and 78-79, respectively; template: Plasmid 33; second PCR: primers 76-79; then cut with NcoI-BamHI) into NcoI-BamHI-cut pUHD-P1 (Yam et al., 1999).
35	FLAG-3C-cyclin B1(NΔ88) in pUHD-P3	Tet-Off plasmid expressing FLAG-tagged cyclin B1(NΔ88).	BamHI-NcoI-cut PCR product (primer 80-81; template: Plasmid 34) was ligated into BamHI-NcoI-cut pUHD-P3 (Ma et al., 2009) .
36	FLAG-3C-cyclin B1(NΔ88)-DD in pDBEcR-pIND(SP1)-DD/Bla	Ecdysone-inducible plasmid with SB ITRs for expressing cyclin B1(NΔ88)^{DD} and DBEcR (3xmyc-tagged); blasticidin resistance.	Insertion of PCR product (primers 82-83; template: Plasmid 35) into NheI-EcoRI-cut Plasmid 31 using SLiCE cloning (Motohashi, 2015).
37	FLAG-3C-cyclin B1(NΔ88)-DD in pDBEcR-pIND(SP1)-DD-W/Bla	Ecdysone-inducible plasmid with SB ITRs for expressing cyclin B1(NΔ88)^{DD} (WPRE in 3' UTR) and DBEcR (3xmyc-tagged); blasticidin resistance.	Insertion of PCR product (primers 82-83; template: Plasmid 35) into NheI-EcoRI-cut Plasmid 32 using SLiCE cloning (Motohashi, 2015).
38	pSBbi-TIR1/Hyg	Plasmid with SB ITRs for expressing TIR1; hygromycin resistance.	Ligation of EcoRI-cut PCR product (primers 84-85; template: pUHD-SB-mAID/Hyg (Yeung et al., 2021)) into EcoRI-cut pSBbi-TIR1 (Lau et al., 2021).
39	pSBbi-3xHA/Hyg	Cloning intermediate.	Insertion of PCR product (primers 86-87; template: pAc5 HA3-eDHFR-T2A-puro (a gift from David Bentley; Addgene #86395)) into NcoI-HindIII-cut Plasmid 38 using SLiCE cloning (Motohashi, 2015).
40	pSBbi-3xFLAG-3xmyc/Pur	Cloning intermediate.	Insertion of PCR product (primers 88-89; template: 3xFLAG-ENSA in pIRES-Puro (a gift from Tim Hunt, Cancer Research UK)) into NcoI-SalI-cut pSBbi-TIR1/Pur (Yeung et al., 2021).
41	pSBbi-Clover-TIR1/Pur	Cloning intermediate.	Insertion of two PCR products ((1) primers 22-90; template: Plasmid 10; (2) primers 23-91) into NheI-EcoRI-cut pSBbi-LacI-VP48-TIR1/Pur (Lau et al., 2021) using SLiCE cloning (Motohashi, 2015).
42	pSBbi-3xFLAG-Clover-T2A-3xmyc/Pur	Cloning intermediate.	Insertion of PCR product (primers 92-93; template: Plasmid 41) into NcoI-cut Plasmid 40 using SLiCE cloning (Motohashi, 2015).
43	pSBbi-3xHA-NcoI-3xFLAG-NheI-Clover-EcoRI-T2A/Bla	Cloning intermediate.	Insertion of double PCR product (primers 94-95 and 96-97, respectively; template: Plasmid 42; second PCR: primers 94-97) and PCR product (primers 98-99; template: Plasmid 20) into EcoRI-cut Plasmid 39 using SLiCE cloning (Motohashi, 2015).
44	pSBbi-mRuby2-Clover/Bla	Plasmid with SB ITRs for expressing mRuby2 (3xHA-tagged) and Clover (3xFLAG-tagged); blasticidin resistance.	Insertion of PCR product (primers 100-101; template: pAC1404-pCR8-mRuby2_NLSPUFa (a gift from Albert Cheng; Addgene #71902)) into NcoI-cut Plasmid 43 using SLiCE cloning (Motohashi, 2015).
45	pSBbi-TIR1/Pur	Plasmid with SB ITRs for expressing TIR1; puromycin resistance.	(Yeung et al., 2021).
46	CDK2 CRISPR-Cas9 in pX330	CDK2 CRISPR-Cas9.	(Ng et al., 2019).
47	pCMV(CAT)T7-SB100	SB transposase.	A gift from Zsuzsanna Izsvak; Addgene, #34879.

48	pCas9-sgAAVS1-1	Plasmid expressing sgAAVS1-1.	A gift from Elina Ikonen; Addgene #129726.
49	pCas9-sgAAVS1-2	Plasmid expressing sgAAVS1-2.	A gift from Elina Ikonen; Addgene #129727.
50	Histone H2B-GFP	Plasmid expressing histone H2B-GFP; blasticidin resistance.	A gift from Geoffrey Wahl (The Salk Institute for Biological Studies).
51	pUHD-P3T/PUR	Puromycin resistance.	(Ma et al., 2009).
52	mAID-CDK2 in pUHD-SB-mAID/Bla	Expression of ^{mAID}CDK2; blasticidin resistance.	Ligation of the NcoI-EcoRI fragment from FLAG-CDK2 in pUHD-P1 (Yam et al., 2000) into pUHD-SB-mAID/Bla (Yeung et al., 2021).
53	mAID-CDK2 in pUHD-SB-mAID/Pur	Expression of ^{mAID}CDK2; puromycin resistance.	Insertion of PCR product (primers 102-103; template: Plasmid 45) into MscI-HindIII-cut Plasmid 52 using SLiCE cloning.
54	pSBbi-AFB2-tTA/Neo	Vector with SB ITRs for expressing AFB2 and tTA; neomycin resistance.	Ligation of the XhoI-SalI fragment from Plasmid 5 into pSBbi-TIR1-tTA/Neo (Yeung et al., 2021).

References only found in this Table (other references can be found in the main reference list)

Fung, T. K., Yam, C. H. and Poon, R. Y. (2005). The N-terminal regulatory domain of cyclin A contains redundant ubiquitination targeting sequences and acceptor sites. *Cell Cycle* **4**, 1411-1420. doi:10.4161/cc.4.10.2046

Ma, H. T., Tsang, Y. H., Marxer, M. and Poon, R. Y. (2009). Cyclin A2-cyclin dependent kinase 2 cooperates with the PLK1-SCFβ-TrCP1-EMI1-anaphase promoting complex/cyclosome axis to promote genome reduplication in the absence of mitosis. *Mol. Cell. Biol.* **29**, 6500-6514. doi:10.1128/MCB.00669-09

Motohashi, K. (2015). A simple and efficient seamless DNA cloning method using SLiCE from Escherichia coli laboratory strains and its application to SLiP site directed mutagenesis. *BMC Biotechnol.* **15**, 47. doi:10.1186/s12896-015-0162-8

Xia, Y., Li, K., Li, J., Wang, T., Gu, L. and Xun, L. (2019). T5 exonuclease dependent assembly offers a low-cost method for efficient cloning and site directed mutagenesis. *Nucleic Acids Res.* **47**, e15. doi:10.1093/nar/gky1169

Yam, C. H., Ng, R.W., Siu,W. Y., Lau, A.W. and Poon, R. Y. (1999). Regulation of cyclin A-Cdk2 by SCF component Skp1 and F-box protein Skp2. *Mol. Cell. Biol.* **19**, 635-645. doi:10.1128/MCB.19.1.635

Journal of Cell Science • Supplementary information

Table S2. Oligonucleotides.

1	5'-CAGCTAGCATGGGCTTCTCTGAGACC-3'
2	5'-TTCCATGGAGCTTGTCTTCT-3'
3	5'-ACCGATCCAGCCTCCGCGGG-3'
4	5'-CTGGGTCATTACATTCTTTCTG-3'
5	5'-CAGAAAGAATGTAATGACCCAG-3'
6	5'-GCTCTGGCTAGTCCAAAGTCT-3'
7	5'-CTGGCCTCTGAGGCCACCATGAACTACTTTCC-3'
8	5'-CTAGCAGCAGAACCGGAGTCGACCAGAATCCAGA-3'
9	5'-TAATATTTTGTTAAAATTGGATCAGCCTAGAGAA-3'
10	5'-CGGACCGGTTACAAGCTTCAGAGGATCA-3'
11	5'-GTAACCGGTCCGTCGACCACTAGTTAGAGC-3'
12	5'-AGTGAGCGAGGAAGCGATCAGTAGTCAAGA-3'
13	5'-GCACCGGTTCAATTGCC-3'
14	5'-GTCTCGAGTAGCTAGTTCATGGC-3'
15	5'-CATCCAGCAGAGCGGTAGTGGAGAGGGC-3'
16	5'-CCCAGTTTAATTTAAATAGAGTTCATGGCAG-3'
17	5'-TTTAAGCTTGATATCCATGGTGGCCTC-3'
18	5'-TCGCTAGCAGAGGTTTCTAC-3'
19	5'-CTGTAGAAACCTCTGCTAGCACCATGCCAGAG-3'
20	5'-GCCCTCTCCACTACCGCTCTGCTGGATG-3'
21	5'-AAGGATCCACCGGTAATGGTGAGCAAGGGC-3'
22	5'-CCTCTCCACTACCAGACTTGTACAGCTCGTCCAT-3'
23	5'-AAGTCTGGTAGTGGAGAGGGCA-3'
24	5'-TTGCGGCCGCATCGATGGGCCAGGATTCTCC-3'
25	5'-AAAACTAGAGATTCTTGTTTAAGCTATCTAGTAGGC-3'
26	5'-GCCACCATGGATATCAAGCTTGGGGATCAATTCTCTA-3'
27	5'-GCTGAATTCTAGGCCCAGCTGGTT-3'
28	5'-GGTACTAGTAGAGGTTTCTACAG-3'
29	5'-AGAGAATTGATCCCCAAGC-3'
30	5'-CCTGTAGAAACCTCTCGCGATGGCCAAGTTGA-3'
31	5'-CAGAACATTTCTCTACTAGTTCATGGCAGCCAGCA-3'
32	5'-AACGGATCCACTAGCGACGAGCTGTACAAG-3'
33	5'-AGCAGAGGTTTCTACGGTCAACTTGGCCAT-3'
34	5'-ATGGCCAAGTTGACCGTAGAAACCTCTGCT-3'
35	5'-CACAAACGAGTTCATGATGCCC-3'
36	5'-GGGCATCATGAACTCGTTTGTG-3'
37	5'-ATCAGCGAGCTCTAGTCCATGCCATGTGTA-3'
38	5'-AGTCATCGATGGCCAAGCCTTTGT-3'
39	5'-TCGAATTCGGCCACGAAGTGCT-3'
40	5'-AAGAATTCGGTCGACCCTAGAGGCT-3'
41	5'-ATGGACTAGTCAGAGGCCAGC-3'
42	5'-AAGCTAGCATGGTGAAACCAGT-3'
43	5'-TCCACTACCAGACTTCCCATTCAGAT-3'
44	5'-CATAAGCTTCGGCCACGAAGTGCT-3'
45	5'-AAGACTTGAACGGATCCGGATCCGGAGGCT-3'
46	5'-TAGAGAATTGATCCCCAAGCTTAATGCAG-3'
47	5'-TGTCGAGATCAAACGGGC-3'
48	5'-ACCACTTGCGGCCGTATCT-3'
49	5'-GTAGATACGGCCGCAAGTGGTGA-3'
50	5'-TCCACTACCAGAATGCAGCTTGA-3'
51	5'-GGCTGATCAGCGAGCTCTA-3'
52	5'-GCTGCATTCTGGTAGTGGA-3'
53	5'-CTGGTAGTGGCTCTACAAGCGGATC-3'
54	5'-TTGAGACAAAGGCTTGGC-3'
55	5'-GATCCGCTTGTAGAGCCACTACCAG-3'
56	5'-CTCCACTACCAGACTTGCTAGTGGAT-3'
57	5'-AAGTATGCCATGGTGGCCTCAGA-3'

Journal of Cell Science • Supplementary information

58	5'-AGCCATGGGTTCTGCCGCAAGTGGT-3'
59	5'-CGGAATGCGTCATACAGGAGCCCAGCAA-3'
60	5'-ACTAGCAGCAGAACCGGATCCCAGCA-3'
61	5'-GCTGGCCTCTGAGGCCACCATG-3'
62	5'-TTGCTGGGCTCCTGTATGACGCATTCCG-3'
63	5'-AAGCTGGCCTCTGAGGCCAC-3'
64	5'-TTCACCACTTGCGGCAGAACC-3'
65	5'-AGCCTCTAGGGTCGACCTCGACGGAT-3'
66	5'-CCTGCACCTGAGGAGTGAATTCTTATCATGTCTG-3'
67	5'-AAGAATTCTCTTATCGGTAATCAACCT-3'
68	5'-TAGCTCTCAATTGCGACGGTATCG-3'
69	5'-CCTGCACCTGAGGAGTGAATGTCTGGATC-3'
70	5'-AGAAGAACTCACACACATCCAGCCTC-3'
71	5'-CCCCATCTTCGACTCGGAGTGCAGGTGGAAA-3'
72	5'-TACCGAGCTCGAATTCTAGGCTTCCGGTTTTAG-3'
73	5'-GACGAGCTGTACAAGGAATTCGGAGTGCAGGTG-3'
74	5'-TGCACCTGAGGAGTGAACTAGGCTTCCGGT-3'
75	5'-TGATTACCGATAAGACTAGGCTTCCGGT-3'
76	5'-AAGGATCCGGGACCGATCCAGCC-3'
77	5'-AGGTTCGGGTCGGCTCCATCTT-3'
78	5'-AAGATGGAGCCGACCCGAACCT-3'
79	5'-TATCTTATCATGTCTGGATCC-3'
80	5'-GTACCCATGGTGGTGCCAGTGCC-3'
81	5'-CGGATCCTTACACCTTTGCCAC-3'
82	5'-AGAAGAACTCACACACAGCTAGCCACAATG-3'
83	5'-TTCCACCTGCACTCCGAACACCTTTGCCA-3'
84	5'-AGGAATTCGATGGATAGATCCG-3'
85	5'-GCGAATTCTATTCCTTTGCCC-3'
86	5'-CTGGCCTCTGAGGCCACCATGTACCCATACGA-3'
87	5'-CTAGAGAATTGATCCCCATGGTCATGATATCAG-3'
88	5'-CTGGCCTCTGAGGCCACGATGGATTA-3'
89	5'-CCACTAGCAGCAGAACCCATGGCGGTTTTATC-3'
90	5'-CCCTGTAGAAACCTCTGATGGTGAGC-3'
91	5'-ATAGGCAGCCTGCACCTGAGGAG-3'
92	5'-GATGACGATAAAACCGCGCTAGCCATGGTGAGCAA-3'
93	5'-CCACTAGCAGCAGAACCGTCGACCATCGATGGG-3'
94	5'-CTGTAGAAACCTCTGCAACATGGATTA-3'
95	5'-CTCACCATTGCTAGCGCGGTT-3'
96	5'-AACCGCGCTAGCAATGGTGAG-3'
97	5'-CGGATCCGAATTCCTTGTACAGCTC-3'
98	5'-AGGAATTCGGATCCGGAGAGGGCAGAGGAA-3'
99	5'-CCTGCACCTGAGGAGTGCTTAGCCC-3'
100	5'-TACGCTGATATCATGACCATGGTGTCTA-3'
101	5'-TCTAGAGAATTGATCCCGTCGACCCCGCTAC-3'
102	5'-TCTCTCTTTCCCTGTAGAAACC-3'
103	5'-AGAATTGATCCCCAAGCTTAATCAGGCACCG-3'
